# Enhancing Patient Comprehension of Glomerular Disease Treatments Using ChatGPT

**DOI:** 10.3390/healthcare13010057

**Published:** 2024-12-31

**Authors:** Yasir H. Abdelgadir, Charat Thongprayoon, Iasmina M. Craici, Wisit Cheungpasitporn, Jing Miao

**Affiliations:** Division of Nephrology and Hypertension, Department of Medicine, Mayo Clinic, Rochester, MN 55905, USA; abdelgadir.yasir@mayo.edu (Y.H.A.); thongprayoon.charat@mayo.edu (C.T.); craici.iasmina@mayo.edu (I.M.C.); cheungpasitporn.wisit@mayo.edu (W.C.)

**Keywords:** accuracy, ChatGPT, glomerular disorders, readability, accuracy, treatment options

## Abstract

**Background/Objectives**: It is often challenging for patients to understand treatment options, their mechanisms of action, and the potential side effects of each treatment option for glomerular disorders. This study explored the ability of ChatGPT to simplify these treatment options to enhance patient understanding. **Methods**: GPT-4 was queried on sixty-seven glomerular disorders using two distinct queries for a general explanation and an explanation adjusted for an 8th grade level or lower. Accuracy was rated on a scale of 1 (incorrect) to 5 (correct and comprehensive). Readability was measured using the average of the Flesch–Kincaid Grade (FKG) and SMOG indices, along with the Flesch Reading Ease (FRE) score. The understandability score (%) was determined using the Patient Education Materials Assessment Tool for Printable Materials (PEMAT-P). **Results**: GPT-4’s general explanations had an average readability level of 12.85 ± 0.93, corresponding to the upper end of high school. When tailored for patients at or below an 8th-grade level, the readability improved to a middle school level of 8.44 ± 0.72. The FRE and PEMAT-P scores also reflected improved readability and understandability, increasing from 25.73 ± 6.98 to 60.75 ± 4.56 and from 60.7% to 76.8% (*p* < 0.0001 for both), respectively. The accuracy of GPT-4’s tailored explanations was significantly lower compared to the general explanations (3.99 ± 0.39 versus 4.56 ± 0.66, *p* < 0.0001). **Conclusions**: ChatGPT shows significant potential for enhancing the readability and understandability of glomerular disorder therapies for patients, but at a cost of reduced comprehensiveness. Further research is needed to refine the performance, evaluate the real-world impact, and ensure the ethical use of ChatGPT in healthcare settings.

## 1. Introduction

Glomerular disorders are a significant cause of chronic kidney disease [1], but their complexity, particularly in terms of treatment options, can create substantial challenge for patient comprehension. For patients with glomerular disease, slowing the progression of chronic kidney disease often depends on enhanced self-care practices. Effective clinical management of these conditions necessitates a shared decision-making process, where both physicians and patients collaboratively determine the course of treatment. This collaborative approach is predicated on patients having a comprehensive understanding of their disease conditions to engage meaningfully in their health management. Nevertheless, effective understanding of these treatments, which involves grasping the mechanisms of actions, side effects, and interactions of various medications, demands a solid understanding of renal pathophysiology, pharmacology, and immunology. Given the diverse pathophysiological processes involved in glomerular disorders as well as their complications and the various treatment options, providing explanations that are both clear and accurate can be quite difficult.

Patients often feel overwhelmed by the complexity of medical terminologies and treatment options, which can complicate communication and reduce the effectiveness of educational materials [2]. A study conducted at a single center in Singapore revealed that among 23 patients with glomerulonephritis, there was a notably low level of health literacy, especially in the domain of appraising health information [3]. Similarly, a study from a single Norwegian center involving 22 pre-dialysis or dialysis patients affected by glomerulonephritis found these individuals to have moderate health literacy [4]. In Taiwan, a study encompassing 21 patients with chronic kidney disease due to glomerulonephritis demonstrated that 34% of the participants had low scores in both self-care and disease knowledge [5]. Collectively, these studies indicate that health literacy among patients with glomerular diseases tends to be low. This scarcity in health literacy can lead to poor medication compliance and, potentially, worse health outcome [6,7]. Bridging this knowledge gap is critical for healthcare providers to improve patient understanding and support well-informed decision-making regarding treatment plans.

Artificial intelligence (AI), particularly large language models (LLMs) like ChatGPT, provides a promising solution to addressing this knowledge gap. These models can translate complex medical terms and concepts into simpler, more understandable language, enabling patients to gain a clearer understanding of their glomerular diseases and treatment options. In the field of nephrology, our earlier studies have shown that ChatGPT notably enhances the readability of medical information regarding living kidney donation by reducing the complexity of the required reading level [8]. ChatGPT was also used to enhance readability by simplifying the literacy levels in responses across various topics. This included addressing questions on colon cancer [9], epilepsy and physical exercise [10], and traumatic dental injuries [11]. Additionally, it was employed in rewriting patient education material on dry eyes [12], rewriting abstracts in the neurosurgical literature [13], and providing peri-operative information for patients with a stoma [14]. This improvement in readability also extends to other medical specialties, as evidenced by research in areas including alveolar bone grafting [15], endoscopic skull base surgery [16], facial plastic and reconstructive surgery [17], radiology [18], cancer [19], and obstructive sleep apnea [20]. However, many previous studies did not investigate the accuracy of the information after simplification by ChatGPT, underscoring the importance of providing patients with both readable and accurate information.

In this study, we assessed both the readability and accuracy of medical explanations about treatment options for various glomerular disorders, as generated by ChatGPT. This dual assessment aims to ensure that while the information is made more accessible by lowering the reading level, it also remains precise and reliable.

## 2. Methods

### 2.1. Study Design

This study targets glomerular diseases that are commonly seen in clinical settings, aiming to cover a broad spectrum of these conditions while excluding very rare disorders. As such, we have chosen 67 glomerular disease terms (Table 1) spanning various etiologies, including glomerular diseases associated with nephrotic syndrome, glomerular diseases associated with nephritis, glomerular diseases associated with complement disorders, paraprotein-mediated glomerular diseases, hereditary glomerular disorders, and other miscellaneous conditions. This choice facilitates a focused but comprehensive examination of the critical elements of glomerular disease.

ChatGPT is available both as a subscription model (such as GPT-4) and a non-subscription version (GPT-3.5). Numerous studies have shown that GPT-4 surpasses GPT-3.5 in answering a range of examination questions [21,22,23,24,25,26,27,28,29,30], including nephrology [31]. They have also demonstrated GPT-4’s enhanced accuracy and readability over GPT-3.5 across various contexts including address questions and creating or revising patient education materials [9,10,11,12,13,14]. Our previous research has also indicated that GPT-4 is more effective in lowering the readability grade levels of living kidney donation information compared to GPT-3.5 [8]. Therefore, in our present study, we focused solely on evaluating GPT-4’s ability to enhance the readability of the answers to glomerular disease therapy.

Initial inquiries were made using the standard question, “What are the medications commonly used to treat [insert glomerular disease term]? Please also describe their mechanisms of action and potential side effects.” This was followed by a specialized request “Please modify the above responses for patients at or below an 8th-grade level.” To thoroughly assess ChatGPT’s capabilities, we did not perform any refinement process, such as training or feedback adjustments to its responses. Moreover, to maintain the objectivity and consistency of its performance, we initiated a new chat session for each distinct case of glomerular disease. This approach ensures that the evaluation metrics are not influenced by prior prompts or interactions.

### 2.2. Accuracy Assessment

As reported in other studies [32,33,34,35,36,37], the accuracy of the responses generated by GPT-4 was independently evaluated by two researchers (the first author, who is completing a clinical nephrology fellowship, and the corresponding author, a nephrology consultant). They used the same evaluation criteria: 1 (completely incorrect), 2 (mostly incorrect), 3 (partially correct and partially incorrect), 4 (correct but incomplete), and 5 (both correct and comprehensive). The average of the scores assigned by the two researchers was calculated for each response to determine its accuracy. Additionally, we conducted a consistency analysis between the two raters using Cohen’s kappa coefficient (https://www.graphpad.com/quickcalcs/kappa1/?K=5, accessed on 20 August 2024).

### 2.3. Readability Assessment

The readability of each response from GPT-4 was evaluated using two widely recognized tools in educational and health contexts: the Flesch–Kincaid Grade (FKG) Level and the Simple Measure of Gobbledygook (SMOG) Index. These metrics are available through publicly accessible readability calculators (https://readabilityformulas.com/readability-scoring-system.php#formulaResults, accessed on 1 August 2024), providing scores ranging from grade 1 to grade 16. This study presents the average of the FKG and SMOG scores to accurately determine the readability level.

Additionally, the Flesch Reading Ease (FRE) score was utilized. The FRE score ranges from 0 to 100, where higher scores indicate texts that are easier to read. Typically, texts scoring between 80 and 70 are considered suitable for seventh-grade readers, while scores between 70 and 60 are appropriate for eighth- or ninth-grade readers.

### 2.4. Understandability Assessment

The understandability score (%) was determined using the understandability domain of the Patient Education Materials Assessment Tool for Printable Materials (PEMAT-P) [38]. Specifically, three topics were evaluated: *Content* (2 items, including “The material makes its purpose completely evident” and “The material does not include information or content that distracts from its purpose”), *Word Choice and Style* (3 items, including “The material uses common, everyday language”, “Medical terms are used only to familiarize audience with the terms. When used, medical terms are defined”, and “The material uses the active voice”), and *Organization* (4 items, including “The material breaks or “chunks” information into short sections”, “The material’s sections have informative headers”, “The material presents information in a logical sequence”, and “The material provides a summary”). Topics such as *Use of Numbers*, *Layout, and Design*, and *Use of Visual Aids* were excluded as they were not applicable to this study. ChatGPT’s responses were rated as Agree (1 point) or Disagree (0 point) on each item. The PEMAT understandability score (%) is calculated as the total points for Agree items divided by total possible points (Agree items plus Disagree items; its 9 in this study), multiplied by 100.

### 2.5. Statistical Analysis

Accuracy and readability levels are reported as the mean ± standard deviation. To compare the accuracy and readability between general and tailored explanations, a paired two-sided *t*-test was employed, with a *p*-value less than 0.05 considered statistically significant. The association between accuracy and readability scores was analyzed using Pearson’s correlation coefficient.

## 3. Results

The general and tailored explanations generated by GPT-4 are presented in Supplementary Table S1. The general explanation received a mean accuracy score of 4.57 ± 0.66, suggesting that these responses were nearly accurate and comprehensive. However, explanations tailored for patients with an 8th-grade reading level or lower showed a reduced mean accuracy score of 3.99 ± 0.39 (Table 2). This difference in accuracy scores was statistically significant (*p* < 0.0001). Additionally, the inter-rater reliability for the accuracy assessment was substantial, as indicated by a Cohen’s kappa coefficient of 0.671 (95% CI 0.495 to 0.744).

**Figure 1:**
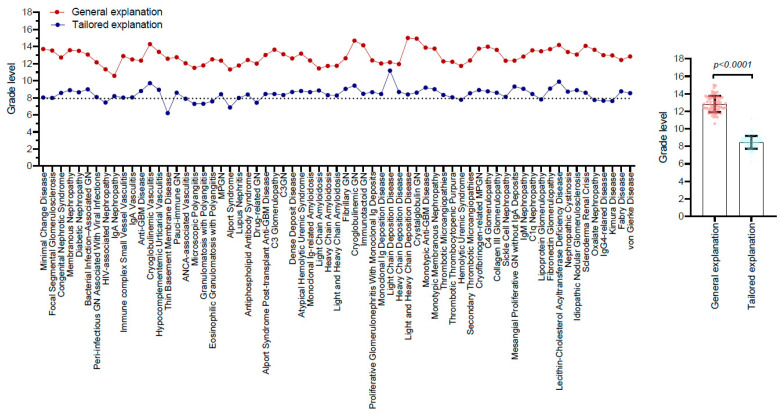
Readability grade levels of general and tailored explanations for glomerular disease therapies by ChatGPT. Grade levels were assessed using the Flesch–Kincaid Grade (FKG) Level and Simple Measure of Gobbledygook (SMOG) Index, and their averages are used in this study. The grade levels for GPT-4’s explanations for each of the glomerular disease therapies are presented (**left** panel). The grade levels for the general and tailored explanations by GPT-4 are compared using paired *t*-tests (**right** panel).

**Figure 2:**
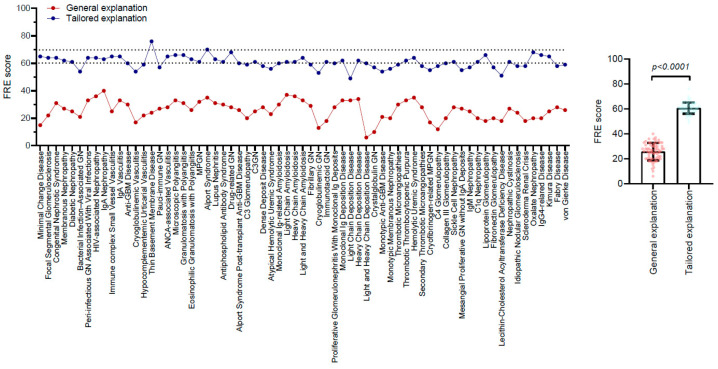
Flesch Reading Ease scores of general and tailored explanations for glomerular disease therapies by ChatGPT. The Flesch Reading Ease (FRE) scores for GPT-4’s explanations for each of the glomerular disease therapies are presented (**left** panel). The FRE score ranges from 0 to 100, where higher scores indicate texts that are easier to read. Typically, texts scoring between 70 and 60 are appropriate for eighth- or ninth-grade readers. The FRE scores of the general and tailored explanations are compared using paired *t*-tests (**right** panel).

The readability analysis revealed that the grade level for general explanations averaged 12.85 ± 0.93. In contrast, the tailored explanations were designed for significantly easier comprehension, with an average grade level of 8.44 ± 0.72 (*p* < 0.0001) (Table 2, Figure 1). The FRE scores also reflected this distinction; general explanations recorded a challenging FRE score of 25.73 ± 6.98, while tailored explanations achieved a significantly more accessible FRE score of 60.75 ± 4.56 (*p* < 0.0001) (Table 2, Figure 2). Notably, the FRE scores for the tailored explanations regarding therapies for the 25 glomerular diseases remain below 60, encompassing conditions such as light chain deposition disease, lecithin-cholesterol acyltransferase deficiency disease, and cryoglobulinemic glomerulonephritis, among others (Table 3). Additionally, there was a moderate negative correlation between accuracy and readability scores (r = −0.445, *p* < 0.001), indicating that improvements in readability tended to correspond with decreases in accuracy.

Furthermore, we performed a PEMAT analysis to evaluate understandability (Table 4), which demonstrated a notable enhancement in the understandability scores, increasing from 60.70 ± 8.46 for general explanations to 76.82 ± 11.39 for tailored explanations. The points for the *Content* item showed no significant difference between general and tailored explanations, indicating that both approaches effectively served their purpose. However, the points for *Word Choice and Style* and *Organization* were significantly higher for tailored explanations compared to general ones, with values of 2.09 ± 0.77 vs. 1.36 ± 0.51 and 2.84 ± 0.77 vs. 2.08 ± 0.61, respectively (both *p* < 0.0001). This suggests that tailored explanations often relied on common vocabulary and simpler sentence structures, making them more accessible and easier for patients to understand.

## 4. Discussion

This study underscores the potential of LLMs, such as GPT-4, to adapt medical information to readability levels accessible to patients with varying levels of health literacy. By doing so, these models contribute to health care equity, particularly for patients with glomerular disorders.

Health literacy is closely linked to patient outcomes [39,40,41,42,43]. According to the 2003 National Assessment of Adult Literacy, only 12% of U.S. adults possess proficient health literacy skills, while approximately 22% and 14% have basic and below-basic health literacy levels, respectively [44]. The Literacy Project indicates that the average American reads at a 7th to 8th grade level [6,45,46], which can impede the effective delivery of medical information across diverse literacy levels [47,48,49].

ChatGPT is extensively utilized across different medical specialties to answer common questions about health issues. However, the readability of its responses can be problematic for patients. Research has found that GPT-4’s replies to common questions about strabismus and amblyopia had readability levels suitable primarily for those with a college education, reflected by an FKG score over 14 and an FRE score below 23 [50]. In the fields of plastic and aesthetic surgery, the information provided by GPT-4 also required a high level of literacy, with an FRE index of 33.6 [51]. Moreover, a study on pediatric vesicoureteral reflux indicated that while ChatGPT delivers accurate and high-quality information, its readability is low, with challenging FKG and SMOG scores (average scores of 15 and 19, respectively) and a low FRE score of 26 ± 12 [52]. In educating patients about melanoma, a systematic review analysis showed that AI language models are highly accurate, though their readability frequently surpasses recommended levels [53]. These findings highlight a clear need for making the information more accessible. However, research on ChatGPT’s effectiveness in enhancing the readability of patient education materials in nephrology is still limited.

Our present study indicates that the readability of the treatment information for glomerular disorders generated by GPT-4 improved markedly when tailored to lower literacy levels. The average readability score dropped from 12.8 to 8.4, while the FRE score increased from 25.73 to 60.75, indicating easier comprehension. Additionally, the PEMAT analysis revealed a significant improvement in the understandability of tailored explanations compared to general explanations, despite the generated content being similar between the two groups. Notably, a detailed item analysis showed that tailored explanations frequently used common vocabulary and simpler sentence structures, making them easier for patients to understand.

Nevertheless, for certain glomerular diseases, such as light chain deposition disease, lecithin-cholesterol acyltransferase deficiency disease, and cryoglobulinemic glomerulonephritis, readability improvement remains challenging. These conditions contain specialized terms that are difficult to simplify, as evidenced by their FRE scores remaining below 60. Further investigation and tailored explanations are necessary to understand these complex conditions.

In our previous study, we found that both GPT-3.5 and GPT-4 could decrease the readability level of information on living kidney donation. The average FRE scores were lowered from 9.6 to 7.7 and 4.3, respectively, with GPT-4 proving to be more effective [8]. Another study showed that using ChatGPT improved the readability of patient instructions for Mohs micrographic surgery, as indicated by a reduction in the FKG level from 8.6 to 6.0 and the SMOG index from 8.9 to 6.7. However, the study did not evaluate the accuracy of the original or ChatGPT-modified texts, nor did it assess how these changes in readability scores actually affected patient comprehension [54]. In a comprehensive study, 100 abstracts from prominent neurology journals and 340 patient education documents from neurosurgical associations were rephrased by GPT-4 to target a 5th-grade reading level. This adjustment resulted in a significant readability improvement: the FKG level dropped from the 12th grade to the 5th for abstracts and from the 13th grade to the 5th for patient education materials, while largely preserving the content’s integrity [55]. Further analysis of 150 original abstracts of neurosurgical literature revealed a marked readability enhancement after GPT-4’s modifications, with the FKG level decreasing from 12.5 to 7.7, the SMOG index from 11.3 to 6.6, and the FRE score improving from 33.3 to 69.6. However, only 84.2% of the summaries maintained moderate scientific accuracy, as assessed by two physicians [56]. These results underscore the potential of AI to address low health literacy, though expert review remains essential.

Despite the improvement in readability and understandability, our study also noted a decrease in accuracy with more simplified texts, revealing a moderate negative correlation between readability and accuracy. Recently, multiple studies have discovered that while ChatGPT has simplified information across various medical fields, making it more readable, it has led to a reduction in comprehensiveness. These fields include cardiovascular disease prevention, dermatology educational materials for the public, and patient-centered information on breast cancer prevention and screening [57,58,59]. More detailed explanations tend to be more accurate but more challenging for many patients, particularly those with lower health literacy. Conversely, while simplified text improves readability and comprehension, there is often a loss in specificity and accuracy. The balance between accuracy and readability is crucial. Based on the average education level of the general population, a target readability at around the 8th-grade level is beneficial, making medical information significantly more readable, particularly for patients facing complex diseases and treatment options. Utilizing readability formulas such as the SMOG index, FKG, and FRE score can guide the process of tailoring educational materials to meet the needs of all patients effectively.

We acknowledge that this study has some limitations. While this study concentrated on critical aspects regarding treatment options and their underlying mechanisms and side effects from the patient’s perspective, the applicability of AI extends beyond just glomerular disease and its treatments; exploring ChatGPT’s ability to simplify other relevant patient concerns in future research could be valuable. Potential areas of interest include understanding the disorder itself, its pathophysiology, diagnostic processes, non-medical management strategies, outcomes of the disorders, implications of not receiving the medical treatment, and the impact on quality of life during treatment. Additionally, readability formulas, while common in various fields, may not fully capture the complexity of medical terminology or the nuanced comprehensiveness required in medical content. Setting a universal readability standard, such as the eighth-grade level, might not be suitable for everyone due to varying literacy skills within populations. Future research should focus on personalized educational approaches that account for individual literacy levels to enhance effectiveness. Moreover, we emphasize the lack of validation with end users or patients in our study, including the assessments of differences in knowledge of the disease and affective perceptions. It is crucial to determine whether simplified content effectively improves patient health outcomes, comprehension, knowledge retention, satisfaction, and adherence. Furthermore, the potential inaccuracies in LLM-generated content highlight the need for strict quality control, including reviews and validations by healthcare professionals. In the evolving landscape of healthcare, the integration of AI technologies like ChatGPT alongside physicians will bring the potential to enhance patient education significantly, especially in scenarios where there is a shortage of qualified medical professionals. But the role of physician and AI should be clarified and emphasized during this process. As physicians, they must maintain their pivotal role in delivering personalized, empathetic medical care and making complex health decisions. Their irreplaceable expertise and the trust they build with patients are fundamental for effective education and support. On the other hand, AI can greatly extend the reach and efficiency of patient education through various ways, such as offering instant access to a broad range of medical knowledge, providing personalized educational content at scale, and staying continuously updated with the latest medical knowledge, guidelines, and research. The future will likely see a synergistic relationship where AI supports and amplifies the efforts of physicians. By handling routine educational tasks and data management, AI can alleviate some of the workload from physicians, allowing them to dedicate more time to direct patient care and complex medical challenges [60]. This collaborative approach could lead to more thorough and accessible patient education, improved adherence to treatment plans, and, ultimately, better health outcomes. In future, optimizing the balance between technological efficiency and the nuanced, empathetic care provided by human physicians will be crucial for maximizing the benefits of both resources in patient education.

Future research could prioritize a more tailored approach that optimizes both readability and accuracy, exploring methods to maintain the precision of AI models while enhancing the readability of patient educational materials. Initially, it is crucial to refine user profiles within specific medical domains, such as nephrology. Exploring various prompt strategies could also increase accuracy; these include detailed inputs, chain-of-thought processing, and retrieval-augmented generation that integrates specific knowledge resources like clinical guidelines [61,62,63]. Additionally, direct feedback from patients could help tailor the complexity and presentation of information according to factors such as demographics, education levels, and health literacy. The use of a diverse human control group would provide a direct and authentic assessment of comprehension levels of the intended audience, thus improving the external validity of the findings. Expanding data analyses to include larger, more diverse datasets will further improve AI performance and ensure that the educational materials are sensitive and relevant across a wide patient population. Establishing ethical standards and oversight for using AI in patient education is critical. Ethical committees should conduct regular reviews, with a focus on privacy, consent, and transparency concerning AI-generated advice [64,65]. Additionally, integrating AI tools with existing electronic health record systems and patient portals could facilitate the seamless delivery of tailored educational content, thus improving patient engagement and comprehension of their health conditions. By prioritizing these areas, future projects can utilize AI tools more effectively to empower patients and enhance our understanding of the ethical and effective application of AI in healthcare.

## 5. Conclusions

In conclusion, GPT4 has successfully improved the readability and understandability of educational materials on therapies for glomerular disorders, demonstrating notable enhancements in readability and PEMAT scores through simplified explanations. However, these simplified explanations sometimes result in less comprehensive content, underscoring the importance of maintaining a balance between accuracy and readability. While tools like ChatGPT show great promise in enhancing patient education, further research is necessary to refine these approaches, evaluate their real-world impact, and ensure their ethical application. Cooperative efforts among researchers, medical professionals, patients, and advocates are essential to fully utilize AI technology in promoting equitable healthcare outcomes, particularly in the management of complex conditions such as glomerular diseases.

## 6. AI Disclosure

The use of ChatGPT in this study was strictly limited to the response-generating protocol described in the methods section. ChatGPT was not used for data analysis, writing, or any other aspects of the production of this manuscript.

## Figures and Tables

**Table 1:** List of glomerular disease terms assessed in this study.

No.	Glomerular Disease Terms
1	Minimal Change Disease
2	Focal Segmental Glomerulosclerosis
3	Congenital Nephrotic Syndrome
4	Membranous Nephropathy
5	Diabetic Nephropathy
6	Bacterial Infection–Associated GN
7	Peri-infectious GN Associated With Viral Infections
8	HIV-associated Nephropathy
9	IgA Nephropathy
10	Immune complex Small Vessel Vasculitis
11	IgA Vasculitis or Henoch-Schönlein Purpura
12	Anti-GBM Disease
13	Cryoglobulinemic Vasculitis
14	Hypocomplementemic Urticarial Vasculitis
15	Thin Basement Membrane Disease
16	Pauci-immune GN
17	ANCA-associated Vasculitis
18	Microscopic Polyangiitis
19	Granulomatosis with Polyangiitis
20	Eosinophilic Granulomatosis with Polyangiitis
21	Membranoproliferative Glomerulonephritis
22	Alport Syndrome
23	Lupus Nephritis
24	Antiphospholipid Antibody Syndrome
25	Drug-related GN
26	Alport Syndrome Post-transplant Anti-GBM Disease
27	C3 Glomerulopathy
28	C3 GN
29	Dense Deposit Disease
30	Atypical Hemolytic Uremic Syndrome
31	Monoclonal Immunoglobulin-related Amyloidosis
32	Light Chain Amyloidosis
33	Heavy Chain Amyloidosis
34	Light and Heavy Chain Amyloidosis
35	Fibrillary GN
36	Cryoglobulinemic GN (type I and type II)
37	Immunotactoid GN
38	Proliferative Glomerulonephritis With Monoclonal Ig Deposits
39	Monoclonal Ig Deposition Disease
40	Light Chain Deposition Disease
41	Heavy Chain Deposition Disease
42	Light and Heavy Chain Deposition Disease
43	Crystalglobulin GN
44	Monotypic Anti-GBM Disease
45	Monotypic Membranous Nephropathy
46	Thrombotic Microangiopathies
47	Thrombotic Thrombocytopenic Purpura
48	Hemolytic Uremic Syndrome
49	Secondary Thrombotic Microangiopathies
50	Cryofibrinogen-related MPGN
51	C4 Glomerulopathy
52	Collagen III Glomerulopathy
53	Sickle Cell Nephropathy
54	Mesangial Proliferative GN without IgA Deposits
55	IgM Nephropathy
56	C1q Nephropathy
57	Lipoprotein Glomerulopathy
58	Fibronectin Glomerulopathy
59	Lecithin-Cholesterol Acyltransferase Deficiency Disease
60	Nephropathic Cystinosis
61	Idiopathic Nodular Glomerulosclerosis
62	Scleroderma renal crisis
63	Oxalate nephropathy
64	Immunoglobulin G4-related Disease
65	Kimura Disease
66	Fabry Disease
67	von Gierke Disease

GN, glomerulonephritis; MPGN, membranoproliferative glomerulonephritis; GBM, glomerular basement membrane.

**Table 2:** Accuracy and readability assessment of GPT-4’s responses to glomerular disease terms.

	General Explanation (95% CI of Mean)	Tailored Explanation (95% CI of Mean) ^a^	*p* Value ^b^	Difference (95% CI) ^c^
Accuracy ^d^	4.56 ± 0.66 (4.41–4.73)	3.99 ± 0.39 (3.90–4.09)	<0.0001	0.57 (0.46–0.69)
Readability				
Grade level ^e^	12.85 ± 0.93 (12.62–13.07)	8.44 ± 0.72 (8.26–8.62)	<0.0001	4.41 (4.18–4.64)
FRE score ^f^	25.73 ± 6.98 (24.03–27.43)	60.75 ± 4.56 (59.63–61.86)	<0.0001	35.01 (33.29–36.74)

^a^ Tailored for patients at or below 8th grade level. ^b^ A paired two-sided *t* test. ^c^ Mean of differences and 95% confidence intervals (CIs). ^d^ Accuracy is scaled from 1 to 5: 1 = completely incorrect, 2 = mostly incorrect, 3 = partly correct and partly incorrect, 4 = correct but not comprehensive, and 5 = correct and comprehensive. ^e^ Grade level is the average of the Flesch–Kincaid Grade (FKG) Level and Simple Measure of Gobbledygook (SMOG) Index. ^f^ Flesch Reading Ease (FRE) scores range from 0 to 100, with higher scores indicating easier-to-read text.

**Table 3:** Flesch Reading Ease scores below 60 for tailored therapy explanations of 25 glomerular diseases.

Glomerular Disease	General Explanation	Tailored Explanation
Light Chain Deposition Disease	33	49
Lecithin-Cholesterol Acyltransferase Deficiency Disease	18	51
Cryoglobulinemic GN	13	53
Bacterial Infection–Associated GN	21	54
Cryoglobulinemic Vasculitis	17	54
Monotypic Anti-GBM Disease	21	54
Cryofibrinogen-related MPGN	17	55
Mesangial Proliferative GN without IgA Deposits	17	55
Atypical Hemolytic Uremic Syndrome	23	56
Monotypic Membranous Nephropathy	20	56
Pauci-immune GN	27	57
Crystalglobulin GN	10	57
IgM Nephropathy	25	57
Fibronectin Glomerulopathy	20	57
Dense Deposit Disease	28	58
Secondary Thrombotic Microangiopathies	28	58
C4 Glomerulopathy	12	58
Idiopathic Nodular Glomerulosclerosis	24	58
Scleroderma Renal Crisis	18	58
Fabry Disease	28	58
Hypocomplementemic Urticarial Vasculitis	22	59
C3 Glomerulopathy	20	59
Fibrillary GN	29	59
Thrombotic Microangiopathies	30	59
von Gierke Disease	26	59

GN, glomerulonephritis; MPGN, membranoproliferative glomerulonephritis; GBM, glomerular basement membrane.

**Table 4:** Understandability assessment of GPT-4’s responses to glomerular disease terms.

	General Explanation (95% CI of Mean)	Tailored Explanation (95% CI of Mean) ^a^	*p* Value ^b^	Difference (95% CI) ^c^
Understandability (%) ^d^	60.70 ± 8.46 (58.63–62.76)	76.82 ± 11.39 (74.04–79.59)	<0.0001	16.12 (14.42–17.82)
Content (Agree points) ^e^	2	2	na	na
Word Choice and Style (Agree points) ^e^	1.36 ± 0.51 (1.23–1.48)	2.09 ± 0.77 (1.90–2.28)	<0.0001	0.73 (0.61–0.86)
Organization (Agree points) ^e^	2.08 ± 0.61 (1.93–2.22)	2.84 ± 0.77 (2.69–2.99)	<0.0001	0.76 (0.63–0.89)

^a^ Tailored for patients at or below 8th grade level. ^b^ A paired two-sided *t* test. ^c^ Mean of differences and 95% confidence intervals (CIs). ^d^ Understandability score (%) is assessed using the Understandability domain of Patient Education Materials Assessment Tool for Printable Materials (PEMAT P), calculated by the total points/total possible points × 100. ^e^ Total points of Content = 2, total points of Word Choice and Style = 3, total points of Organization = 4. na: not applicable.

## Data Availability

The data underlying this article are available in this article and in its online supplementary material.

## Supplementary Materials

The following supporting information can be downloaded at https://www.mdpi.com/article/10.3390/healthcare13010057/s1: Table S1: General and tailored explanations of treatment options by ChatGPT for various glomerular disorders.
