# Supplementary material for: Enhancing Patient Comprehension of Glomerular Disease Treatments Using ChatGPT
Source: Healthcare (Basel). 2024 Dec 31;13(1):57. doi: 10.3390/healthcare13010057 (PMC11719585; doi:10.3390/healthcare13010057)
Supplement: Supplementary file 1 [file healthcare-13-00057-s001.zip › healthcare-3357654-supplementary.pdf]

Supplementary Table S1. General and tailored explanations of treatment options by ChatGPT for various glomerular disorders

No.	Glomerular disorders	General explanation	Tailored explanation
		**What are the medications often used for the treatment of xxxx? Please also describe their mechanisms of action and potential side effects.**	**Please modify the above responses for patients at or below an 8th-grade level.**
1	Minimal Change Disease	Minimal change disease (MCD) is primarily treated with corticosteroids, which are effective in inducing remission in the majority of cases. Here's a detailed overview of the medications commonly used, their mechanisms of action, and potential side effects:<br>### 1. **Corticosteroids (Prednisone, Prednisolone)**<br>- **Mechanism of Action:** Corticosteroids reduce inflammation and suppress the immune system by inhibiting multiple inflammatory cytokines and mediators. In MCD, they help reduce the permeability of the capillary walls in the kidneys, preventing protein leakage into the urine.<br>- **Side Effects:** Long-term use of corticosteroids can lead to weight gain, increased risk of infections, hypertension, hyperglycemia, osteoporosis, mood swings, and cataract formation.<br>### 2. **Calcineurin Inhibitors (Cyclosporine, Tacrolimus)**<br>- **Mechanism of Action:** These agents inhibit calcineurin, a protein phosphatase involved in activating T-cells of the immune system. This results in reduced cytokine production and diminished overall immune response, which is beneficial in treating MCD.<br>- **Side Effects:** Calcineurin inhibitors may cause nephrotoxicity, hypertension, neurotoxicity, gingival hyperplasia, and glucose intolerance.<br>### 3. **Mycophenolate Mofetil**<br>- **Mechanism of Action:** Mycophenolate mofetil inhibits inosine monophosphate dehydrogenase, which is crucial for the proliferation of T and B lymphocytes. This suppression of lymphocyte proliferation helps in reducing the immune response in MCD.<br>- **Side Effects:** Common side effects include gastrointestinal disturbances, potential development of leukopenia, and increased risk of infections.<br>### 4. **Rituximab (for refractory cases)**<br>- **Mechanism of Action:** Rituximab is a monoclonal antibody that targets CD20, a protein primarily found on the surface of B cells. By depleting B cells, rituximab helps in reducing the autoimmune response believed to be part of the MCD pathogenesis.<br>- **Side Effects:** Infusion reactions, risk of severe infections, and potential for reactivation of viral infections like hepatitis B.<br>### 5. **Angiotensin-Converting Enzyme (ACE) Inhibitors or Angiotensin II Receptor Blockers (ARBs)**<br>- **Mechanism of Action:** These are used adjunctively for their antiproteinuric effects. They lower blood pressure and decrease glomerular capillary pressure, thus reducing proteinuria.<br>- **Side Effects:** Can include elevated potassium levels, reduced kidney function, and dizziness.<br>### Considerations<br>- **Corticosteroids** are usually the first line of treatment, with treatment extending for several months to achieve remission.<br>- **Calcineurin inhibitors** are often used in steroid-resistant cases or in those where steroid side effects are intolerable.<br>- **Rituximab** is reserved for cases that do not respond to other therapies.<br>This comprehensive approach ensures a tailored treatment, aiming to maximize efficacy while managing potential side effects, which must be closely monitored throughout the therapy duration.	Certainly! Here's a simplified explanation of the medications used to treat minimal change disease, tailored for understanding at or below an 8th-grade level:<br><br>### 1. **Corticosteroids (like Prednisone)**<br>- **What They Do:** These medicines help stop swelling and calm down the immune system. They make the tiny filters in your kidneys let less protein slip into your urine.<br>- **Side Effects:** They can make you gain weight, feel moody, get infections more easily, have higher blood sugar, and possibly cause bone weakening.<br>### 2. **Calcineurin Inhibitors (like Cyclosporine and Tacrolimus)**<br>- **What They Do:** These drugs help quiet your immune system by stopping a specific action inside your immune cells, which helps prevent too much protein from getting into your urine.<br>- **Side Effects:** They can hurt your kidneys if used a lot, raise your blood pressure, make you feel shaky or cause your gums to grow too much.<br>### 3. **Mycophenolate Mofetil**<br>- **What They Do:** This medicine stops certain white blood cells from growing too fast, which helps calm your immune system.<br>- **Side Effects:** It can upset your stomach, lower your white blood cell count (making it easier to catch infections), and cause other stomach issues.<br>### 4. **Rituximab (for tough cases)**<br>- **What They Do:** This special medicine targets and removes a type of white blood cell that might be causing the disease. This helps calm down the immune system.<br>- **Side Effects:** It can cause reactions when getting the medicine, increase the risk of serious infections, and can reactivate old viruses like hepatitis B if they're in your body.<br>### 5. **Blood Pressure Medicines (ACE Inhibitors or ARBs)**<br>- **What They Do:** These are used along with other treatments to help lower the pressure in your kidneys, which helps reduce protein loss.<br>- **Side Effects:** They might make you feel dizzy, affect how your kidneys work, or increase potassium in your blood, which needs to be watched.<br>### Key Points:<br>- **Corticosteroids** are usually the first medicine doctors try, and you might take them for several months to get better.<br>- **Calcineurin inhibitors** are used if the first medicine doesn't work well or if it causes too many side effects.<br>- **Rituximab** is used when other medicines don't work.<br>Doctors watch closely how these medicines affect you to make sure they help without causing too many problems.
2	Focal Segmental Glomerulosclerosis	Focal Segmental Glomerulosclerosis (FSGS) is a kidney disease that involves scarring of the glomeruli, the small filtering units within the kidney. Treatment aims to control symptoms and	Sure, let's explain the treatments for Focal Segmental Glomerulosclerosis (FSGS) in a simpler way. This is a kidney condition where parts of the kidneys get scarred, and

		prevent further progression of the disease. Here are the commonly used medications for FSGS, along with their mechanisms of action and potential side effects:<br>### 1. **Corticosteroids** (e.g., Prednisone)<br>- **Mechanism of Action**: These drugs reduce inflammation and immune response by suppressing the activity of the immune system and decreasing the production of inflammatory chemicals.<br>- **Side Effects**: Weight gain, increased risk of infections, hypertension, mood swings, and bone thinning.<br>### 2. **Calcineurin Inhibitors** (e.g., Cyclosporine, Tacrolimus)<br>- **Mechanism of Action**: These agents work by inhibiting calcineurin, a protein phosphatase involved in activating T-cells of the immune system, thereby reducing the immune response and inflammation.<br>- **Side Effects**: Nephrotoxicity (can harm the kidneys), hypertension, tremor, increased risk of infections, and possible development of diabetes.<br>### 3. **Angiotensin-Converting Enzyme (ACE) Inhibitors** (e.g., Lisinopril, Enalapril)<br>- **Mechanism of Action**: ACE inhibitors help to lower blood pressure and reduce proteinuria (protein in urine) by blocking the conversion of angiotensin I to angiotensin II, which is a substance that can constrict blood vessels and increase blood pressure.<br>- **Side Effects**: Cough, elevated blood potassium levels, low blood pressure, and renal function deterioration, particularly in cases with bilateral renal artery stenosis.<br>### 4. **Angiotensin II Receptor Blockers (ARBs)** (e.g., Losartan, Irbesartan)<br>- **Mechanism of Action**: Similar to ACE inhibitors, ARBs reduce blood pressure and proteinuria by blocking the action of angiotensin II, allowing blood vessels to widen.<br>- **Side Effects**: Dizziness, elevated blood potassium levels, and occasionally renal function deterioration.<br>### 5. **Rituximab**<br>- **Mechanism of Action**: Rituximab is a monoclonal antibody that targets CD20 protein on the surfaces of B-cells, leading to the depletion of these cells. It is used in cases resistant to standard therapy.<br>- **Side Effects**: Infusion reactions, increased risk of infections, progressive multifocal leukoencephalopathy (rare but serious brain infection), and hepatitis B reactivation in carriers.<br>### 6. **Plasmapheresis**<br>- **Mechanism of Action**: Though not a medication, plasmapheresis is a procedure used in severe or refractory cases. It involves filtering the blood to remove harmful substances, including antibodies that may contribute to FSGS.<br>- **Side Effects**: Drop in blood pressure, bleeding due to anticoagulants used during the procedure, and possible infection.<br>### 7. **Mycophenolate Mofetil**<br>- **Mechanism of Action**: This medication inhibits the proliferation of B and T lymphocytes, thus suppressing the immune response.<br>- **Side Effects**: Diarrhea, vomiting, increased risk of infections, and possible development of anemia or leukopenia.<br>When considering these treatments, the specific choice and combination of medications depend on the individual's disease characteristics, response to previous treatments, and the presence of any co-existing conditions. Regular monitoring and adjustments by a healthcare provider are essential to manage the disease effectively and minimize side effects.	these treatments help manage it:<br>### 1. **Corticosteroids** (like Prednisone)<br>- **What They Do**: These medicines help reduce swelling and calm down the body's defense system to prevent it from attacking the kidneys.<br>- **Side Effects**: They can make you gain weight, get infections more easily, feel mood changes, or have weaker bones.<br>### 2. **Calcineurin Inhibitors** (like Cyclosporine, Tacrolimus)<br>- **What They Do**: These drugs help control the body's defense system so it doesn't harm the kidneys.<br>- **Side Effects**: They can cause kidney problems, high blood pressure, shaky hands, and increase the chance of getting sick.<br>### 3. **ACE Inhibitors** (like Lisinopril, Enalapril)<br>- **What They Do**: These help lower blood pressure and reduce protein loss in urine, which is good for kidney health.<br>- **Side Effects**: They might cause a dry cough, change blood pressure, or affect kidney function.<br>### 4. **Angiotensin II Receptor Blockers (ARBs)** (like Losartan, Irbesartan)<br>- **What They Do**: Similar to ACE inhibitors, these also help with high blood pressure and reduce protein in urine by relaxing blood vessels.<br>- **Side Effects**: They can make you feel dizzy and affect kidney function.<br>### 5. **Rituximab**<br>- **What They Do**: This medicine targets certain cells of the immune system to protect the kidneys from being damaged by the body's own defense system.<br>- **Side Effects**: It can cause reactions during treatment, increase the risk of infections, and other serious conditions.<br>### 6. **Plasmapheresis**<br>- **What It Does**: This isn't a medicine but a special procedure that cleans the blood of harmful substances that can hurt the kidneys.<br>- **Side Effects**: It might cause low blood pressure, bleeding, or infections.<br>### 7. **Mycophenolate Mofetil**<br>- **What It Does**: This medicine helps control the immune system to prevent it from damaging the kidneys.<br>- **Side Effects**: It can cause stomach upset, increase the risk of infections, and lead to blood issues like anemia.<br>Each treatment has a specific job in managing FSGS, and doctors choose the best one based on how the patient is doing and other health factors. Regular check-ups are important to make sure the treatments are working well and not causing other problems.
3	Congenital Nephrotic Syndrome	Congenital Nephrotic Syndrome (CNS) is a rare kidney disorder that typically presents in infancy, characterized by significant protein loss in the urine, leading to low blood protein levels and swelling. Management of CNS often involves specific medications to control symptoms and address the underlying processes contributing to the disease. Here are some commonly used medications along with their mechanisms of action and potential side effects:	Sure, let's simplify the explanations of the treatments for Congenital Nephrotic Syndrome (CNS), a rare kidney condition seen in babies that causes them to lose a lot of protein through their urine, leading to swelling and other issues:<br><br>### 1. **Albumin and Water Pills (Diuretics)**<br>- **What They Do**: Albumin is a special protein given through a vein to help stop

		### 1. **Albumin and Diuretics**<br>- **Mechanism of Action**: Albumin is administered intravenously to replace proteins lost in the urine, helping to manage swelling and maintain blood volume. Diuretics help remove excess fluid from the body.<br>- **Side Effects**: Albumin can cause fluid overload if not carefully monitored. Diuretics can lead to dehydration, electrolyte imbalances, and kidney function deterioration.<br><br>### 2. **Angiotensin-Converting Enzyme (ACE) Inhibitors** (e.g., Enalapril, Captopril)<br>- **Mechanism of Action**: These medications help reduce protein loss in the urine and lower blood pressure by blocking the conversion of angiotensin I to angiotensin II.<br>- **Side Effects**: They can cause cough, high potassium levels, low blood pressure, and can be harmful to kidney function, especially in cases of significant pre-existing kidney impairment.<br><br>### 3. **Angiotensin II Receptor Blockers (ARBs)** (e.g., Losartan)<br>- **Mechanism of Action**: Similar to ACE inhibitors, ARBs also help reduce proteinuria and control blood pressure by blocking the effects of angiotensin II, thereby relaxing blood vessels.<br>- **Side Effects**: Dizziness, high potassium levels, and impacts on kidney function are potential side effects.<br><br>### 4. **Anticoagulants** (e.g., Warfarin)<br>- **Mechanism of Action**: These are used to prevent blood clots, which patients with CNS are at higher risk for due to altered blood protein levels affecting clotting factors.<br>- **Side Effects**: Increased risk of bleeding, requiring regular monitoring of blood clotting times to ensure safe levels.<br><br>### 5. **Immunosuppressants** (in cases caused by specific genetic mutations)<br>- **Mechanism of Action**: These drugs suppress the immune system to reduce inflammation and the immune response, potentially decreasing protein loss when CNS is part of a broader autoimmune or inflammatory process.<br>- **Side Effects**: Increased risk of infections, potential liver or kidney toxicity, and other systemic effects depending on the specific agent used.<br><br>### 6. **Antibiotics**<br>- **Mechanism of Action**: Prophylactic antibiotics may be used to prevent infections due to immunosuppression or the use of invasive lines for treatment.<br>- **Side Effects**: Antibiotic resistance, gastrointestinal disturbances, and allergic reactions can occur.<br><br>### 7. **Vitamin D and Calcium Supplements**<br>- **Mechanism of Action**: These supplements are often necessary due to the loss of vitamin D-binding proteins and calcium in the urine, which can lead to bone weakening.<br>- **Side Effects**: Excessive intake can lead to hypercalcemia (high blood calcium levels), which can cause various symptoms including nausea, vomiting, confusion, and kidney stones.<br><br>The treatment for CNS often requires careful monitoring and adjustments by healthcare providers to balance the management of symptoms with the minimization of potential side effects. Each treatment plan is tailored to the individual needs of the patient, taking into account the severity of the disease and the presence of any additional health issues.	swelling and keep blood levels healthy. Water pills help the body get rid of extra water by making more urine.<br>- **Side Effects**: Albumin can make the body hold too much water if not carefully watched. Water pills can make you dehydrated and disturb the balance of salts in your body.<br><br>### 2. **ACE Inhibitors** (like Enalapril, Captopril)<br>- **What They Do**: These medicines help lower the amount of protein lost in urine and help keep blood pressure normal by stopping a specific body chemical from being made.<br>- **Side Effects**: They might cause a dry cough, change the levels of potassium in the blood, lower blood pressure, and affect how well the kidneys work.<br><br>### 3. **Angiotensin II Receptor Blockers (ARBs)** (like Losartan)<br>- **What They Do**: These are similar to ACE inhibitors and help reduce the loss of protein in urine and lower blood pressure by making blood vessels relax.<br>- **Side Effects**: They can make you dizzy, change potassium levels in your blood, and affect kidney function.<br><br>### 4. **Blood Thinners (Anticoagulants)** (like Warfarin)<br>- **What They Do**: These medicines are used to prevent blood clots, which can happen more easily in CNS because of changes in blood proteins.<br>- **Side Effects**: They can increase the chance of bleeding, so it's important to regularly check how quickly your blood clots.<br><br>### 5. **Immune System Controllers (Immunosuppressants)**<br>- **What They Do**: These medicines help calm down the body’s defense system, which can sometimes help reduce the loss of protein in the urine.<br>- **Side Effects**: They can make it easier to get sick (infections) and might hurt the liver or kidneys.<br><br>### 6. **Antibiotics**<br>- **What They Do**: These are given to stop or prevent infections, especially because the treatments for CNS might make it easier to get sick.<br>- **Side Effects**: They can cause stomach upset, allergic reactions, or lead to germs that are hard to kill with medicine.<br><br>### 7. **Vitamin D and Calcium Supplements**<br>- **What They Do**: These are given because the body might lose important nutrients like calcium and vitamin D, which are needed for strong bones.<br>- **Side Effects**: Too much can cause high calcium levels, leading to stomach upset, confusion, and kidney problems.<br><br>For children with CNS, doctors carefully watch and change treatments as needed to help manage the condition while trying to prevent side effects. Each treatment is chosen based on how the child is doing and what is safest for them.
4	Membranous Nephropathy	Membranous Nephropathy (MN) is a kidney disorder characterized by the thickening of the membranes within the glomeruli—the tiny blood vessels in the kidneys that filter wastes from the blood. This condition is often associated with significant proteinuria (excessive protein in urine) and can lead to nephrotic syndrome. Treatment typically aims to reduce proteinuria,	Sure, let's simplify the explanations of the treatments for Membranous Nephropathy (MN), which is a kidney condition where the tiny filters in the kidneys (called glomeruli) get thick, making it hard for them to work properly and causing too much protein to go into the urine. Here are the common treatments and what

		manage symptoms, and prevent progression to kidney failure. Here's an overview of commonly used medications for MN, their mechanisms of action, and potential side effects:<br><br>### 1. **Corticosteroids** (e.g., Prednisone)<br>- **Mechanism of Action**: Corticosteroids reduce inflammation and suppress the immune system, decreasing the production of antibodies that can damage the membranes in the kidneys.<br>- **Side Effects**: Weight gain, increased risk of infections, hypertension, mood swings, increased blood sugar levels, and bone thinning.<br><br>### 2. **Calcineurin Inhibitors** (e.g., Cyclosporine, Tacrolimus)<br>- **Mechanism of Action**: These drugs suppress the immune system by inhibiting the activity of calcineurin, a protein important for the activation of immune cells, thereby reducing the immune response that contributes to kidney damage.<br>- **Side Effects**: Kidney toxicity, high blood pressure, tremors, increased risk of infections, and potential increase in blood sugar levels.<br><br>### 3. **Rituximab**<br>- **Mechanism of Action**: Rituximab is a monoclonal antibody that targets CD20 protein on the surfaces of B-cells, leading to the depletion of these cells. This reduces the production of antibodies that attack the kidney.<br>- **Side Effects**: Infusion reactions, increased risk of infections, progressive multifocal leukoencephalopathy (a rare brain infection), and hepatitis B reactivation in carriers.<br><br>### 4. **Alkylating Agents** (e.g., Cyclophosphamide)<br>- **Mechanism of Action**: These drugs alkylate or bind to DNA, disrupting the replication of cells, particularly those of the immune system, thus reducing the immune response against kidney tissues.<br>- **Side Effects**: Bone marrow suppression (leading to low blood counts), increased risk of infections, hair loss, and potential for bladder toxicity and secondary cancers.<br><br>### 5. **Angiotensin-Converting Enzyme (ACE) Inhibitors** (e.g., Lisinopril, Enalapril)<br>- **Mechanism of Action**: ACE inhibitors help to reduce proteinuria and manage blood pressure by blocking the conversion of angiotensin I to angiotensin II, thereby relaxing blood vessels and reducing pressure within the glomeruli.<br>- **Side Effects**: Dry cough, elevated blood potassium levels, low blood pressure, and in rare cases, kidney function impairment.<br><br>### 6. **Angiotensin II Receptor Blockers (ARBs)** (e.g., Losartan, Irbesartan)<br>- **Mechanism of Action**: ARBs also reduce proteinuria and lower blood pressure by blocking the effects of angiotensin II directly, which helps to relax blood vessels and decrease blood pressure.<br>- **Side Effects**: Similar to ACE inhibitors, including dizziness, high potassium levels, and occasionally impacting kidney function.<br><br>### 7. **Anticoagulants** (e.g., Warfarin)<br>- **Mechanism of Action**: These are used in some patients to prevent the formation of blood clots, which patients with MN are at higher risk of due to alterations in blood coagulation factors from significant protein loss.<br>- **Side Effects**: Increased risk of bleeding, requiring frequent monitoring and dose adjustments based on blood clotting tests.	they do:<br><br>### 1. **Corticosteroids** (like Prednisone)<br>- **What They Do**: These medicines help reduce swelling and calm down the body's defense system to keep it from harming the kidneys.<br>- **Side Effects**: They can make you gain weight, get infections more easily, raise your blood pressure, make you feel moody, increase your blood sugar, and make your bones weaker.<br><br>### 2. **Calcineurin Inhibitors** (like Cyclosporine, Tacrolimus)<br>- **What They Do**: These drugs help control the body's defense system by blocking a specific protein needed for the immune cells to work. This helps protect the kidneys from getting damaged.<br>- **Side Effects**: They can harm the kidneys, cause high blood pressure, make your hands shake, increase the chance of getting sick, and can also raise your blood sugar.<br><br>### 3. **Rituximab**<br>- **What They Do**: This medicine targets certain cells in the immune system and reduces their number, which helps in stopping them from attacking the kidneys.<br>- **Side Effects**: It can cause reactions during treatment, increase the risk of infections, and cause very rare but serious infections in the brain.<br><br>### 4. **Alkylating Agents** (like Cyclophosphamide)<br>- **What They Do**: These drugs interfere with how certain cells in the immune system copy themselves, which helps reduce the damage they can do to the kidneys.<br>- **Side Effects**: They can cause a decrease in blood cells which makes you more prone to infections and bleeding, hair loss, and can harm the bladder and increase the risk of other cancers later on.<br><br>### 5. **ACE Inhibitors** (like Lisinopril, Enalapril)<br>- **What They Do**: These medicines help lower the amount of protein lost in the urine and help keep blood pressure normal by stopping a body chemical from making the blood vessels tight.<br>- **Side Effects**: They might cause a dry cough, increase potassium in the blood, lower blood pressure, and sometimes affect how well the kidneys work.<br><br>### 6. **Angiotensin II Receptor Blockers (ARBs)** (like Losartan, Irbesartan)<br>- **What They Do**: These work like ACE inhibitors but block the body chemical directly, helping to relax blood vessels and lower blood pressure.<br>- **Side Effects**: They can cause dizziness, change potassium levels in your blood, and affect kidney function.<br><br>### 7. **Blood Thinners (Anticoagulants)** (like Warfarin)<br>- **What They Do**: These medicines are used to prevent blood clots that people with MN are at risk of getting because of changes in their blood proteins.<br>- **Side Effects**: They can increase the chance of bleeding, so it's important to check how quickly your blood clots regularly.<br><br>These treatments help manage the symptoms of MN and protect the kidneys from further damage. Each person may need a different combination of these medicines

		These medications are part of a broader therapeutic approach tailored to individual patient needs, severity of the disease, and underlying causes of MN. Regular monitoring by healthcare providers is essential to optimize treatment, manage potential side effects, and adjust therapy as needed to prevent progression of the disease.	based on how severe their condition is. Regular visits to the doctor are important to make sure the treatments are working and to adjust them if needed to keep side effects under control.
5	Diabetic Nephropathy	Diabetic Nephropathy is a form of kidney disease that affects individuals with diabetes. It's characterized by damage to the parts of the kidneys that filter blood due to high blood sugar levels. To manage this condition and prevent further kidney damage, several types of medications are commonly used. Here's a detailed look at these medications, their mechanisms of action, and potential side effects:<br><br>### 1. **Angiotensin-Converting Enzyme (ACE) Inhibitors** (e.g., Lisinopril, Enalapril)<br>- **Mechanism of Action**: ACE inhibitors work by blocking the enzyme that converts angiotensin I to angiotensin II, a powerful vasoconstrictor that also stimulates aldosterone secretion. This action reduces blood pressure, decreases blood volume, and lowers the workload on the heart, all of which are beneficial in protecting kidney function.<br>- **Side Effects**: Common side effects include a persistent dry cough, elevated blood potassium levels, fatigue, dizziness, and in rare cases, kidney function deterioration and swelling of tissues (angioedema).<br><br>### 2. **Angiotensin II Receptor Blockers (ARBs)** (e.g., Losartan, Irbesartan)<br>- **Mechanism of Action**: ARBs block the effects of angiotensin II at its receptor site, which helps relax blood vessels, thus lowering blood pressure and reducing kidney stress. They provide similar benefits to ACE inhibitors but without causing the cough associated with ACE inhibitors.<br>- **Side Effects**: These can include dizziness, hyperkalemia (high potassium levels), and occasionally kidney function impairment. They also carry a risk of fetal toxicity and should not be used during pregnancy.<br><br>### 3. **Diuretics** (e.g., Hydrochlorothiazide, Furosemide)<br>- **Mechanism of Action**: Diuretics help the kidneys remove excess fluid from the body, which helps control blood pressure and reduces swelling. They are often used in combination with other blood pressure medications.<br>- **Side Effects**: Potential side effects include dehydration, electrolyte imbalances (such as low potassium levels), increased urination, and, less commonly, kidney dysfunction.<br><br>### 4. **Glycemic Control Medications**<br>- **Mechanism of Action**: These medications aim to control blood glucose levels through various mechanisms, depending on the type of drug (e.g., metformin reduces glucose production in the liver, while insulin injections replace or supplement natural insulin).<br>- **Side Effects**: They vary widely depending on the medication but can include hypoglycemia (low blood sugar), weight gain (with insulin or sulfonylureas), gastrointestinal issues (with metformin), and potential cardiovascular effects.<br><br>### 5. **Sodium-Glucose Co-transporter 2 (SGLT2) Inhibitors** (e.g., Canagliflozin, Empagliflozin)<br>- **Mechanism of Action**: These drugs work by blocking the reabsorption of glucose in the kidneys, promoting its excretion in urine, which helps lower blood glucose levels and additionally provides cardiovascular and renal protective benefits.<br>- **Side Effects**: They can cause dehydration, urinary tract infections, and genital infections. There is also a risk of diabetic ketoacidosis with these medications, even if blood sugar levels are not very high.	Sure, let's explain the medications used to treat Diabetic Nephropathy, a kidney problem that happens in people with diabetes, in a simpler way:<br><br>### 1. **ACE Inhibitors** (like Lisinopril, Enalapril)<br>- **What They Do**: These medicines help relax the blood vessels, which makes it easier for the heart to pump blood and helps protect the kidneys from being damaged by high blood pressure.<br>- **Side Effects**: They can cause a dry cough, feel tired, dizzy, have too much potassium in the blood, and sometimes swelling.<br><br>### 2. **Angiotensin II Receptor Blockers (ARBs)** (like Losartan, Irbesartan)<br>- **What They Do**: These work a lot like ACE inhibitors but without causing a cough. They help lower blood pressure by relaxing blood vessels.<br>- **Side Effects**: They can make you dizzy, increase potassium in the blood, and are not safe to use during pregnancy.<br><br>### 3. **Diuretics** (like Hydrochlorothiazide, Furosemide)<br>- **What They Do**: These are often called water pills. They help the body get rid of extra water and salt through urine. This helps lower blood pressure and reduce swelling.<br>- **Side Effects**: They can cause dehydration, change the balance of salts in your body, make you pee a lot, and can affect kidney function.<br><br>### 4. **Medicines to Control Blood Sugar**<br>- **What They Do**: Different medicines help keep your blood sugar levels normal. Some decrease sugar production in the liver, and others help add insulin or make the body's insulin work better.<br>- **Side Effects**: They can cause blood sugar to go too low, weight gain, stomach upset, and other effects depending on the type.<br><br>### 5. **SGLT2 Inhibitors** (like Canagliflozin, Empagliflozin)<br>- **What They Do**: These medicines help lower blood sugar by making the kidneys get rid of sugar through urine. They also help protect the heart and kidneys from damage.<br>- **Side Effects**: They can cause dehydration, infections in the urinary tract or genital areas, and a rare condition where the body produces high levels of acids (ketoacidosis).<br><br>### 6. **Endothelin Receptor Antagonists** (like Atrasentan)<br>- **What They Do**: These drugs help protect the kidneys by blocking a substance in the blood that can make blood vessels narrow and cause more kidney damage.<br>- **Side Effects**: They can cause fluid to build up in the body, headaches, and might affect the liver.<br><br>Managing this kidney condition means taking these medicines to help keep blood sugar and blood pressure under control, as well as protecting the kidneys from further damage. It's important for people with this condition to see their doctor regularly to make sure the medicines are working right and not causing problems.

		### 6. **Endothelin Receptor Antagonists** (e.g., Atrasentan)<br>- **Mechanism of Action**: These drugs block endothelin, a substance that causes blood vessels to narrow and can worsen kidney damage. They are under investigation for their potential to protect the kidneys in diabetic nephropathy.<br>- **Side Effects**: Possible side effects include fluid retention, headache, and potential liver damage.<br><br>Management of diabetic nephropathy involves not only controlling blood sugar and managing blood pressure but also monitoring kidney function regularly to adjust treatments as needed and prevent further damage. Each medication regimen needs to be personalized, taking into account the overall health, kidney function, and specific needs of the patient. Regular follow-ups with healthcare providers are crucial to effectively manage the condition and adjust treatment plans based on the patient’s response and any side effects experienced.	Each person might need different medicines based on how their body responds and what their doctor thinks is best.
6	Bacterial Infection–Associated GN	Bacterial Infection-Associated Glomerulonephritis (GN) is a kidney condition that arises due to an immune response to bacterial infections, particularly those caused by certain strains of streptococcus bacteria. This response can lead to inflammation and damage to the kidney's filtering units, the glomeruli. Treatment typically targets the underlying bacterial infection and manages the immune response and inflammation in the kidneys. Here’s an overview of the commonly used medications for this condition:<br><br>### 1. **Antibiotics**<br>- **Examples**: Penicillin, Cephalexin, Dicloxacillin<br>- **Mechanism of Action**: Antibiotics work by killing bacteria or preventing them from growing. For example, Penicillin disrupts the bacterial cell wall synthesis, leading to the destruction of bacteria.<br>- **Side Effects**: Common side effects include allergic reactions, which can range from mild skin rashes to more severe reactions like anaphylaxis. Other side effects may include nausea, diarrhea, and yeast infections.<br><br>### 2. **Corticosteroids** (e.g., Prednisone)<br>- **Mechanism of Action**: These drugs reduce inflammation by suppressing the immune system’s activity. They inhibit various inflammatory cytokines and mediators that can cause damage to the glomeruli during an immune response.<br>- **Side Effects**: Long-term use can lead to weight gain, increased risk of infections, high blood sugar levels, mood swings, and osteoporosis.<br><br>### 3. **Immunosuppressants** (if the response to steroids is inadequate)<br>- **Examples**: Cyclophosphamide, Mycophenolate Mofetil<br>- **Mechanism of Action**: These medications suppress the immune system more broadly or selectively, reducing the production of immune cells and antibodies that contribute to kidney inflammation.<br>- **Side Effects**: Increased risk of infections, potential for kidney and liver toxicity, bone marrow suppression, and possible increased risk of cancers.<br><br>### 4. **Supportive Treatments**<br>- **Examples**: Blood pressure medications like ACE inhibitors or ARBs, Diuretics<br>- **Mechanism of Action**: These help manage symptoms of kidney dysfunction, such as high blood pressure and fluid overload. ACE inhibitors and ARBs specifically reduce proteinuria and control blood pressure, thereby minimizing further stress on the kidneys.<br>- **Side Effects**: Blood pressure medications may cause dizziness, elevated potassium levels, or kidney function impairment. Diuretics can lead to electrolyte imbalances and dehydration.	Sure, let’s break down the treatments for Bacterial Infection-Associated Glomerulonephritis (GN), a kidney problem that happens after a bacterial infection. This problem causes inflammation and damage to the tiny filters in the kidneys. Here’s how the medicines work:<br><br>### 1. **Antibiotics**<br>- **Examples**: Penicillin, Cephalexin, Dicloxacillin<br>- **What They Do**: These medicines fight off the bad bacteria causing the infection. Penicillin, for example, destroys the walls around the bacteria, which kills them.<br>- **Side Effects**: Sometimes, people might get allergic reactions like skin rashes or, very rarely, more severe reactions. Other common issues can include stomach upset and diarrhea.<br><br>### 2. **Corticosteroids** (like Prednisone)<br>- **What They Do**: These medicines help reduce swelling and calm down the body’s defense system to stop it from damaging the kidneys more.<br>- **Side Effects**: If used for a long time, they can cause weight gain, make it easier to catch infections, increase blood sugar, change moods, and make bones weaker.<br><br>### 3. **Immunosuppressants**<br>- **Examples**: Cyclophosphamide, Mycophenolate Mofetil<br>- **What They Do**: These are stronger medicines that help control the body's defense system by reducing the number of cells and substances that can cause inflammation and damage in the kidneys.<br>- **Side Effects**: These can make it much easier to get infections, and they might harm the kidneys, liver, and bone marrow. There’s also a small chance they could increase the risk of getting some types of cancer.<br><br>### 4. **Supportive Treatments**<br>- **Examples**: Blood pressure medicines like ACE inhibitors or ARBs, Water pills (Diuretics)<br>- **What They Do**: These help manage symptoms that come from kidney problems, like high blood pressure and swelling. ACE inhibitors and ARBs specifically help reduce protein loss in urine and control blood pressure.<br>- **Side Effects**: These medicines might make you dizzy, increase potassium in the blood, or affect how well the kidneys work. Water pills can cause an imbalance in body salts and dehydration.

		Treatment strategies for Bacterial Infection-Associated GN are primarily focused on eradicating the infection and controlling inflammation to prevent further kidney damage. It is crucial to manage this condition with regular monitoring by healthcare providers to adjust treatments as needed and address any emerging side effects.	The main goal of treating Bacterial Infection-Associated GN is to get rid of the infection and reduce inflammation to stop further damage to the kidneys. Doctors need to check on patients regularly to make sure the treatments are working and to adjust them if needed. This helps avoid any serious side effects and helps the kidneys heal.
7	Peri-infectious GN Associated With Viral Infections	Peri-infectious Glomerulonephritis (GN) associated with viral infections occurs when the body's immune response to a viral infection leads to inflammation and damage in the kidneys, particularly the glomeruli. Unlike bacterial infection-associated GN, the treatment for viral-associated GN often focuses more on managing symptoms and supporting kidney function, since direct antiviral therapies may not be as widely applicable or effective. Here’s a look at commonly used treatments, their actions, and potential side effects:<br><br>### 1. **Supportive Care**<br>- **Main Components**: This includes managing symptoms such as high blood pressure, swelling, and protein loss in urine. No specific antiviral medications are typically used unless a specific virus known to respond to such treatment is identified.<br>- **Mechanisms of Action**: The aim is to reduce workload on the kidneys, manage blood pressure, and reduce symptoms, helping to preserve kidney function while the body clears the virus.<br>- **Side Effects**: Generally, these treatments are well tolerated, but side effects depend on the specific medications used (such as blood pressure drugs or diuretics).<br><br>### 2. **Corticosteroids**<br>- **Examples**: Prednisone<br>- **Mechanism of Action**: Steroids are used to reduce immune system activity and decrease inflammation in the kidneys.<br>- **Side Effects**: Potential side effects include increased risk of infections, high blood sugar, weight gain, mood changes, and bone thinning with long-term use.<br><br>### 3. **Immunosuppressants**<br>- **Examples**: Cyclophosphamide, Mycophenolate Mofetil<br>- **Mechanism of Action**: These drugs suppress the immune response more broadly, which may be necessary if the inflammation does not resolve with supportive care and steroids.<br>- **Side Effects**: Increased susceptibility to infections, possible liver and kidney toxicity, bone marrow suppression, and an increased risk of certain cancers.<br><br>### 4. **Antiviral Therapy**<br>- **Applicability**: This is used if a specific treatable virus is identified (e.g., Hepatitis B or C, HIV).<br>- **Mechanism of Action**: Antiviral medications work by directly targeting the viral life cycle to prevent the virus from replicating and causing further damage.<br>- **Side Effects**: Side effects vary by drug but can include nausea, fatigue, headache, and more serious effects like liver damage or resistance to the medication.<br><br>### 5. **Plasmapheresis**<br>- **When Used**: Occasionally used in severe cases where there is significant immune complex deposition thought to be driving the kidney damage.<br>- **Mechanism of Action**: This procedure filters the blood to remove harmful immune complexes and antibodies.<br>- **Side Effects**: Can include low blood pressure, bleeding due to anticoagulants used in the process, and risk of infections.	Sure, let’s simplify the explanation of how doctors treat Peri-infectious Glomerulonephritis (GN), a kidney problem that happens because of a viral infection. This condition makes the kidneys swollen and damaged because the body is fighting off the virus. Here’s how the treatments work:<br><br>### 1. **Supportive Care**<br>- **What It Includes**: This means taking care of symptoms like high blood pressure, swelling, and protein in the urine. There usually aren't specific virus-fighting medicines unless the doctors find a virus that can be treated directly.<br>- **How It Works**: The goal is to make it easier for the kidneys to work and lower blood pressure, which helps the kidneys get better while the body deals with the virus.<br>- **Side Effects**: The side effects depend on what medicines are used, like those for blood pressure or water pills (diuretics).<br><br>### 2. **Corticosteroids** (like Prednisone)<br>- **What They Do**: These medicines help calm down the body’s defense system and reduce swelling in the kidneys.<br>- **Side Effects**: They can make it easier to catch infections, raise blood sugar levels, cause weight gain, affect your mood, and make your bones weaker if used for a long time.<br><br>### 3. **Immunosuppressants**<br>- **Examples**: Cyclophosphamide, Mycophenolate Mofetil<br>- **What They Do**: These drugs turn down the body's defense system even more if the swelling doesn’t go away with other treatments.<br>- **Side Effects**: They can make you more likely to get sick, can hurt the liver and kidneys, make your bone marrow less active, and increase the chance of getting certain cancers.<br><br>### 4. **Antiviral Therapy**<br>- **When It’s Used**: This is only used if doctors find a specific virus that they can treat with these medicines.<br>- **What It Does**: These medicines attack the virus directly to stop it from multiplying and causing more harm to the kidneys.<br>- **Side Effects**: Side effects can vary a lot but might include feeling sick to your stomach, feeling tired, headaches, and possibly harming the liver.<br><br>### 5. **Plasmapheresis**<br>- **When It’s Used**: Sometimes used in very bad cases to help clean the blood.<br>- **What It Does**: This treatment filters the blood to remove harmful substances that are causing kidney damage.<br>- **Side Effects**: Can cause low blood pressure, bleeding from the medicines that keep the blood from clotting during treatment, and possible infections.<br><br>The main goal of treating this kidney condition caused by viruses is to support the kidneys and manage symptoms while the body fights the virus. Doctors need to

		Management of peri-infectious GN associated with viral infections primarily aims at supportive care, with a focus on monitoring kidney function and addressing any specific viral causes when possible. The approach can vary significantly based on the type of virus involved and the severity of the kidney involvement. Regular follow-up with a healthcare provider is crucial to adjust treatment as needed and to monitor for complications or side effects from the therapies used.	keep a close eye on how well the kidneys are doing and change treatments as needed. Regular check-ups are important to make sure everything is going well and adjust the medicines to avoid side effects.
8	HIV-associated Nephropathy	HIV-associated Nephropathy (HIVAN) is a form of kidney disease that occurs due to direct infection of kidney cells by HIV and the resulting immune response. Managing HIVAN primarily involves controlling HIV infection and addressing the kidney damage it causes. Here's a detailed look at the common medications used for HIVAN, their mechanisms of action, and potential side effects:<br><br>### 1. **Antiretroviral Therapy (ART)**<br>- **Examples**: Tenofovir, Emtricitabine, and other combinations<br>- **Mechanism of Action**: ART is crucial for managing HIVAN as it suppresses HIV viral load, reduces kidney cell infection, and limits immune system activation. These drugs inhibit various steps in the HIV lifecycle, such as reverse transcription and integration into the host DNA.<br>- **Side Effects**: While ART is effective in controlling HIV, it can have side effects such as gastrointestinal issues (nausea, diarrhea), fatigue, and in rare cases, kidney damage, particularly with drugs like tenofovir. Regular monitoring of kidney function is required.<br><br>### 2. **Angiotensin-Converting Enzyme (ACE) Inhibitors** and **Angiotensin II Receptor Blockers (ARBs)**<br>- **Examples**: Lisinopril (ACE inhibitor), Losartan (ARB)<br>- **Mechanism of Action**: These drugs are used to manage hypertension and proteinuria in HIVAN. They work by blocking the effects of angiotensin II, a molecule that causes blood vessels to constrict and increases blood pressure. By relaxing blood vessels, they help reduce the workload on the kidneys.<br>- **Side Effects**: Common side effects include dizziness, increased blood potassium levels, and in rare cases, kidney function impairment. These drugs also carry a risk of causing angioedema (swelling similar to hives but under the skin).<br><br>### 3. **Diuretics**<br>- **Examples**: Furosemide<br>- **Mechanism of Action**: Diuretics are used to help the kidneys remove excess fluid from the body, which is important in managing swelling and hypertension associated with kidney damage.<br>- **Side Effects**: These can include dehydration, electrolyte imbalances, and increased urination.<br><br>### 4. **Corticosteroids**<br>- **Examples**: Prednisone<br>- **Mechanism of Action**: In some cases of HIVAN, corticosteroids may be used to reduce inflammation in the kidneys. They suppress the immune system and reduce inflammation throughout the body.<br>- **Side Effects**: Long-term use can lead to side effects such as weight gain, increased risk of infections, diabetes, and osteoporosis.<br><br>### 5. **Immunosuppressive Agents**<br>- **Examples**: Cyclosporine<br>- **Mechanism of Action**: These may be used in severe cases of HIVAN to further reduce the immune response and inflammation in the kidneys.	HIV-associated Nephropathy (HIVAN) is a type of kidney disease that can happen in people with HIV. It's important to treat both the HIV and the kidney problems it causes. Here's how the medicines work and what side effects they might have, explained simply:<br><br>### 1. **Antiretroviral Therapy (ART)**<br>- **Examples**: Tenofovir, Emtricitabine, and others<br>- **What They Do**: These medicines help control HIV by stopping the virus from growing inside the body. This helps protect the kidneys from damage caused by the virus.<br>- **Side Effects**: They can cause stomach upset like nausea or diarrhea, make you feel tired, and sometimes affect the kidneys. That's why doctors regularly check kidney health when you take these medicines.<br><br>### 2. **Blood Pressure Medicines (ACE Inhibitors and ARBs)**<br>- **Examples**: Lisinopril (ACE inhibitor), Losartan (ARB)<br>- **What They Do**: These medicines help lower blood pressure and reduce protein loss in urine, which is good for kidney health. They work by relaxing blood vessels.<br>- **Side Effects**: They might make you dizzy, increase potassium in your blood, and sometimes they can affect how well your kidneys work. There's also a small risk of swelling under the skin.<br><br>### 3. **Water Pills (Diuretics)**<br>- **Examples**: Furosemide<br>- **What They Do**: These help your body get rid of extra water and salt through urine. This helps with swelling and high blood pressure.<br>- **Side Effects**: They can cause dehydration, change the balance of salts in your body, and make you go to the bathroom a lot.<br><br>### 4. **Corticosteroids**<br>- **Examples**: Prednisone<br>- **What They Do**: These are used to reduce swelling in the kidneys in some cases. They work by calming down the body's defense system.<br>- **Side Effects**: If used for a long time, they can cause weight gain, make it easier to get infections, increase blood sugar, and make bones weaker.<br><br>### 5. **Immunosuppressive Agents**<br>- **Examples**: Cyclosporine<br>- **What They Do**: In severe cases, these medicines might be used to reduce the immune response even more to help protect the kidneys.<br>- **Side Effects**: They can lower your body's ability to fight infections and can cause other problems like kidney damage and high blood pressure.<br><br>### Supportive Treatments<br>- **Purpose**: Along with these medicines, doctors also take care of other things like making sure your blood pressure is good, managing minerals in your blood, and

		- **Side Effects**: These drugs can suppress the overall immune response, which increases the risk of infections and can have other side effects like kidney damage and high blood pressure.<br><br>### Supportive Treatments<br>- **Purpose**: In addition to specific medications, supportive treatments such as managing blood pressure, correcting electrolyte imbalances, and treating symptoms of kidney failure (like anemia and acidosis) are critical.<br><br>The management of HIVAN must be carefully coordinated, taking into account the overall health of the patient, HIV status, and kidney function. Regular follow-ups with healthcare providers are essential to monitor the effectiveness of the treatment and adjust it as needed, as well as to manage any side effects of the medications used.	treating other kidney problems like anemia.<br><br>Managing HIVAN involves using these medicines carefully and visiting the doctor regularly to check how the treatments are working and to make sure there are no serious side effects. This helps keep both HIV and kidney problems under control.
9	IgA Nephropathy	IgA Nephropathy, also known as Berger’s disease, is a kidney disorder where Immunoglobulin A (IgA) — an antibody produced by the immune system — deposits in the kidneys. This leads to inflammation that can damage kidney tissues. Treatment for IgA Nephropathy aims to slow the disease's progression and manage symptoms like high blood pressure and proteinuria (protein in urine). Here are commonly used medications, how they work, and their possible side effects:<br><br>### 1. **Corticosteroids** (e.g., Prednisone)<br>- **Mechanism of Action**: Corticosteroids help reduce inflammation and suppress the immune system. By doing so, they reduce the production of antibodies, including IgA, and decrease inflammation in the kidneys.<br>- **Side Effects**: Weight gain, increased risk of infections, high blood sugar, mood changes, and bone loss are common with long-term use.<br><br>### 2. **Immunosuppressants**<br>- **Examples**: Cyclophosphamide, Mycophenolate Mofetil, Azathioprine<br>- **Mechanism of Action**: These medications also suppress the immune system, reducing the production of the IgA antibodies that are depositing in the kidneys.<br>- **Side Effects**: Increased risk of infections, potential liver and kidney toxicity, hair loss, and gastrointestinal symptoms. There is also a risk of decreased bone marrow function and an increased risk of certain cancers.<br><br>### 3. **Angiotensin-Converting Enzyme (ACE) Inhibitors** and **Angiotensin II Receptor Blockers (ARBs)**<br>- **Examples**: Lisinopril (ACE inhibitor), Losartan (ARB)<br>- **Mechanism of Action**: These drugs help reduce blood pressure and proteinuria, which are common symptoms in IgA Nephropathy. They do this by relaxing blood vessels and reducing pressure in the glomeruli (kidney filtering units).<br>- **Side Effects**: Dizziness, elevated blood potassium levels, and reduced kidney function in some cases. They also can cause a dry cough (more common with ACE inhibitors).<br><br>### 4. **Fish Oil**<br>- **Mechanism of Action**: High-dose omega-3 fatty acid supplements (fish oil) have been used to try to reduce inflammation within the kidneys.<br>- **Side Effects**: Fish oil is generally well tolerated but can cause gastrointestinal symptoms such as belching, nausea, and a fishy aftertaste.<br><br>### 5. **Blood Pressure Medications** (Other than ACE inhibitors and ARBs)<br>- **Examples**: Calcium channel blockers, beta-blockers	IgA Nephropathy, also known as Berger’s disease, is when a type of protein called IgA builds up in the kidneys. This can cause inflammation and damage to the kidneys. Here’s how doctors treat this condition with medicines, explained simply:<br><br>### 1. **Corticosteroids** (like Prednisone)<br>- **What They Do**: These medicines help calm down the body’s defense system and reduce swelling in the kidneys.<br>- **Side Effects**: They can make you gain weight, more likely to get sick, have higher blood sugar, mood changes, and can make your bones weaker if used for a long time.<br><br>### 2. **Immunosuppressants**<br>- **Examples**: Cyclophosphamide, Mycophenolate Mofetil, Azathioprine<br>- **What They Do**: These drugs also help quiet the immune system to stop it from making too many IgA proteins that are hurting the kidneys.<br>- **Side Effects**: They can make it easier to catch infections, can hurt the liver and kidneys, cause hair loss, stomach upset, and there's a small chance they could lead to certain types of cancer.<br><br>### 3. **Blood Pressure Medicines (ACE Inhibitors and ARBs)**<br>- **Examples**: Lisinopril (ACE inhibitor), Losartan (ARB)<br>- **What They Do**: These medicines help keep your blood pressure low and reduce the amount of protein lost in the urine, which helps protect the kidneys.<br>- **Side Effects**: They might make you dizzy, increase the potassium in your blood, and sometimes affect how well your kidneys work. ACE inhibitors might also cause a dry cough.<br><br>### 4. **Fish Oil**<br>- **What It Does**: Fish oil can help reduce swelling in the kidneys because it has special fats that fight inflammation.<br>- **Side Effects**: It’s usually okay for most people, but it can cause an upset stomach, burping, and a fishy taste in your mouth.<br><br>### 5. **Other Blood Pressure Medicines**<br>- **Examples**: Calcium channel blockers, beta-blockers<br>- **What They Do**: These also help control your blood pressure, which is important for keeping your kidneys healthy.<br>- **Side Effects**: They can make you feel tired, cause your ankles to swell, constipation (mainly calcium channel blockers), or make your hands and feet cold

		- **Mechanism of Action**: These medications help manage blood pressure, which is crucial in slowing down kidney damage.<br>- **Side Effects**: Depending on the type, side effects can include fatigue, swollen ankles, constipation (calcium channel blockers), cold hands and feet, and sleep disturbances (beta-blockers).<br><br>### 6. **Anticoagulants** (if needed)<br>- **Examples**: Warfarin<br>- **Mechanism of Action**: Used in patients who develop blood clots, which can be a complication in severe kidney disease.<br>- **Side Effects**: Increased risk of bleeding, requiring regular monitoring of blood clotting times.<br><br>The treatment approach in IgA Nephropathy is often tailored based on individual symptoms, kidney function, and overall health. Regular monitoring is essential to adjust medications as necessary and manage any side effects that may occur. The goal is to protect kidney function as much as possible and prevent progression to more severe kidney disease.	and mess with your sleep (mainly beta-blockers).<br><br>### 6. **Blood Thinners** (if needed)<br>- **Examples**: Warfarin<br>- **What They Do**: These are used if there's a risk of blood clots, which can happen when the kidneys aren't working well.<br>- **Side Effects**: They can increase the chance of bleeding, so doctors need to check your blood regularly to make sure it's not too thin.<br><br>Doctors choose what medicines to use based on how the person is feeling and how their kidneys are doing. Regular check-ups are important to see if the treatments are working and to change things if needed. The main goal is to keep the kidneys working well and prevent the disease from getting worse.
10	Immune complex Small Vessel Vasculitis	Immune Complex Small Vessel Vasculitis (SVV) involves inflammation of the small blood vessels, typically caused by immune complexes (collections of immune cells and antibodies) that deposit in these vessels, leading to inflammation and damage. The treatment primarily focuses on suppressing the immune system to reduce inflammation and prevent further vessel damage. Here's a detailed look at the commonly used medications for SVV, their mechanisms of action, and potential side effects:<br><br>### 1. **Corticosteroids** (e.g., Prednisone)<br>- **Mechanism of Action**: Corticosteroids suppress the immune system broadly and reduce inflammation. They inhibit multiple inflammatory pathways, which helps reduce the formation of immune complexes and their deposition in blood vessels.<br>- **Side Effects**: Long-term use can lead to weight gain, increased risk of infections, high blood sugar, mood swings, and osteoporosis.<br><br>### 2. **Cyclophosphamide**<br>- **Mechanism of Action**: Cyclophosphamide is an alkylating agent that interferes with the multiplication of immune cells, thereby reducing the production of immune complexes that cause vasculitis.<br>- **Side Effects**: Increased risk of infections, bone marrow suppression, hair loss, bladder toxicity, and an increased risk of certain cancers.<br><br>### 3. **Mycophenolate Mofetil**<br>- **Mechanism of Action**: This medication inhibits the proliferation of B and T lymphocytes, reducing the production of antibodies and immune complexes.<br>- **Side Effects**: Common side effects include gastrointestinal disturbances, increased risk of infections, and potential liver enzyme abnormalities.<br><br>### 4. **Azathioprine**<br>- **Mechanism of Action**: Azathioprine inhibits purine synthesis, which is necessary for the proliferation of cells, particularly lymphocytes involved in the immune response.<br>- **Side Effects**: It can cause bone marrow suppression, liver toxicity, increased susceptibility to infections, and rarely, certain cancers.<br><br>### 5. **Rituximab**<br>- **Mechanism of Action**: Rituximab is a monoclonal antibody that targets CD20 on the	Immune Complex Small Vessel Vasculitis (SVV) is a condition where the small blood vessels become swollen because of immune cells and proteins gathering in them. This can cause damage to the vessels. The main goal of treatment is to calm down the immune system to stop this from happening. Here's how the medicines for SVV work and what side effects they might have, explained simply:<br><br>### 1. **Corticosteroids** (like Prednisone)<br>- **What They Do**: These medicines help reduce swelling all over the body by calming down the immune system. They stop the body from reacting too strongly, which is what causes the swelling in the blood vessels.<br>- **Side Effects**: They can cause weight gain, make it easier to get infections, increase blood sugar, affect your mood, and weaken bones if used for a long time.<br><br>### 2. **Cyclophosphamide**<br>- **What They Do**: This medicine helps by stopping immune cells from growing too fast, which reduces the number of harmful proteins that can cause vessel damage.<br>- **Side Effects**: It can make it easier to catch infections, cause hair loss, hurt the bladder, and increase the risk of some cancers.<br><br>### 3. **Mycophenolate Mofetil**<br>- **What They Do**: This drug slows down the growth of specific immune cells that help create harmful proteins, thus reducing swelling.<br>- **Side Effects**: It can upset your stomach, make it easier to get infections, and might affect your liver.<br><br>### 4. **Azathioprine**<br>- **What They Do**: This medicine stops certain building blocks in cells from working, which slows down the growth of immune cells that cause swelling.<br>- **Side Effects**: It can lower your body's ability to fight infections, affect your liver, and very rarely increase the risk of cancer.<br><br>### 5. **Rituximab**<br>- **What They Do**: This medicine targets specific cells in the immune system that make harmful proteins and removes them.

		surfaces of B-cells, leading to their depletion. This reduces the production of autoantibodies that form immune complexes.<br>- **Side Effects**: Potential side effects include infusion reactions, increased risk of infections, and, in rare cases, progressive multifocal leukoencephalopathy (a serious brain infection).<br><br>### 6. **Plasmapheresis**<br>- **Mechanism of Action**: Though not a medication, plasmapheresis is sometimes used in severe cases. It involves filtering the blood to remove harmful immune complexes and antibodies.<br>- **Side Effects**: Possible side effects include low blood pressure, bleeding due to anticoagulation during the procedure, and risk of infections.<br><br>### 7. **Biologics** (e.g., Tocilizumab, Belimumab)<br>- **Mechanism of Action**: These targeted therapies block specific parts of the immune system, like cytokines or specific cells, which are involved in the inflammatory process.<br>- **Side Effects**: They can cause infections, reactions at the injection site, and potential liver issues, among others.<br><br>### Supportive Care<br>- **Purpose**: Depending on the organs involved, treatments may also include medications to manage symptoms and prevent complications, such as antihypertensives for blood pressure control or immunoglobulin therapy.<br><br>Treatment plans for SVV are highly personalized and depend on the severity and extent of organ involvement. Continuous monitoring and adjustments are crucial due to the potential severe side effects of these potent medications. Regular follow-ups with healthcare providers are essential to manage the condition effectively and minimize the risks associated with therapy.	- **Side Effects**: It can cause reactions during the treatment, make you more likely to get infections, and in very rare cases, cause a serious brain infection.<br><br>### 6. **Plasmapheresis**<br>- **What It Does**: This is a procedure, not a medicine. It involves filtering the blood to remove harmful immune cells and proteins.<br>- **Side Effects**: It can cause low blood pressure, bleeding due to the blood thinners used during the procedure, and increase the risk of infections.<br><br>### 7. **Biologics** (like Tocilizumab, Belimumab)<br>- **What They Do**: These treatments specifically target parts of the immune system involved in causing inflammation and swelling.<br>- **Side Effects**: They can lead to infections, cause reactions where the medicine is injected, and affect the liver.<br><br>### Supportive Care<br>- **Purpose**: Depending on which parts of the body are affected, doctors might also give medicines to help with symptoms like high blood pressure or provide treatments that help strengthen the immune system.<br><br>Treatments for SVV need to be carefully chosen and watched by doctors to make sure they're working and not causing serious side effects. Regular check-ups are important to adjust the treatment as needed and keep the patient feeling as good as possible.
11	IgA Vasculitis (Henoch-Schönlein Purpura)	IgA Vasculitis, also known as Henoch-Schönlein Purpura (HSP), is an inflammation of small blood vessels caused by the deposition of Immunoglobulin A (IgA) antibodies. This condition often affects the skin, gastrointestinal tract, joints, and kidneys. Treatment focuses on managing symptoms and preventing complications, especially if the kidneys are involved. Here’s an overview of the medications commonly used, how they work, and their potential side effects:<br><br>### 1. **Corticosteroids** (e.g., Prednisone)<br>- **Mechanism of Action**: Corticosteroids are used to reduce inflammation by suppressing the immune system. They help decrease the immune response that causes inflammation and blood vessel damage in HSP.<br>- **Side Effects**: Potential side effects include increased appetite, weight gain, mood swings, high blood pressure, high blood sugar, and with long-term use, bone weakening, and increased risk of infections.<br><br>### 2. **Nonsteroidal Anti-Inflammatory Drugs (NSAIDs)** (e.g., Ibuprofen, Naproxen)<br>- **Mechanism of Action**: NSAIDs are often used to manage pain and joint inflammation in HSP. They work by blocking enzymes in the body (COX-1 and COX-2) that produce chemicals called prostaglandins, which contribute to inflammation and pain.<br>- **Side Effects**: Common side effects include stomach upset, gastrointestinal bleeding, and kidney problems, especially with prolonged use.<br><br>### 3. **Immunosuppressants** (e.g., Azathioprine, Mycophenolate mofetil)<br>- **Mechanism of Action**: In more severe cases, especially those involving significant kidney	IgA Vasculitis, also known as Henoch-Schönlein Purpura (HSP), is a condition where some blood vessels get inflamed because of a type of antibody called IgA. This inflammation can affect the skin, stomach, joints, and sometimes kidneys. Here’s how the medicines used to treat HSP work and what side effects they might have, explained simply:<br><br>### 1. **Corticosteroids** (like Prednisone)<br>- **What They Do**: These medicines help calm down the body’s defense system and reduce swelling in the blood vessels.<br>- **Side Effects**: They can cause weight gain, mood changes, higher blood pressure, and higher blood sugar. If used for a long time, they might also weaken bones and make it easier to catch infections.<br><br>### 2. **Pain Relievers (NSAIDs)** (like Ibuprofen, Naproxen)<br>- **What They Do**: These are used to help with pain and swelling, especially in the joints.<br>- **Side Effects**: They can upset your stomach and might cause problems in your stomach like bleeding, especially if you use them a lot.<br><br>### 3. **Immunosuppressants** (like Azathioprine, Mycophenolate mofetil)<br>- **What They Do**: In serious cases, these medicines help by calming the immune system even more to reduce swelling and damage in the kidneys.<br>- **Side Effects**: These drugs can make it easier to catch infections and can affect the liver or kidneys. They might also decrease the number of blood cells, which

		involvement, immunosuppressants may be used to further suppress the immune system to reduce inflammation.<br>- **Side Effects**: These medications can increase the risk of infections, cause liver or kidney toxicity, and potentially lead to a decrease in blood cell counts.<br><br>### 4. **Colchicine**<br>- **Mechanism of Action**: Used primarily to treat joint symptoms in HSP, colchicine reduces inflammation by decreasing the activity of neutrophils, a type of white blood cell that contributes to inflammation.<br>- **Side Effects**: Side effects can include nausea, vomiting, stomach pain, and diarrhea. Rarely, it can cause low blood counts and muscle weakness.<br><br>### 5. **Angiotensin-Converting Enzyme (ACE) Inhibitors** (e.g., Enalapril, Lisinopril)<br>- **Mechanism of Action**: For HSP patients with significant kidney involvement, ACE inhibitors can help reduce proteinuria (protein in urine) and manage blood pressure, both of which can help protect kidney function.<br>- **Side Effects**: Possible side effects include a dry cough, high potassium levels, low blood pressure, and in rare cases, kidney function impairment.<br><br>### 6. **Rituximab**<br>- **Mechanism of Action**: In cases where traditional treatments are not effective, Rituximab, a monoclonal antibody that targets CD20 on B cells, may be used to reduce the levels of IgA-producing cells.<br>- **Side Effects**: Infusion reactions, increased risk of infections, and, very rarely, progressive multifocal leukoencephalopathy (a serious brain infection) can occur.<br><br>### Supportive Care<br>- **Purpose**: Treatment may also involve managing symptoms such as nausea, abdominal pain, and ensuring adequate hydration and nutrition, particularly if the gastrointestinal tract is affected.<br><br>Treatment strategies for IgA Vasculitis are personalized based on the severity of symptoms and organ involvement. Regular monitoring by healthcare providers is crucial to adjust the treatment plan as needed and to minimize potential side effects from these medications.	helps fight off infections.<br><br>### 4. **Colchicine**<br>- **What They Do**: This medicine is used mainly for joint pain in HSP. It works by reducing the activity of certain white blood cells that cause swelling.<br>- **Side Effects**: It can cause stomach pain, nausea, vomiting, and diarrhea. Very rarely, it might cause muscle weakness or lower your blood cell counts.<br><br>### 5. **Blood Pressure Medicine (ACE Inhibitors)** (like Enalapril, Lisinopril)<br>- **What They Do**: If the kidneys are affected, these medicines help reduce protein loss in urine and help control blood pressure, which protects the kidneys.<br>- **Side Effects**: They might cause a dry cough, raise potassium levels in the blood, lower blood pressure too much, and in rare cases, affect how well the kidneys work.<br><br>### 6. **Rituximab**<br>- **What They Do**: This is used when other treatments don't work well. It targets certain immune cells to reduce the number of harmful IgA antibodies.<br>- **Side Effects**: It can cause reactions during the treatment, increase the risk of infections, and very rarely, lead to a serious brain infection.<br><br>### Supportive Care<br>- **Purpose**: Doctors also take care of any stomach pain, nausea, and make sure you're drinking enough fluids, especially if your stomach is affected.<br><br>Treatments for IgA Vasculitis depend on how serious the symptoms are and which parts of the body are affected. Doctors keep a close eye on how well treatments are working and make changes if needed. Regular check-ups are important to help manage the condition and avoid side effects from the medicines.
12	Anti-GBM Disease	Anti-Glomerular Basement Membrane Disease (Anti-GBM Disease), also known as Goodpasture's Syndrome, is a rare autoimmune disorder in which the immune system attacks the glomerular basement membrane in the kidneys and often the lungs. This attack leads to inflammation and can cause significant damage. The treatment primarily aims to stop the immune attack and reduce inflammation. Here's an overview of commonly used medications, their mechanisms, and potential side effects:<br><br>### 1. **Corticosteroids** (e.g., Prednisone)<br>- **Mechanism of Action**: Corticosteroids reduce inflammation by suppressing the immune system broadly. They inhibit various inflammatory pathways, which helps reduce the immune system's attack on the kidneys and lungs.<br>- **Side Effects**: Long-term use can lead to weight gain, increased risk of infections, high blood sugar, mood swings, osteoporosis, and an increased risk of cataracts.<br><br>### 2. **Cyclophosphamide**<br>- **Mechanism of Action**: Cyclophosphamide is a cytotoxic drug that suppresses the immune system by interfering with the growth and function of immune cells, particularly those that produce the harmful antibodies in Anti-GBM Disease.	Anti-Glomerular Basement Membrane Disease (Anti-GBM Disease), also known as Goodpasture's Syndrome, is a rare illness where the body's defense system mistakenly attacks parts of the kidneys and sometimes the lungs. This can cause a lot of damage and needs serious treatment to stop the immune system from causing harm. Here's a simple explanation of the medications used and their side effects:<br><br>### 1. **Corticosteroids** (like Prednisone)<br>- **What They Do**: These medicines help reduce swelling and stop the immune system from attacking the body. They work by calming down the parts of the immune system that are too active.<br>- **Side Effects**: They can cause weight gain, make it easier to get infections, increase blood sugar levels, change moods, weaken bones, and can affect eyesight with long-term use.<br><br>### 2. **Cyclophosphamide**<br>- **What They Do**: This is a strong medicine that helps by slowing down the immune system, especially the cells that make harmful antibodies which attack the kidneys and lungs.

		- **Side Effects**: It can cause bone marrow suppression, increased risk of infections, hair loss, and potential bladder toxicity. There's also a long-term risk of secondary cancers.<br><br>### 3. **Plasmapheresis**<br>- **Mechanism of Action**: Although not a medication, plasmapheresis is a procedure often used in the treatment of Anti-GBM Disease. It involves filtering the blood to remove harmful antibodies that are attacking the kidneys and lungs.<br>- **Side Effects**: Potential side effects include low blood pressure, bleeding due to anticoagulation used during the procedure, and risk of infections.<br><br>### 4. **Rituximab**<br>- **Mechanism of Action**: Rituximab is a monoclonal antibody that targets CD20 on B-cells, leading to their depletion. By reducing B-cell numbers, it decreases the production of autoantibodies that contribute to the disease.<br>- **Side Effects**: Rituximab can cause infusion reactions, increased risk of infections, and, very rarely, progressive multifocal leukoencephalopathy (a serious brain infection).<br><br>### 5. **Mycophenolate Mofetil**<br>- **Mechanism of Action**: This immunosuppressive agent inhibits the proliferation of T and B lymphocytes, which are involved in the autoimmune response, thereby reducing the production of antibodies.<br>- **Side Effects**: Common side effects include gastrointestinal symptoms, increased risk of infections, and possible liver enzyme abnormalities.<br><br>### Additional Supportive Care:<br>- **Purpose**: Patients may also need supportive treatments depending on the severity of kidney or lung involvement, such as blood pressure medications, dialysis in cases of severe kidney failure, or supplemental oxygen for lung involvement.<br><br>The treatment approach for Anti-GBM Disease is aggressive and aims to quickly suppress the immune system to prevent irreversible damage to the kidneys and lungs. Regular monitoring and follow-up with healthcare providers are critical to manage the condition effectively and adjust treatments to minimize side effects and complications.	- **Side Effects**: It can make you more likely to get infections, cause hair loss, and might hurt the bladder. There's also a chance it could lead to other types of sickness like cancer after many years.<br><br>### 3. **Plasmapheresis**<br>- **What It Does**: This isn't a medicine but a treatment where blood is filtered to remove bad antibodies that are attacking the kidneys and lungs.<br>- **Side Effects**: It can cause low blood pressure, bleeding because of the blood thinners used during the process, and increase the risk of infections.<br><br>### 4. **Rituximab**<br>- **What It Does**: This medicine targets and removes specific immune cells (B-cells) that are part of the problem. By doing this, it helps reduce the production of the harmful antibodies.<br>- **Side Effects**: It can cause reactions during the treatment, make it easier to get infections, and in very rare cases, lead to a serious brain infection.<br><br>### 5. **Mycophenolate Mofetil**<br>- **What It Does**: This medicine also helps slow down the immune system by affecting specific immune cells, reducing their ability to cause damage.<br>- **Side Effects**: It might cause stomach upset, make it easier to get infections, and can sometimes affect the liver.<br><br>### Additional Supportive Care:<br>- **Purpose**: Depending on how severe the disease is, especially if it affects the kidneys or lungs seriously, other treatments like medicine for blood pressure, treatments to help with kidney function, or extra oxygen might be needed.<br><br>Doctors use these treatments to quickly control the immune system and prevent it from causing more damage to the body. It's really important for patients with this disease to see their doctor regularly to make sure the treatments are working right and adjust them if needed to avoid side effects.
13	Cryoglobulinemic Vasculitis	Cryoglobulinemic Vasculitis is a condition where abnormal proteins called cryoglobulins, which can clump together in cold temperatures, cause inflammation and damage to blood vessels. This condition often involves the skin, joints, and kidneys, and it can be associated with infections like hepatitis C or autoimmune diseases. The treatment typically aims to reduce inflammation, manage symptoms, and treat any underlying conditions that might be causing the cryoglobulins to form. Here are the common medications used to treat Cryoglobulinemic Vasculitis, their mechanisms of action, and potential side effects:<br><br>### 1. **Corticosteroids** (e.g., Prednisone)<br>- **Mechanism of Action**: Corticosteroids help reduce inflammation by broadly suppressing the immune system and inhibiting multiple inflammatory pathways.<br>- **Side Effects**: Long-term use can lead to weight gain, increased risk of infections, high blood sugar, mood swings, osteoporosis, and an increased risk of cataracts.<br><br>### 2. **Rituximab**<br>- **Mechanism of Action**: Rituximab is a monoclonal antibody that targets CD20 on B-cells, leading to their depletion. This reduces the production of cryoglobulins since B-cells are responsible for their production.<br>- **Side Effects**: Rituximab can cause infusion reactions, increased risk of infections, and,	Cryoglobulinemic Vasculitis is a condition where unusual proteins in the blood, called cryoglobulins, clump together when it gets cold. These clumps can cause inflammation and damage to blood vessels. This often affects the skin, joints, and kidneys and can be linked to infections like hepatitis C or autoimmune diseases. The treatment focuses on reducing inflammation, treating symptoms, and handling any underlying causes. Here's how the common medicines for this condition work and what side effects they might have, explained simply:<br><br>### 1. **Corticosteroids** (like Prednisone)<br>- **What They Do**: These medicines help reduce swelling by calming down the immune system and stopping it from causing inflammation.<br>- **Side Effects**: They can cause weight gain, make it easier to get infections, increase blood sugar, affect your mood, weaken bones, and cause problems with your eyes if used for a long time.<br><br>### 2. **Rituximab**<br>- **What It Does**: This medicine targets certain cells of the immune system that make the cryoglobulins and removes them.<br>- **Side Effects**: It can cause reactions during the treatment, increase the chance

		very rarely, progressive multifocal leukoencephalopathy (a serious brain infection).<br><br>### 3. **Plasmapheresis**<br>- **Mechanism of Action**: Plasmapheresis is a procedure that involves filtering the blood to remove cryoglobulins and other harmful immune complexes. This can quickly reduce symptoms and inflammation caused by these proteins.<br>- **Side Effects**: Potential side effects include low blood pressure, bleeding due to anticoagulation during the procedure, and the risk of infections.<br><br>### 4. **Antiviral Therapy** (for associated infections like Hepatitis C)<br>- **Mechanism of Action**: When Cryoglobulinemic Vasculitis is associated with viral infections like hepatitis C, antiviral medications such as sofosbuvir and ribavirin are used to treat the underlying infection, which can reduce the production of cryoglobulins.<br>- **Side Effects**: Side effects of antiviral therapy can include fatigue, headache, nausea, and in the case of ribavirin, hemolytic anemia.<br><br>### 5. **Immunosuppressants** (e.g., Cyclophosphamide, Azathioprine)<br>- **Mechanism of Action**: These drugs suppress the immune system more specifically, reducing the production of abnormal immune proteins including cryoglobulins.<br>- **Side Effects**: Increased susceptibility to infections, potential for liver and kidney toxicity, bone marrow suppression, and an increased risk of certain cancers.<br><br>### 6. **Cytokine Blockers** (e.g., Infliximab)<br>- **Mechanism of Action**: These biologic agents block tumor necrosis factor (TNF) and other cytokines involved in inflammation, reducing vascular inflammation caused by cryoglobulin deposits.<br>- **Side Effects**: They can cause increased risk of infections, potential liver issues, and, in rare cases, lymphoma or other cancers.<br><br>Treatment for Cryoglobulinemic Vasculitis is often tailored based on the underlying cause, the severity of the symptoms, and the specific organs involved. Regular monitoring by healthcare providers is crucial to manage the condition effectively, adjust treatments as needed, and minimize the potential side effects associated with these powerful medications.	of getting infections, and very rarely lead to a serious brain infection.<br><br>### 3. **Plasmapheresis**<br>- **What It Does**: This is a treatment where blood is filtered to remove the cryoglobulins and other harmful substances. It can quickly reduce symptoms and swelling.<br>- **Side Effects**: It can cause low blood pressure, bleeding because of the blood thinners used during the procedure, and increase the risk of infections.<br><br>### 4. **Antiviral Therapy** (for infections like Hepatitis C)<br>- **What It Does**: If the condition is caused by a virus like hepatitis C, antiviral medicines like sofosbuvir and ribavirin are used to treat the virus, which can help decrease the production of cryoglobulins.<br>- **Side Effects**: These medicines can make you feel tired, give you a headache, make you feel nauseous, and ribavirin can cause anemia.<br><br>### 5. **Immunosuppressants** (like Cyclophosphamide, Azathioprine)<br>- **What They Do**: These drugs slow down the immune system to stop it from making the abnormal proteins that cause trouble.<br>- **Side Effects**: They can make you more likely to get infections, hurt the liver and kidneys, decrease the number of blood cells that help you fight off infections, and increase the risk of some cancers.<br><br>### 6. **Cytokine Blockers** (like Infliximab)<br>- **What They Do**: These medicines block certain chemicals in the body that cause inflammation, helping to reduce swelling in the blood vessels.<br>- **Side Effects**: They can increase the risk of infections, cause liver issues, and in rare cases, lead to cancer.<br><br>Doctors tailor the treatment for Cryoglobulinemic Vasculitis based on what's causing it, how severe the symptoms are, and which parts of the body are affected. It's important to see the doctor regularly to make sure the treatments are working right and adjust them if needed to avoid side effects.
14	Hypocomplementemic Urticarial Vasculitis	Hypocomplementemic Urticarial Vasculitis (HUV) is a rare autoimmune disorder characterized by low levels of complement proteins in the blood, recurrent urticaria (hives), and inflammation of small blood vessels. This condition often involves the skin, joints, and sometimes internal organs such as the kidneys and lungs. The treatment aims to manage symptoms and prevent further inflammation and damage. Here's an overview of commonly used medications for HUV, their mechanisms of action, and potential side effects:<br><br>### 1. **Corticosteroids** (e.g., Prednisone)<br>- **Mechanism of Action**: Corticosteroids are effective at reducing inflammation and suppressing the immune system broadly. They inhibit multiple inflammatory pathways, which helps to alleviate symptoms of vasculitis and prevent further immune-mediated damage.<br>- **Side Effects**: Long-term use can lead to weight gain, increased risk of infections, high blood sugar, mood swings, osteoporosis, and an increased risk of cataracts.<br><br>### 2. **Antihistamines** (e.g., Diphenhydramine, Cetirizine)<br>- **Mechanism of Action**: Antihistamines are used to manage the urticarial (hives) component of HUV by blocking histamine receptors, which play a key role in allergic reactions and inflammation associated with hives.<br>- **Side Effects**: Drowsiness (more common with first-generation antihistamines like	Hypocomplementemic Urticarial Vasculitis (HUV) is a rare condition where the immune system mistakenly attacks parts of the body, causing inflammation in small blood vessels. This can lead to symptoms like hives on the skin, joint pain, and sometimes problems with internal organs like the kidneys. Here's how the treatments work and what side effects they might have, explained in a simple way:<br><br>### 1. **Corticosteroids** (like Prednisone)<br>- **What They Do**: These medicines help reduce swelling and calm down the immune system. They are good at stopping the inflammation that causes symptoms like pain and redness.<br>- **Side Effects**: If used for a long time, they can make you gain weight, increase your chance of getting infections, raise your blood sugar, affect your mood, weaken your bones, and cause problems with your eyes.<br><br>### 2. **Antihistamines** (like Diphenhydramine, Cetirizine)<br>- **What They Do**: These medicines help control hives and itching by blocking the effects of histamine, a chemical in the body that can cause allergy symptoms.<br>- **Side Effects**: They can make you sleepy (especially the older types like diphenhydramine), give you a dry mouth, and sometimes make you feel dizzy.

		diphenhydramine), dry mouth, and in some cases, dizziness.<br><br>### 3. **Immunosuppressants** (e.g., Azathioprine, Mycophenolate Mofetil)<br>- **Mechanism of Action**: These drugs suppress the immune system to reduce the production of autoantibodies and immune complexes that contribute to inflammation and tissue damage in HUV.<br>- **Side Effects**: Increased susceptibility to infections, potential for liver and kidney toxicity, and a risk of decreased blood cell counts leading to anemia or increased bleeding risk.<br><br>### 4. **Hydroxychloroquine**<br>- **Mechanism of Action**: Originally used as an antimalarial, hydroxychloroquine is effective in treating autoimmune diseases due to its immunomodulatory effects, reducing inflammation and helping to control immune responses.<br>- **Side Effects**: Stomach upset, skin rash, and very rarely, eye toxicity, which necessitates regular eye examinations when using this medication long-term.<br><br>### 5. **Colchicine**<br>- **Mechanism of Action**: Colchicine is used primarily for treating inflammatory symptoms in HUV, particularly for joint pain and swelling. It works by reducing the activity of neutrophils, a type of white blood cell involved in inflammation.<br>- **Side Effects**: Gastrointestinal issues such as diarrhea and nausea are common. Long-term use can lead to bone marrow suppression and muscle weakness.<br><br>### 6. **Biologic Agents** (e.g., Rituximab)<br>- **Mechanism of Action**: Rituximab, a monoclonal antibody that targets CD20 on B cells, is used in more severe cases. It helps reduce the number of B cells, lowering the production of harmful antibodies that contribute to inflammation.<br>- **Side Effects**: Infusion reactions, increased risk of infections, and, very rarely, progressive multifocal leukoencephalopathy (a serious brain infection).<br><br>### Supportive Care<br>- **Purpose**: Treatment may also include supportive measures such as pain management for joint symptoms and medications to address any specific organ involvement.<br><br>Managing Hypocomplementemic Urticarial Vasculitis often requires a combination of these medications, tailored to the severity of the symptoms and the specific manifestations of the disease. Regular monitoring by healthcare providers is crucial to adjust treatment as needed and to minimize potential side effects.	### 3. **Immunosuppressants** (like Azathioprine, Mycophenolate Mofetil)<br>- **What They Do**: These drugs weaken the immune system to stop it from attacking the body, which helps reduce inflammation and damage to blood vessels.<br>- **Side Effects**: They can make it easier for you to catch infections, and might affect your liver and kidneys. They can also decrease the number of blood cells that help your body fight infections and stop bleeding.<br><br>### 4. **Hydroxychloroquine**<br>- **What They Do**: This medicine helps control the immune system and reduce inflammation. It's used in treating several autoimmune diseases.<br>- **Side Effects**: It can upset your stomach, cause skin rashes, and very rarely affect your eyes, so you need regular eye checks if you take it for a long time.<br><br>### 5. **Colchicine**<br>- **What They Do**: Colchicine is used mainly to reduce joint pain and swelling. It works by lessening the activity of certain white blood cells that cause inflammation.<br>- **Side Effects**: It can cause stomach upset, diarrhea, and nausea. If you use it for a long time, it might lead to more serious issues like weakening your muscles.<br><br>### 6. **Biologic Agents** (like Rituximab)<br>- **What They Do**: This type of medicine targets specific cells in the immune system that make harmful antibodies, reducing inflammation and damage.<br>- **Side Effects**: It can cause reactions during the treatment, make it easier to get infections, and in very rare cases, lead to a serious brain infection.<br><br>### Supportive Care<br>- **Purpose**: Doctors also use medicines to help with pain and manage other specific symptoms, depending on what parts of the body are affected.<br><br>Treating HUV often involves a combination of these medicines, and the choice depends on how serious the symptoms are. Regular visits to the doctor are important to make sure the medicines are working right and to change them if needed to avoid side effects.
15	Thin Basement Membrane Disease	Thin Basement Membrane Disease (TBMD), also known as benign familial hematuria, primarily involves the thinning of the glomerular basement membranes in the kidneys. Typically, this condition is characterized by persistent microscopic hematuria (blood in the urine) and is usually asymptomatic with a good prognosis. The treatment strategy for TBMD generally focuses on managing symptoms and monitoring renal function rather than treating the disease itself, as it is often benign and stable.<br><br>### Common Medications and Their Mechanisms of Action:<br><br>1. **Angiotensin-Converting Enzyme (ACE) Inhibitors**:<br>- **Examples**: Lisinopril, Enalapril<br>- **Mechanism**: These drugs help relax blood vessels by blocking the formation of a natural chemical that narrows blood vessels. This can be particularly helpful in reducing proteinuria (protein in the urine) which sometimes occurs in TBMD patients.	Thin Basement Membrane Disease (TBMD) is a kidney condition where the inner layers of the kidneys are thinner than usual. People with TBMD often have blood in their urine but might not feel sick. The goal of treatment is to keep an eye on kidney health and manage any symptoms, not to cure the disease, since it usually doesn't get worse.<br><br>### Common Medications and What They Do:<br><br>1. **ACE Inhibitors**:<br>- **Examples**: Lisinopril, Enalapril<br>- **What They Do**: These medicines help open up your blood vessels, making it easier for your heart to pump blood. This can help if there is protein in your urine, which can happen with TBMD.<br>- **Side Effects**: You might get a cough, feel dizzy, or have high potassium levels

		- **Potential Side Effects**: Cough, elevated blood potassium levels, low blood pressure, and kidney function deterioration.<br><br>2. **Angiotensin II Receptor Blockers (ARBs)**:<br>- **Examples**: Losartan, Valsartan<br>- **Mechanism**: Similar to ACE inhibitors, ARBs work by blocking the action of angiotensin II, thereby preventing the narrowing of blood vessels. This helps to reduce blood pressure and proteinuria.<br>- **Potential Side Effects**: Dizziness, hyperkalemia (high potassium levels), and potential renal impairment.<br><br>### Supportive Treatments:<br><br>- **Antihypertensive Therapy**: If hypertension is present, managing high blood pressure is crucial to prevent further renal damage.<br>- **Dietary Modifications**: Reducing protein intake may be suggested to decrease the burden on the kidneys.<br><br>### Monitoring and Lifestyle Adjustments:<br><br>Regular monitoring of kidney function through blood tests and urine analysis is recommended to detect any progression of renal impairment early. Patients are also advised to maintain a healthy lifestyle, avoid nonsteroidal anti-inflammatory drugs (NSAIDs), and manage other comorbid conditions such as diabetes or hypertension effectively.<br><br>### Conclusion:<br><br>The primary aim in managing TBMD is to monitor kidney function and manage symptoms like hypertension or proteinuria if they arise. The medications used do not treat TBMD directly but help in managing its potential complications and symptoms effectively. It's important for patients to have regular follow-ups with their healthcare provider to monitor their renal function and adjust treatments as needed. Always consult a nephrologist or your healthcare provider for personalized advice and management strategies tailored to your specific condition.	which can make you feel tired or weak.<br><br>2. **ARBs (Angiotensin II Receptor Blockers)**:<br>- **Examples**: Losartan, Valsartan<br>- **What They Do**: These work like ACE inhibitors but are better for some people because they have fewer side effects. They help keep your blood vessels open.<br>- **Side Effects**: These might make you feel dizzy, have high potassium levels, or affect your kidneys.<br><br>### Other Things That Can Help:<br><br>- **Blood Pressure Medicines**: If you have high blood pressure, it's important to keep it under control to protect your kidneys.<br>- **Eating Right**: Eating less protein might help your kidneys not have to work as hard.<br><br>### Keeping an Eye on Your Health:<br><br>It’s important to go to the doctor regularly to check how your kidneys are doing. They will test your blood and urine to see if anything has changed. Staying active and healthy, and avoiding pain medicines like ibuprofen can also help keep your kidneys healthy.<br><br>### To Sum It Up:<br><br>Taking care of TBMD is mostly about watching for any changes in your health and treating symptoms like high blood pressure or too much protein in your urine. The medicines don’t cure TBMD but help deal with these symptoms. Make sure to talk to your doctor about the best plan for you and keep up with regular check-ups.
16	Pauci-immune GN	Pauci-immune glomerulonephritis (GN) is a type of kidney inflammation that often occurs in association with systemic vasculitis diseases like Granulomatosis with polyangiitis (GPA) and Microscopic polyangiitis (MPA). It is called "pauci-immune" because there are few or no immune deposits seen in the kidneys when viewed under a microscope.<br><br>### Common Medications Used for Pauci-immune GN:<br><br>1. **Corticosteroids (Prednisone, Methylprednisolone)**:<br>- **Mechanism**: These medications reduce inflammation by suppressing the immune system, which is helpful in treating diseases where the immune system attacks the body's own tissues, like pauci-immune GN.<br>- **Potential Side Effects**: Increased risk of infections, high blood sugar levels, increased appetite, weight gain, mood swings, and bone thinning.<br><br>2. **Cyclophosphamide**:<br>- **Mechanism**: Cyclophosphamide is a cytotoxic drug that kills rapidly dividing cells, including those involved in the inflammatory process. It is often used for severe cases to induce remission.	Pauci-immune glomerulonephritis (GN) is a type of kidney problem where the body’s immune system mistakenly attacks the kidneys, causing inflammation. It often happens with other diseases that cause inflammation in blood vessels.<br><br>### Common Medications Used for Pauci-immune GN:<br><br>1. **Corticosteroids (like Prednisone and Methylprednisolone)**:<br>- **What They Do**: These medicines help calm down your body’s immune system to stop it from attacking your kidneys. They reduce swelling and inflammation.<br>- **Possible Side Effects**: You might get more infections, feel hungrier, gain weight, feel moodier, and your bones could get weaker.<br><br>2. **Cyclophosphamide**:<br>- **What It Does**: This strong medicine kills cells in your body that grow quickly, which can include cells that cause inflammation in your kidneys.<br>- **Possible Side Effects**: It can hurt your bladder and increase the risk of bladder cancer, make you more likely to get infections, lower your blood counts, and

		- **Potential Side Effects**: Bladder toxicity (which can lead to bladder cancer), bone marrow suppression (leading to low blood counts), increased risk of infections, and potential infertility.<br><br>3. **Rituximab**:<br>- **Mechanism**: Rituximab is a monoclonal antibody that targets and destroys B cells, a type of white blood cell that plays a role in inflammation and autoantibody production in autoimmune diseases.<br>- **Potential Side Effects**: Infusion reactions (fever, chills, fatigue), increased risk of infections, and rarely, severe skin and brain infections.<br><br>4. **Mycophenolate Mofetil**:<br>- **Mechanism**: This medication inhibits the proliferation of B and T lymphocytes, immune cells that are active in the autoimmune response, thus reducing inflammation and tissue damage.<br>- **Potential Side Effects**: Diarrhea, nausea, increased risk of infections, and potential for rare but serious side effects like blood cell abnormalities.<br><br>### Additional Treatments:<br><br>- **Plasma Exchange (Plasmapheresis)**: This procedure can be used to remove antibodies from the blood that are thought to be involved in the disease process, used particularly in rapidly progressive cases.<br><br>### Monitoring and Management:<br><br>Patients receiving treatment for pauci-immune GN typically require close monitoring due to the potential severity of the side effects, especially when using powerful immunosuppressants like cyclophosphamide. This includes regular blood tests to monitor blood counts, kidney function, and potential toxic effects of the medications.<br><br>### Conclusion:<br><br>The treatment of pauci-immune GN aims to reduce kidney inflammation and prevent further damage by using medications that suppress or modulate the immune system. Each of these medications comes with potential side effects, often requiring a balance between managing the disease and minimizing adverse effects. Treatment strategies are tailored to the severity of the disease and the individual patient's overall health status. Regular follow-ups with a nephrologist are crucial to adjust treatment plans as needed and to monitor for complications.	possibly cause infertility.<br><br>3. **Rituximab**:<br>- **What It Does**: Rituximab targets specific cells of your immune system that are involved in the inflammation and helps to decrease their numbers.<br>- **Possible Side Effects**: You might have reactions like fever or tiredness when you get the medicine, and there's a higher chance of getting infections.<br><br>4. **Mycophenolate Mofetil**:<br>- **What It Does**: This medicine slows down the activity of certain immune cells, reducing inflammation and damage in the kidneys.<br>- **Possible Side Effects**: It can cause stomach upset, nausea, and increase the risk of getting infections. It can also cause changes in blood cells that need to be monitored.<br><br>### Extra Help:<br><br>- **Plasma Exchange (Plasmapheresis)**: This is a special procedure where doctors filter your blood to remove harmful antibodies. It's sometimes used if the kidney problem is getting worse quickly.<br><br>### Keeping Track of Your Health:<br><br>It's very important to go to the doctor often if you're being treated for pauci-immune GN because the medicines can have strong effects. Your doctor will check your blood regularly to see how your kidneys are doing and to make sure the medicines aren't causing other problems.<br><br>### To Sum It Up:<br><br>Treating pauci-immune GN involves using medicines to control your immune system and protect your kidneys from further damage. These medicines can have some serious side effects, so your doctor will watch your health closely. Make sure to talk to your doctor about how you're feeling and follow their advice on managing your condition.
17	ANCA-associated Vasculitis	ANCA-associated vasculitis (AAV) is a group of diseases where the body's immune system mistakenly attacks its own blood vessels, causing inflammation and damage. This can affect various organs, including the kidneys, lungs, and nerves. Treatment focuses on controlling the immune system to reduce inflammation and prevent further damage.<br><br>### Common Medications Used for ANCA-Associated Vasculitis:<br><br>1. **Corticosteroids (e.g., Prednisone, Methylprednisolone)**:<br>- **Mechanism**: These drugs suppress the immune system broadly, reducing inflammation throughout the body.<br>- **Potential Side Effects**: Weight gain, increased risk of infections, diabetes, osteoporosis, mood swings, and cataracts.	ANCA-associated vasculitis (AAV) is a disease where the body's immune system attacks its own blood vessels by mistake. This causes swelling and damage and can affect organs like the kidneys, lungs, and nerves. The goal of treatment is to calm down the immune system to stop it from causing more harm.<br><br>### Common Medications Used for ANCA-Associated Vasculitis:<br><br>1. **Corticosteroids (like Prednisone and Methylprednisolone)**:<br>- **What They Do**: These medicines work by calming down the entire immune system to reduce swelling and redness in the body.<br>- **Possible Side Effects**: You might gain weight, get more infections, have mood swings, or develop problems like diabetes or weak bones.

		2. **Cyclophosphamide**:<br>- **Mechanism**: Cyclophosphamide is a chemotherapy drug that kills rapidly dividing cells, including those that are part of the immune response causing vasculitis.<br>- **Potential Side Effects**: Increased risk of infections, bladder toxicity (which can lead to bladder cancer), bone marrow suppression (leading to low blood cell counts), and infertility.<br><br>3. **Rituximab**:<br>- **Mechanism**: Rituximab is a monoclonal antibody that targets CD20 on B cells, leading to their depletion. B cells are a part of the immune system and play a role in inflammation by producing antibodies, including ANCA.<br>- **Potential Side Effects**: Infusion reactions, increased risk of infections, and rare cases of progressive multifocal leukoencephalopathy (a serious brain infection).<br><br>4. **Mycophenolate Mofetil**:<br>- **Mechanism**: This drug inhibits an enzyme necessary for the growth and function of T and B cells, thereby suppressing the immune response.<br>- **Potential Side Effects**: Gastrointestinal disturbances, increased risk of infections, and possible blood cell abnormalities.<br><br>5. **Azathioprine**:<br>- **Mechanism**: Azathioprine interferes with the synthesis of DNA, RNA, and proteins necessary for the proliferation of immune cells, thus dampening the immune response.<br>- **Potential Side Effects**: Liver toxicity, increased risk of infections, and bone marrow suppression.<br><br>### Additional Treatments:<br><br>- **Plasma Exchange (Plasmapheresis)**: This is used especially in severe cases or when there are rapidly worsening symptoms. It involves removing blood plasma that contains harmful antibodies and replacing it with good plasma or a plasma substitute.<br><br>### Monitoring and Management:<br><br>Due to the potential severe side effects of these medications, patients with ANCA-associated vasculitis require close monitoring. This includes regular blood tests to check for effects on blood cells, liver, and kidneys, and monitoring for signs of infections or other complications.<br><br>### Conclusion:<br><br>The treatment for ANCA-associated vasculitis is geared toward controlling the autoimmune response with medications that suppress the immune system. This helps reduce inflammation and prevent damage to vital organs. Because these medications can have serious side effects, it is crucial to have regular follow-ups with healthcare providers to manage the disease effectively and adjust treatments as necessary.	2. **Cyclophosphamide**:<br>- **What It Does**: This is a strong medicine that stops certain cells in the immune system from growing, which helps to stop the swelling in your blood vessels.<br>- **Possible Side Effects**: You can get more infections, have a sore bladder, lose some hair, and it can make it hard for you to have children later.<br><br>3. **Rituximab**:<br>- **What It Does**: This medicine targets specific cells in your immune system that help create the inflammation and gets rid of them.<br>- **Possible Side Effects**: You might feel unwell during the infusion (like getting a fever or feeling tired), and it can increase the chance of getting serious infections.<br><br>4. **Mycophenolate Mofetil**:<br>- **What It Does**: This medicine slows down the growth of immune cells that cause inflammation, helping to protect your blood vessels.<br>- **Possible Side Effects**: It can upset your stomach, make you more likely to get sick from infections, and affect your blood in ways that might make you feel tired or bruise easily.<br><br>5. **Azathioprine**:<br>- **What It Does**: Azathioprine stops the immune cells from growing too fast, which helps calm down the swelling in your vessels.<br>- **Possible Side Effects**: It can cause liver problems, make you more likely to get infections, and affect your blood cells.<br><br>### Extra Help:<br><br>- **Plasma Exchange (Plasmapheresis)**: In severe cases, doctors might clean your blood. They take out the part of the blood that has bad antibodies (which are causing the disease) and replace it with good fluid.<br><br>### Keeping Track of Your Health:<br><br>These medicines can have strong effects, so it’s very important to see your doctor regularly. They will do tests to make sure your blood, liver, and kidneys are okay and check that the medicine isn’t causing other problems.<br><br>### To Sum It Up:<br><br>Treating ANCA-associated vasculitis involves using medicines that make your immune system less active to stop it from damaging your blood vessels. These medicines need to be watched closely by a doctor because they can cause serious side effects. Make sure to follow your doctor’s advice and go to all your appointments to keep track of your health.
18	Microscopic Polyangiitis	Microscopic Polyangiitis (MPA) is a form of vasculitis that primarily affects small blood vessels. It can lead to severe damage in organs such as the kidneys, lungs, and nerves. The treatment typically involves medications that suppress the immune system to reduce inflammation and prevent further vessel and organ damage.<br><br>### Common Medications Used for Microscopic Polyangiitis:	Microscopic Polyangiitis (MPA) is a type of illness where the body’s immune system attacks small blood vessels, causing inflammation. This can hurt important organs like the kidneys and lungs. Doctors use special medicines to calm the immune system and stop it from causing more damage.<br><br>### Common Medications Used for Microscopic Polyangiitis:

		1. **Corticosteroids (such as Prednisone)**<br>- **Mechanism**: These medications suppress the immune system broadly and reduce inflammation throughout the body. They are often used as a first-line treatment to quickly control the symptoms of vasculitis.<br>- **Potential Side Effects**: Increased risk of infections, weight gain, high blood pressure, diabetes, osteoporosis, mood changes, and cataracts.<br><br>2. **Cyclophosphamide**<br>- **Mechanism**: This chemotherapy agent inhibits the growth of immune cells that are involved in the inflammatory process. It is used for inducing remission in severe cases of MPA.<br>- **Potential Side Effects**: Bladder toxicity, increased risk of infections, bone marrow suppression (leading to reduced blood cell counts), hair loss, and potential infertility.<br><br>3. **Rituximab**<br>- **Mechanism**: Rituximab is a monoclonal antibody that targets CD20-positive B cells, which are involved in the autoimmune process. It helps reduce the production of autoantibodies that are part of the disease mechanism in MPA.<br>- **Potential Side Effects**: Infusion reactions (like fever, chills, fatigue), increased risk of infections, and, rarely, severe skin and brain infections.<br><br>4. **Azathioprine**<br>- **Mechanism**: After remission is achieved, azathioprine helps maintain remission. It interferes with the synthesis of DNA and RNA, which are necessary for the proliferation of immune cells, thus reducing immune activity.<br>- **Potential Side Effects**: Increased risk of infections, potential liver damage, and bone marrow suppression.<br><br>5. **Mycophenolate Mofetil**<br>- **Mechanism**: This immunosuppressive agent also prevents the proliferation of B and T lymphocytes, key components in the immune response, thereby reducing inflammation.<br>- **Potential Side Effects**: Gastrointestinal issues, increased risk of infections, and potential blood cell abnormalities.<br><br>### Additional Treatments:<br><br>- **Plasma Exchange (Plasmapheresis)**: This may be used in severe cases, especially when there is significant kidney involvement. It involves removing blood plasma that contains harmful antibodies and replacing it with good plasma or a plasma substitute to quickly reduce inflammation.<br><br>### Monitoring and Management:<br><br>Patients being treated for MPA need careful and regular monitoring because of the severity of the disease and the potential side effects of the medications. Monitoring typically includes regular blood tests to check for effects on blood cells, liver and kidney functions, and for any signs of infections.<br><br>### Conclusion:<br><br>The treatment of MPA involves using powerful medications to control the autoimmune response and prevent further damage to blood vessels and organs. These treatments are potent and can carry significant side effects, so they must be managed carefully by healthcare	1. **Corticosteroids (like Prednisone)**<br>- **What They Do**: These medicines help cool down the inflammation by making the immune system less active. They're often used first to quickly control the disease.<br>- **Possible Side Effects**: You might get more infections, gain weight, have higher blood sugar, weak bones, mood changes, and possibly cataracts.<br><br>2. **Cyclophosphamide**<br>- **What It Does**: This strong medicine stops certain immune cells from growing, which helps to calm down the inflammation. It's used when the disease is very serious.<br>- **Possible Side Effects**: It can hurt your bladder, make you lose hair, lower your blood counts, increase your chance of infections, and it might affect having children later.<br><br>3. **Rituximab**<br>- **What It Does**: This medicine targets specific cells of your immune system that are causing trouble and gets rid of them. It helps stop the body from attacking itself.<br>- **Possible Side Effects**: You might feel unwell during the treatment (like getting a fever or feeling tired), and there's a higher risk of getting serious infections.<br><br>4. **Azathioprine**<br>- **What It Does**: After the disease is under control, this medicine helps keep it that way. It slows down the growth of immune cells.<br>- **Possible Side Effects**: It can increase your risk of infections, possibly cause liver problems, and affect your blood cells.<br><br>5. **Mycophenolate Mofetil**<br>- **What It Does**: This medicine also helps control the immune system by slowing down the growth of certain immune cells.<br>- **Possible Side Effects**: It might upset your stomach, make you more likely to get infections, and can affect your blood cells.<br><br>### Extra Help:<br><br>- **Plasma Exchange (Plasmapheresis)**: If the disease is really bad, especially if the kidneys are involved, doctors might clean your blood. They take out the bad stuff and put in good stuff to help calm the inflammation quickly.<br><br>### Keeping Track of Your Health:<br><br>If you are being treated for MPA, you need to see the doctor often. The doctor will do tests to check your blood, and how well your liver and kidneys are working, and watch for infections. This is because the medicines are very strong and can have serious side effects.<br><br>### To Sum It Up:<br><br>Treating MPA involves using strong medicines to control your immune system and protect your blood vessels and organs from damage. These medicines need careful

		providers with regular follow-up assessments to adjust the treatment as necessary and to manage any complications.	watching by a doctor, who will check on you regularly to make sure the treatment is working and to adjust it if needed.
19	Granulomatosis with Polyangiitis	Granulomatosis with Polyangiitis (GPA), formerly known as Wegener's granulomatosis, is a form of vasculitis that primarily affects the small to medium blood vessels in the body. It can lead to significant inflammation and damage to the respiratory tract, kidneys, and other organs. The treatment generally aims to suppress the immune system to reduce inflammation and prevent further organ damage.<br><br>### Common Medications Used for Granulomatosis with Polyangiitis:<br><br>1. **Corticosteroids (such as Prednisone)**<br>- **Mechanism**: These drugs suppress the immune system broadly to reduce inflammation throughout the body. They are often used as initial therapy to quickly control disease activity.<br>- **Potential Side Effects**: Increased risk of infections, weight gain, high blood pressure, diabetes, osteoporosis, mood swings, and cataracts.<br><br>2. **Cyclophosphamide**<br>- **Mechanism**: Cyclophosphamide is a chemotherapy drug that inhibits the growth of immune cells that are involved in the inflammatory process. It is used for inducing remission in severe cases of GPA.<br>- **Potential Side Effects**: Bladder toxicity, increased risk of infections, bone marrow suppression (leading to reduced blood cell counts), hair loss, and potential infertility.<br><br>3. **Rituximab**<br>- **Mechanism**: Rituximab is a monoclonal antibody that targets CD20-positive B cells, which are part of the immune system involved in the autoimmune process. It helps reduce the production of autoantibodies that are part of the disease mechanism in GPA.<br>- **Potential Side Effects**: Infusion reactions (such as fever, chills, fatigue), increased risk of infections, and, rarely, severe skin and brain infections.<br><br>4. **Methotrexate**<br>- **Mechanism**: Methotrexate is used as a maintenance therapy to prevent relapses of the disease. It interferes with DNA synthesis, inhibiting the proliferation of immune cells and reducing inflammation.<br>- **Potential Side Effects**: Liver toxicity, lung issues (such as pneumonitis), gastrointestinal upset, and potential for increased risk of infections.<br><br>5. **Azathioprine**<br>- **Mechanism**: Azathioprine is another option for maintenance therapy. It works by interfering with the synthesis of nucleic acids, necessary for the proliferation and survival of immune cells.<br>- **Potential Side Effects**: Increased risk of infections, potential liver damage, and bone marrow suppression.<br><br>### Additional Treatments:<br><br>- **Plasma Exchange (Plasmapheresis)**: This may be used in cases with severe organ involvement, such as rapidly progressive kidney disease. It involves removing blood plasma that contains harmful antibodies and replacing it with good plasma or a plasma substitute to quickly reduce inflammation.<br><br>### Monitoring and Management:	Granulomatosis with Polyangiitis (GPA), also known as Wegener's granulomatosis, is a disease where the body's immune system mistakenly attacks small and medium-sized blood vessels. This can cause problems in the lungs, kidneys, and other parts of the body. The goal of treatment is to calm down the immune system to stop it from causing damage.<br><br>### Common Medications Used for Granulomatosis with Polyangiitis:<br><br>1. **Corticosteroids (like Prednisone)**<br>- **What They Do**: These medicines help reduce swelling and inflammation by making the immune system less active. They are often used first to quickly control the disease.<br>- **Possible Side Effects**: You might get more infections, gain weight, have higher blood sugar, weak bones, mood changes, and possibly cataracts.<br><br>2. **Cyclophosphamide**<br>- **What It Does**: This strong medicine stops certain immune cells from growing, which helps to calm down the inflammation. It's used when the disease is very serious.<br>- **Possible Side Effects**: It can hurt your bladder, make you lose hair, lower your blood counts, increase your chance of infections, and it might affect having children later.<br><br>3. **Rituximab**<br>- **What It Does**: This medicine targets specific cells of your immune system that are causing trouble and gets rid of them. It helps stop the body from attacking itself.<br>- **Possible Side Effects**: You might feel unwell during the treatment (like getting a fever or feeling tired), and there's a higher risk of getting serious infections.<br><br>4. **Methotrexate**<br>- **What It Does**: This medicine is used to keep the disease from coming back. It slows down the growth of immune cells.<br>- **Possible Side Effects**: It can cause liver problems, lung problems like coughing and feeling short of breath, upset stomach, and increase your risk of infections.<br><br>5. **Azathioprine**<br>- **What It Does**: This medicine also helps keep the disease under control after it gets better. It works by slowing down the growth of immune cells.<br>- **Possible Side Effects**: It can increase your risk of infections, possibly cause liver problems, and affect your blood cells.<br><br>### Extra Help:<br><br>- **Plasma Exchange (Plasmapheresis)**: If the disease is really bad, especially if the kidneys are involved, doctors might clean your blood. They take out the bad stuff and put in good stuff to help calm the inflammation quickly.<br><br>### Keeping Track of Your Health:

		Patients with GPA require careful monitoring due to the aggressive nature of the disease and the potential severe side effects of the medications. Monitoring typically includes regular blood tests to check for effects on blood cells, liver and kidney functions, and for any signs of infections.<br><br>### Conclusion:<br><br>Treatment for GPA involves using powerful medications to control the immune system and prevent inflammation. These treatments are potent and can have significant side effects, so they must be managed carefully by healthcare providers with regular follow-up assessments to adjust the treatment as necessary and to manage any complications.	If you are being treated for GPA, you need to see the doctor often. The doctor will do tests to check your blood, and how well your liver and kidneys are working, and watch for infections. This is because the medicines are very strong and can have serious side effects.<br><br>### To Sum It Up:<br><br>Treating GPA involves using strong medicines to control your immune system and protect your blood vessels and organs from damage. These medicines need careful watching by a doctor, who will check on you regularly to make sure the treatment is working and to adjust it if needed.
20	Eosinophilic Granulomatosis with Polyangiitis	Eosinophilic Granulomatosis with Polyangiitis (EGPA), formerly known as Churg-Strauss syndrome, is a rare form of vasculitis that primarily affects small to medium-sized blood vessels and is associated with asthma and high levels of eosinophils, a type of white blood cell. The treatment focuses on controlling the inflammation and regulating the immune system's activity to prevent damage to organs such as the lungs and nerves.<br><br>### Common Medications Used for Eosinophilic Granulomatosis with Polyangiitis:<br><br>1. **Corticosteroids (e.g., Prednisone)**<br>- **Mechanism**: Corticosteroids are potent anti-inflammatory drugs that broadly suppress the immune system, reducing inflammation throughout the body.<br>- **Potential Side Effects**: Increased risk of infections, weight gain, high blood pressure, diabetes, osteoporosis, mood swings, and cataracts.<br><br>2. **Cyclophosphamide**<br>- **Mechanism**: Used in severe cases, cyclophosphamide is a chemotherapy agent that targets rapidly dividing cells, including those of the immune system, to reduce inflammation and autoimmune activity.<br>- **Potential Side Effects**: Bladder toxicity, increased risk of infections, bone marrow suppression (leading to low blood cell counts), hair loss, and potential infertility.<br><br>3. **Mepolizumab**<br>- **Mechanism**: Mepolizumab is a monoclonal antibody that targets interleukin-5 (IL-5), a key cytokine involved in the growth and activation of eosinophils. By blocking IL-5, mepolizumab reduces the number of eosinophils, thereby decreasing inflammation associated with EGPA.<br>- **Potential Side Effects**: Headache, injection site reactions, and a slightly increased risk of infections.<br><br>4. **Azathioprine**<br>- **Mechanism**: Azathioprine is used for maintenance therapy to prevent relapses of the disease. It interferes with the synthesis of DNA, RNA, and proteins necessary for the proliferation of immune cells, thereby suppressing immune activity.<br>- **Potential Side Effects**: Increased risk of infections, potential liver damage, and bone marrow suppression.<br><br>5. **Methotrexate**<br>- **Mechanism**: Methotrexate is another option for maintenance therapy that inhibits enzymes involved in the rapid growth of immune cells, reducing inflammation and the autoimmune response.	Eosinophilic Granulomatosis with Polyangiitis (EGPA), also known as Churg-Strauss syndrome, is a rare disease where the body’s immune system attacks its own blood vessels, especially in people with asthma. This attack causes swelling and can damage organs like the lungs and nerves. The treatment helps control this swelling by calming down the immune system.<br><br>### Common Medications Used for Eosinophilic Granulomatosis with Polyangiitis:<br><br>1. **Corticosteroids (like Prednisone)**<br>- **What They Do**: These medicines help reduce swelling by making the immune system less active. They're often used first to quickly control the disease.<br>- **Possible Side Effects**: You might get more infections, gain weight, have higher blood sugar, weak bones, mood changes, and possibly cataracts.<br><br>2. **Cyclophosphamide**<br>- **What It Does**: This strong medicine stops certain immune cells from growing, which helps to calm down the inflammation. It's used when the disease is very serious.<br>- **Possible Side Effects**: It can hurt your bladder, make you lose hair, lower your blood counts, increase your chance of infections, and it might affect having children later.<br><br>3. **Mepolizumab**<br>- **What It Does**: This medicine targets a specific part of the immune system involved in increasing eosinophils, which are cells that can cause a lot of the swelling in EGPA.<br>- **Possible Side Effects**: You might get headaches, have reactions where the medicine is injected, like redness or pain, and there's a slightly higher risk of getting sick from infections.<br><br>4. **Azathioprine**<br>- **What It Does**: This medicine helps keep the disease from coming back after it gets better. It slows down the growth of immune cells.<br>- **Possible Side Effects**: It can increase your risk of infections, possibly cause liver problems, and affect your blood cells.<br><br>5. **Methotrexate**<br>- **What It Does**: This medicine is used to keep the disease under control. It works by slowing down parts of the immune system that can cause swelling.<br>- **Possible Side Effects**: It can cause liver problems, lung problems like

		- **Potential Side Effects**: Liver toxicity, lung issues such as pneumonitis, gastrointestinal upset, and increased risk of infections.<br><br>### Additional Treatments:<br><br>- **Rituximab**: Sometimes used in cases where patients do not respond well to other treatments. It targets CD20-positive B cells, reducing their ability to contribute to the autoimmune process.<br>- **Intravenous Immunoglobulin (IVIG)**: This can be used in severe cases or for those with recurrent disease. IVIG provides the body with normal antibodies, which can help modulate the immune response.<br><br>### Monitoring and Management:<br><br>Patients receiving treatment for EGPA require regular monitoring to manage side effects and assess the effectiveness of the treatment. This includes routine blood tests to monitor blood cell counts, liver and kidney function, and tests to check for signs of infection.<br><br>### Conclusion:<br><br>Treating EGPA involves using medications that modify the immune system's response, aiming to reduce inflammation and prevent organ damage. Due to the potential severe side effects of these medications, close monitoring by healthcare providers is essential to adjust treatment as necessary and to manage any complications. The choice of treatment depends on the severity of the disease and the specific symptoms and organs involved.	coughing and feeling short of breath, upset stomach, and increase your risk of infections.<br><br>### Extra Help:<br><br>- **Rituximab**: Sometimes used if other treatments aren't working well. It specifically targets immune cells that are causing problems.<br>- **Intravenous Immunoglobulin (IVIG)**: This is given through a vein and can help calm the immune system in severe cases or when the disease comes back.<br><br>### Keeping Track of Your Health:<br><br>If you are being treated for EGPA, you need to visit the doctor often. They will check your blood to see how the medicines are affecting your body and make sure your liver and kidneys are working right. They will also watch for signs that you might be getting an infection.<br><br>### To Sum It Up:<br><br>Treating EGPA means using medicines that help control the immune system and reduce swelling to protect your blood vessels and organs from damage. These medicines are very strong and can have serious side effects, so doctors need to watch how you are doing very closely. They will check on you regularly to make sure the treatment is working and adjust it if needed.
21	MPGN	Membranoproliferative glomerulonephritis (MPGN) is a complex kidney disorder characterized by changes in the kidney structures called glomeruli. It involves the buildup of immune complexes in the kidneys, leading to inflammation and damage. Treatment for MPGN often targets the underlying cause of the immune complex deposition and aims to control symptoms and prevent progression of kidney disease.<br><br>### Common Medications Used for MPGN:<br><br>1. **Corticosteroids (such as Prednisone)**<br>- **Mechanism**: These drugs reduce inflammation by suppressing the immune system broadly, thus reducing the production of immune complexes that cause kidney damage.<br>- **Potential Side Effects**: Increased risk of infections, high blood sugar, weight gain, increased blood pressure, mood swings, bone thinning, and cataracts.<br><br>2. **Cyclophosphamide**<br>- **Mechanism**: Cyclophosphamide is a cytotoxic drug that kills rapidly dividing cells, including immune cells, thus reducing the formation of harmful immune complexes.<br>- **Potential Side Effects**: Bladder toxicity, increased risk of infections, bone marrow suppression (leading to reduced blood cell counts), hair loss, and potential infertility.<br><br>3. **Mycophenolate Mofetil**<br>- **Mechanism**: This immunosuppressant inhibits the proliferation of B and T lymphocytes, immune cells that are active in the autoimmune response, thus reducing inflammation and tissue damage.<br>- **Potential Side Effects**: Diarrhea, nausea, increased risk of infections, and potential for rare but serious side effects like blood cell abnormalities.	Membranoproliferative glomerulonephritis (MPGN) is a kidney disease where certain parts of the kidneys called glomeruli get damaged. This happens because of harmful immune complexes (a type of substance made when the body's immune system is overactive) that build up in the kidneys, causing inflammation and harm. The treatments focus on stopping the immune system from creating these harmful substances and helping to manage the symptoms to protect the kidneys.<br><br>### Common Medications Used for MPGN:<br><br>1. **Corticosteroids (like Prednisone)**<br>- **What They Do**: These medicines help reduce swelling and calm down the immune system to stop it from causing harm to the kidneys.<br>- **Possible Side Effects**: You might get more infections, higher blood sugar, gain weight, have higher blood pressure, mood changes, weak bones, and possibly cataracts.<br><br>2. **Cyclophosphamide**<br>- **What It Does**: This strong medicine helps stop certain immune cells from growing too fast, which can help decrease the harmful substances that damage the kidneys.<br>- **Possible Side Effects**: It can hurt your bladder, make you lose hair, lower your blood counts, increase your chance of infections, and it might affect having children later.<br><br>3. **Mycophenolate Mofetil**<br>- **What It Does**: This medicine slows down the growth of immune cells that cause inflammation, helping to protect your kidneys.

		4. **Rituximab**<br>- **Mechanism**: Rituximab is a monoclonal antibody that targets CD20 on B cells, leading to their depletion. B cells are a part of the immune system and play a role in inflammation by producing antibodies, including those that form immune complexes in MPGN.<br>- **Potential Side Effects**: Infusion reactions (fever, chills, fatigue), increased risk of infections, and rarely, severe skin and brain infections.<br><br>5. **Angiotensin-Converting Enzyme (ACE) Inhibitors (such as Lisinopril) or Angiotensin II Receptor Blockers (ARBs, such as Losartan)**<br>- **Mechanism**: These medications help control blood pressure and reduce proteinuria (protein in the urine), which can be a symptom of ongoing kidney damage. They work by relaxing blood vessels and reducing the workload on the kidneys.<br>- **Potential Side Effects**: Dizziness, increased blood potassium levels, and, in rare cases, kidney function deterioration.<br><br>### Additional Treatments:<br><br>- **Plasma Exchange (Plasmapheresis)**: This procedure may be used in severe cases to remove harmful immune complexes directly from the blood, thereby reducing their deposition in the kidneys.<br><br>### Monitoring and Management:<br><br>Patients being treated for MPGN need careful and regular monitoring due to the potential severity of the side effects and the progressive nature of the disease. This includes regular blood tests to monitor kidney function, blood counts, and potential toxic effects of the medications.<br><br>### Conclusion:<br><br>The treatment of MPGN involves medications that modulate the immune system's activity to reduce the formation of harmful immune complexes and control symptoms like high blood pressure and proteinuria. Due to the potential for significant side effects from these treatments, close monitoring by healthcare providers is essential to adjust treatments as necessary and manage any complications.	- **Possible Side Effects**: It might upset your stomach, make you more likely to get infections, and can affect your blood in ways that might make you feel tired or bruise easily.<br><br>4. **Rituximab**<br>- **What It Does**: This medicine specifically targets immune cells that are part of the problem, reducing their numbers and helping to calm down the immune reaction.<br>- **Possible Side Effects**: You might have reactions like fever or tiredness when getting the medicine, and there's a higher chance of getting infections.<br><br>5. **ACE Inhibitors (like Lisinopril) or ARBs (like Losartan)**<br>- **What They Do**: These medicines help control your blood pressure and reduce protein in your urine, which can help protect your kidneys from further damage.<br>- **Possible Side Effects**: You might feel dizzy, have higher potassium levels, and rarely, they can affect your kidney function.<br><br>### Extra Help:<br><br>- **Plasma Exchange (Plasmapheresis)**: If the disease is really bad, doctors might do a special treatment where they clean your blood to remove the harmful immune complexes.<br><br>### Keeping Track of Your Health:<br><br>If you are being treated for MPGN, you need to visit the doctor often. They will check your blood to see how the medicines are affecting your body and to make sure your kidneys are working right. They will also watch for signs that you might be getting an infection.<br><br>### To Sum It Up:<br><br>Treating MPGN means using medicines to control the immune system and reduce swelling to protect your kidneys from damage. These medicines are strong and can have serious side effects, so doctors need to watch how you are doing very closely. They will check on you regularly to make sure the treatment is working and adjust it if needed.
22	Alport Syndrome	Alport Syndrome is a genetic condition characterized by kidney disease, hearing loss, and eye abnormalities. It primarily affects the kidneys' filtering system, leading to progressive loss of kidney function. The treatment for Alport Syndrome focuses on slowing the progression of kidney disease and managing symptoms. There is no cure, but medications can help control the decline in kidney function.<br><br>### Common Medications Used for Alport Syndrome:<br><br>1. **Angiotensin-Converting Enzyme (ACE) Inhibitors (such as Lisinopril, Enalapril)**<br>- **Mechanism**: These medications help relax blood vessels, lowering blood pressure and reducing the amount of protein leaked into the urine, which is beneficial for protecting kidney function.<br>- **Potential Side Effects**: Dry cough, elevated blood potassium levels, low blood pressure, and, rarely, kidney function deterioration.	Alport Syndrome is a genetic condition that mainly affects the kidneys, but can also impact hearing and vision. This disease makes the kidneys' filtering system get worse over time, leading to kidney problems. While there's no cure, certain medicines can help slow down the damage to the kidneys.<br><br>### Common Medications Used for Alport Syndrome:<br><br>1. **ACE Inhibitors (like Lisinopril and Enalapril)**<br>- **What They Do**: These medicines help relax the blood vessels, lower blood pressure, and reduce protein in the urine. This is good because it helps protect the kidneys from getting worse.<br>- **Possible Side Effects**: You might get a dry cough, feel dizzy, or have high levels of potassium in your blood. Sometimes, they can also affect how well your kidneys work.

		2. **Angiotensin II Receptor Blockers (ARBs, such as Losartan, Valsartan)**<br>- **Mechanism**: Similar to ACE inhibitors, ARBs lower blood pressure and reduce proteinuria by blocking the action of angiotensin II, which causes blood vessels to tighten. This also helps in reducing stress on the kidneys.<br>- **Potential Side Effects**: Dizziness, high potassium levels, and occasionally, impacts on kidney function.<br><br>### Additional Supportive Treatments:<br><br>- **Statins**: If cholesterol levels are high, statins may be prescribed to help reduce the risk of cardiovascular disease.<br>- **Dietary Changes**: A low-salt, low-protein diet may be recommended to ease the workload on the kidneys.<br>- **Hearing Aids and Vision Correction**: As Alport Syndrome can affect hearing and vision, aids and corrective measures might be necessary.<br><br>### Monitoring and Management:<br><br>Patients with Alport Syndrome need regular monitoring of their kidney function through blood tests and urine tests to check protein levels. Regular check-ups can help adjust treatments as necessary and monitor for complications such as worsening of kidney function.<br><br>### Conclusion:<br><br>While there is no cure for Alport Syndrome, treatments like ACE inhibitors and ARBs are critical in managing the disease's progression by controlling blood pressure and reducing proteinuria. These medications help slow down the damage to the kidneys, extending kidney function over time. Given the potential side effects, regular follow-up with healthcare providers is essential for managing the condition effectively.	2. **ARBs (like Losartan and Valsartan)**<br>- **What They Do**: These work like ACE inhibitors but are a bit different in how they help. They also relax blood vessels and help lower blood pressure to protect the kidneys.<br>- **Possible Side Effects**: You might feel dizzy, have high potassium levels, and rarely, they can also affect your kidney function.<br><br>### Other Things That Can Help:<br><br>- **Statins**: If you have high cholesterol, your doctor might give you medicines called statins to help keep your heart healthy.<br>- **Eating Right**: Eating less salt and protein can help your kidneys not have to work as hard.<br>- **Hearing Aids and Glasses**: If you have trouble hearing or seeing because of Alport Syndrome, you might need hearing aids or glasses.<br><br>### Keeping Track of Your Health:<br><br>If you have Alport Syndrome, you need to go to the doctor often to check on your kidneys. They will do blood tests and urine tests to see how much protein is in your urine and how your kidneys are doing. Regular visits can help your doctor make sure your treatments are working right and change them if needed.<br><br>### To Sum It Up:<br><br>There's no cure for Alport Syndrome, but medicines like ACE inhibitors and ARBs are very important in managing the disease. They help slow down kidney damage by controlling blood pressure and reducing protein in the urine. Because these medicines can have side effects, it's important to see your doctor regularly to keep an eye on how you're doing and make sure the treatments are working well.
23	Lupus Nephritis	Lupus Nephritis is a serious condition where the immune system mistakenly attacks the kidneys, causing inflammation and damage. It is one of the most severe complications of systemic lupus erythematosus (SLE), an autoimmune disease. The treatment aims to reduce inflammation, suppress the immune response, and prevent further kidney damage.<br><br>### Common Medications Used for Lupus Nephritis:<br><br>1. **Corticosteroids (such as Prednisone)**<br>- **Mechanism**: These drugs broadly suppress the immune system and reduce inflammation throughout the body. They are often used to quickly control acute flares of lupus.<br>- **Potential Side Effects**: Weight gain, increased risk of infections, high blood pressure, mood swings, and long-term risks like diabetes, osteoporosis, and cataracts.<br><br>2. **Cyclophosphamide**<br>- **Mechanism**: Cyclophosphamide is a chemotherapy drug that suppresses the immune system by killing rapidly dividing cells, including those of the immune system that are causing inflammation.<br>- **Potential Side Effects**: Bladder toxicity, increased risk of infections, bone marrow suppression (leading to low blood cell counts), hair loss, and potential infertility.	Lupus Nephritis is when the immune system mistakenly attacks the kidneys in someone who has lupus, an autoimmune disease. This attack causes swelling and damage to the kidneys. The goal of the treatment is to calm down the immune system, reduce swelling, and protect the kidneys from more damage.<br><br>### Common Medications Used for Lupus Nephritis:<br><br>1. **Corticosteroids (like Prednisone)**<br>- **What They Do**: These medicines work by calming down the whole immune system to reduce swelling all over the body. They are used especially when lupus flares up suddenly.<br>- **Possible Side Effects**: You might gain weight, get infections more easily, have higher blood pressure, feel moody, and over time, you could get diabetes, weak bones, and eye problems.<br><br>2. **Cyclophosphamide**<br>- **What It Does**: This strong medicine helps by stopping immune cells that grow too fast and cause inflammation from growing. It's used for serious cases.<br>- **Possible Side Effects**: It can cause problems in your bladder, more infections, lower your blood counts, cause hair loss, and might affect having children later.

		3. **Mycophenolate Mofetil (MMF)**<br>- **Mechanism**: MMF inhibits the proliferation of B and T lymphocytes, key components of the immune response, thereby reducing inflammation and damage to the kidneys.<br>- **Potential Side Effects**: Gastrointestinal upset (like stomach pain and diarrhea), increased risk of infections, and possible blood cell abnormalities.<br><br>4. **Azathioprine**<br>- **Mechanism**: Azathioprine interferes with the synthesis of DNA, RNA, and proteins necessary for the proliferation of immune cells, thereby reducing immune activity.<br>- **Potential Side Effects**: Increased risk of infections, liver toxicity, and bone marrow suppression.<br><br>5. **Rituximab**<br>- **Mechanism**: Rituximab is a monoclonal antibody that targets CD20 on B cells, leading to their depletion. B cells are a part of the immune system and play a role in inflammation and autoantibody production in lupus.<br>- **Potential Side Effects**: Infusion reactions (fever, chills, fatigue), increased risk of infections, and rarely, severe skin and brain infections.<br><br>6. **Belimumab**<br>- **Mechanism**: Belimumab is a monoclonal antibody that inhibits a protein called B-lymphocyte stimulator (BLyS), which is important for the survival and proliferation of B cells. By blocking this protein, belimumab helps reduce the activity of the immune system in lupus.<br>- **Potential Side Effects**: Nausea, diarrhea, fever, and also a risk of infections.<br><br>### Additional Treatments:<br><br>- **Hydroxychloroquine**: Often used in lupus treatment, it helps reduce flares and is believed to offer protection against kidney damage.<br>- **ACE Inhibitors or ARBs**: These are used to manage hypertension and reduce proteinuria, which are common in lupus nephritis.<br><br>### Monitoring and Management:<br><br>Patients with lupus nephritis require careful monitoring to manage side effects and assess the effectiveness of the treatment. This includes regular blood tests to check for effects on blood cells, liver and kidney function, and monitoring for signs of infections.<br><br>### Conclusion:<br><br>The treatment of lupus nephritis involves a combination of medications aimed at controlling the autoimmune response and preventing kidney damage. Given the potential for significant side effects from these treatments, regular follow-up with healthcare providers is crucial to adjust treatments as necessary and to manage any complications.	3. **Mycophenolate Mofetil (MMF)**<br>- **What It Does**: This medicine slows down the growth of immune cells that cause inflammation, helping to protect your kidneys.<br>- **Possible Side Effects**: It might upset your stomach, cause diarrhea, increase the chance of getting infections, and can affect your blood in ways that might make you feel tired or bruise easily.<br><br>4. **Azathioprine**<br>- **What It Does**: This medicine works by stopping immune cells from growing too fast, which helps calm down the immune system.<br>- **Possible Side Effects**: It can increase your risk of infections, cause liver problems, and affect your blood cells.<br><br>5. **Rituximab**<br>- **What It Does**: This medicine targets specific immune cells that are part of the problem, reducing their numbers and helping to calm down the immune reaction.<br>- **Possible Side Effects**: You might have reactions like fever or tiredness when getting the medicine, and there’s a higher chance of getting infections.<br><br>6. **Belimumab**<br>- **What It Does**: This medicine stops a protein that helps immune cells grow, reducing the activity of the immune system in lupus.<br>- **Possible Side Effects**: You might feel nauseous, have diarrhea, get a fever, and there's also a risk of infections.<br><br>### Other Things That Can Help:<br><br>- **Hydroxychloroquine**: Often used to help reduce lupus flares and protect the kidneys.<br>- **ACE Inhibitors or ARBs**: These medicines help manage high blood pressure and reduce protein in the urine, which can help keep your kidneys healthier.<br><br>### Keeping Track of Your Health:<br><br>If you have lupus nephritis, you need to visit the doctor often. They will do blood tests and other checks to see how well your kidneys are working and how the medicines are affecting your body. Regular visits help your doctor make sure the treatments are working right and change them if needed.<br><br>### To Sum It Up:<br><br>Treating lupus nephritis means using medicines to control the immune system and reduce swelling to protect your kidneys. These medicines are strong and can have serious side effects, so doctors need to watch how you are doing very closely. They will check on you regularly to make sure the treatment is working and adjust it if needed.
24	Antiphospholipid Antibody Syndrome	Antiphospholipid Antibody Syndrome (APS) is a disorder where the immune system mistakenly creates antibodies against certain normal proteins in the blood. This can lead to blood clots forming in veins and arteries, which can cause various complications like stroke, blood clots in the lungs (pulmonary embolism), or pregnancy-related issues. The primary goal of treatment in APS is to prevent clot formation.	Antiphospholipid Antibody Syndrome (APS) is a health condition where the body mistakenly makes antibodies against normal proteins in the blood, leading to blood clots. These clots can cause serious problems like strokes or issues during pregnancy. The main goal of treatment for APS is to prevent these clots from forming.

		### Common Medications Used for Antiphospholipid Antibody Syndrome:<br><br>1. **Aspirin (Low-dose)**<br>- **Mechanism**: Aspirin helps thin the blood by inhibiting platelets, the cells in the blood that help with clotting. By reducing the platelets' ability to clump together, aspirin lowers the risk of blood clots forming.<br>- **Potential Side Effects**: Stomach upset, increased risk of gastrointestinal bleeding, and rare allergic reactions.<br><br>2. **Warfarin (Coumadin)**<br>- **Mechanism**: Warfarin works by blocking the action of vitamin K, which is essential for the formation of clotting factors in the liver. This action slows down the clotting process, helping to prevent the formation of harmful blood clots.<br>- **Potential Side Effects**: Increased risk of bleeding, which requires regular blood tests to monitor the drug's intensity (INR test) to ensure safe and effective dosing.<br><br>3. **Heparin (Unfractionated and Low Molecular Weight Heparin like Enoxaparin)**<br>- **Mechanism**: Heparin enhances the activity of antithrombin, a natural blood thinner that helps reduce clotting by inactivating several enzymes in the clotting system.<br>- **Potential Side Effects**: Bleeding, osteoporosis with long-term use, and in rare cases, heparin-induced thrombocytopenia (HIT), a serious condition where the platelet count falls drastically and paradoxically increases the risk of clotting.<br><br>4. **Direct Oral Anticoagulants (DOACs) such as Apixaban, Rivaroxaban**<br>- **Mechanism**: DOACs work by directly inhibiting specific proteins in the blood clotting process. For example, Rivaroxaban blocks Factor Xa, which is crucial for the development of a blood clot.<br>- **Potential Side Effects**: Bleeding risks, although typically less monitoring is required compared to warfarin. Their use in APS is somewhat controversial and usually considered when patients have difficulties with other anticoagulants.<br><br>### Additional Treatments:<br><br>- **Immunosuppressive Agents**: In cases where thrombosis recurs despite anticoagulation, or where there is catastrophic APS, drugs like corticosteroids, rituximab, or other immunosuppressants may be used to dampen the immune response.<br>- **Hydroxychloroquine**: Particularly for patients with systemic lupus erythematosus and APS, it is used for its mild blood-thinning effects and potential to improve lipid profiles and insulin sensitivity.<br><br>### Monitoring and Management:<br><br>Patients with APS require careful monitoring to balance the risk of clotting with the risk of bleeding, especially when on medications like warfarin. Regular blood tests (like INR for warfarin users) and assessments are essential to ensure effective and safe treatment.<br><br>### Conclusion:<br><br>Managing APS involves using medications to prevent blood clots by thinning the blood or modulating the immune system's behavior. Due to the serious risks associated with both clotting and potential treatment side effects (especially bleeding), close monitoring by	### Common Medications Used for Antiphospholipid Antibody Syndrome:<br><br>1. **Aspirin (Low-dose)**<br>- **What It Does**: Aspirin helps keep the blood from clotting too easily. It stops the tiny cells in the blood, called platelets, from sticking together.<br>- **Possible Side Effects**: It can upset your stomach, cause more bleeding, especially in the stomach, and sometimes cause allergic reactions.<br><br>2. **Warfarin (Coumadin)**<br>- **What It Does**: Warfarin slows down how fast your blood clots by affecting certain vitamins in the body that are needed for blood clotting.<br>- **Possible Side Effects**: The main risk is bleeding. If you take warfarin, you need regular blood tests to make sure it's working right and not causing too much thinning of the blood.<br><br>3. **Heparin (including Enoxaparin)**<br>- **What It Does**: Heparin also makes the blood clot more slowly. It works faster than warfarin and is often used in hospitals or given as an injection at home.<br>- **Possible Side Effects**: It can cause bleeding, weaken bones if used for a long time, and very rarely, a serious problem where it actually causes more clots instead of fewer.<br><br>4. **Direct Oral Anticoagulants (DOACs) like Apixaban, Rivaroxaban**<br>- **What It Does**: These medicines directly stop specific parts of the clotting process in the blood. They're easier to manage because they don't need as many blood tests as warfarin.<br>- **Possible Side Effects**: The main risk is still bleeding, similar to other blood thinners.<br><br>### Other Things That Might Help:<br><br>- **Immunosuppressive Agents**: If blood clots keep happening despite the blood thinners, or in very severe cases, doctors might use stronger medicines that calm down the immune system.<br>- **Hydroxychloroquine**: This is used especially for patients who also have lupus. It helps a little with clotting and can also improve cholesterol and sugar levels in the blood.<br><br>### Keeping Track of Your Health:<br><br>If you have APS, you need to visit the doctor often to make sure the medicines are working properly without causing bleeding. They'll do blood tests, especially if you're taking warfarin, to make sure your blood isn't too thin or too thick.<br><br>### To Sum It Up:<br><br>Treating APS involves using medicines to make sure your blood doesn't clot too easily. Because these medicines can also increase the risk of bleeding, it's really important to keep in touch with your doctor, follow directions closely, and go for regular check-ups. This helps make sure the treatment is safe and effective.

		healthcare providers is crucial. This ensures that the treatment is appropriately adjusted as needed and that any complications are promptly addressed.	
25	Drug-related GN	Drug-related glomerulonephritis (GN) is a form of kidney inflammation that occurs as a reaction to certain medications. This condition can be triggered by drugs that cause an immune response leading to inflammation and damage in the kidney tissues. Treatment usually involves stopping the offending drug and managing the symptoms and effects of the inflammation. In some cases, additional treatments may be needed to control the immune system and reduce inflammation.<br><br>### Common Triggers and Management Strategies:<br><br>1. **NSAIDs (Nonsteroidal Anti-inflammatory Drugs)**<br>- **Mechanism**: NSAIDs, like ibuprofen and naproxen, reduce pain and inflammation by inhibiting enzymes called COX-1 and COX-2, which are involved in the inflammatory process. They can occasionally cause kidney damage by reducing blood flow within the kidneys, leading to inflammation.<br>- **Potential Side Effects**: Kidney impairment, gastrointestinal bleeding, increased blood pressure, and fluid retention.<br><br>2. **Antibiotics (like Penicillin, Rifampin)**<br>- **Mechanism**: These medications can induce an immune response that mistakenly targets kidney structures, leading to inflammation.<br>- **Potential Side Effects**: Allergic reactions, rashes, and in rare cases, direct toxicity to kidney cells.<br><br>3. **Diuretics (like Furosemide)**<br>- **Mechanism**: Diuretics increase urine production to help reduce fluid retention but can affect kidney function and lead to inflammation in susceptible individuals.<br>- **Potential Side Effects**: Electrolyte imbalances, dehydration, and altered kidney function.<br><br>### Treatment Options for Drug-related GN:<br><br>1. **Corticosteroids**<br>- **Mechanism**: Corticosteroids such as prednisone are used to reduce immune system activity and decrease inflammation in the kidneys.<br>- **Potential Side Effects**: Weight gain, increased risk of infections, high blood sugar, osteoporosis, and mood changes.<br><br>2. **Stopping the Offending Drug**<br>- **Mechanism**: The primary treatment for drug-induced GN is to discontinue the use of the drug that caused the reaction.<br>- **Potential Side Effects**: Depending on the drug, stopping it abruptly may require management of the underlying condition with alternative medications.<br><br>3. **Supportive Care**<br>- **Mechanism**: Managing blood pressure with ACE inhibitors or ARBs, controlling proteinuria, and ensuring good fluid and electrolyte balance are critical.<br>- **Potential Side Effects**: Blood pressure medications like ACE inhibitors may cause cough, elevated blood potassium levels, and rarely, kidney function deterioration.<br><br>### Monitoring and Management:	Drug-related glomerulonephritis (GN) is when certain medicines cause inflammation and damage to the kidneys. This problem can happen because the medicines make the body's immune system react badly, affecting the kidneys. The main way to treat this is to stop taking the medicine that caused the problem and take care of the kidneys while they heal.<br><br>### Common Medicines That Can Cause This Problem:<br><br>1. **NSAIDs (like ibuprofen and naproxen)**<br>- **What They Do**: These are painkillers that also reduce swelling. They work by blocking certain chemicals in the body that cause pain and swelling.<br>- **Possible Side Effects**: They can make it harder for blood to flow through the kidneys, which might damage them. They can also cause stomach bleeding, higher blood pressure, and swelling because of extra fluid in the body.<br><br>2. **Antibiotics (like Penicillin, Rifampin)**<br>- **What They Do**: Antibiotics fight infections, but sometimes they can cause the immune system to react in ways that harm the kidneys.<br>- **Possible Side Effects**: Allergic reactions, skin rashes, and sometimes direct harm to kidney cells.<br><br>3. **Diuretics (like Furosemide)**<br>- **What They Do**: These medicines help the body get rid of extra water by making more urine. This can help when there's too much fluid in the body.<br>- **Possible Side Effects**: They can cause the body to lose too many important salts, lead to dehydration, and change how well the kidneys work.<br><br>### How to Treat Drug-related GN:<br><br>1. **Corticosteroids (like Prednisone)**<br>- **What They Do**: These are strong medicines that help calm down the immune system and reduce swelling in the kidneys.<br>- **Possible Side Effects**: They can cause weight gain, make it easier to get infections, raise blood sugar levels, weaken bones, and affect moods.<br><br>2. **Stopping the Offending Drug**<br>- **What It Does**: The most important step is to stop taking the medicine that caused the kidney problem.<br>- **Potential Side Effects**: There might be other issues that need to be managed with different medicines if you stop the one that caused the problem.<br><br>3. **Supportive Care**<br>- **What It Does**: This includes taking care of blood pressure, reducing protein loss in urine, and making sure the body has the right balance of fluids and salts.<br>- **Possible Side Effects**: Blood pressure medicines might cause a cough, high potassium levels, and sometimes affect kidney function.<br><br>### Keeping Track of Your Health:<br><br>If you have drug-related GN, you need to see the doctor often to make sure your

		Patients with drug-related GN need careful monitoring to assess the recovery of kidney function and manage any complications from the inflammation or treatments. Regular kidney function tests, urine analysis for protein, and monitoring for potential side effects of any new medications are essential.<br><br>### Conclusion:<br><br>Drug-related GN involves managing the effects of medications that have caused kidney inflammation. Treatment primarily involves stopping the drug responsible and may include the use of steroids to reduce inflammation and supportive care to manage symptoms and protect kidney function. Regular follow-ups with healthcare providers are crucial to adjust treatment as necessary and to monitor for complications or recovery.	kidneys are getting better. They will check your kidney function with blood tests and urine tests. They will also watch for side effects from any new medicines you might need.<br><br>### To Sum It Up:<br><br>Drug-related GN happens when medicines cause your immune system to harm your kidneys. The main treatment is to stop taking the harmful medicine and use other medicines to help calm the immune system and heal the kidneys. Regular check-ups with your doctor are important to make sure everything is going well and adjust treatments if needed.
26	Alport Syndrome Post-transplant Anti-GBM Disease	Alport Syndrome is a genetic disorder that affects the kidneys and can lead to progressive loss of kidney function, often necessitating a kidney transplant. In some cases, after a kidney transplant, patients with Alport Syndrome can develop anti-glomerular basement membrane (anti-GBM) disease. This condition occurs when the immune system attacks the newly transplanted kidney, mistaking it for foreign tissue and damaging the glomerular basement membrane, a crucial structure in the kidneys.<br><br>### Common Medications Used for Alport Syndrome Post-Transplant Anti-GBM Disease:<br><br>1. **Corticosteroids (such as Prednisone)**<br>- **Mechanism**: These drugs are potent anti-inflammatory agents that broadly suppress the immune system to reduce the autoimmune reaction attacking the kidney's glomerular basement membrane.<br>- **Potential Side Effects**: Increased risk of infections, weight gain, high blood pressure, diabetes, osteoporosis, mood swings, and cataracts.<br><br>2. **Cyclophosphamide**<br>- **Mechanism**: Cyclophosphamide is a cytotoxic drug that suppresses the immune system by killing rapidly dividing cells, including those that are part of the immune response causing the anti-GBM disease.<br>- **Potential Side Effects**: Increased risk of infections, bone marrow suppression (leading to low blood cell counts), hair loss, bladder toxicity, and potential infertility.<br><br>3. **Rituximab**<br>- **Mechanism**: Rituximab is a monoclonal antibody that specifically targets CD20 on B cells, leading to their depletion. B cells are part of the immune system and contribute to the production of antibodies, including those involved in anti-GBM disease.<br>- **Potential Side Effects**: Infusion reactions (such as fever, chills, and fatigue), increased risk of infections, and rarely, severe skin and brain infections.<br><br>4. **Plasma Exchange (Plasmapheresis)**<br>- **Mechanism**: This treatment involves removing the patient's blood plasma, which contains the harmful anti-GBM antibodies, and replacing it with donor plasma or a plasma substitute to quickly reduce antibody levels and minimize kidney damage.<br>- **Potential Side Effects**: Hypotension (low blood pressure), bleeding due to anticoagulant use during the procedure, and allergic reactions to replacement fluids.<br><br>### Additional Considerations:	Alport Syndrome is a genetic condition that mainly affects the kidneys and can lead to serious kidney damage. Sometimes, people with Alport Syndrome need a kidney transplant. After getting a new kidney, some patients might develop a problem called anti-GBM disease, where the body's immune system attacks the new kidney thinking it's something harmful.<br><br>### Common Medications Used for Alport Syndrome Post-Transplant Anti-GBM Disease:<br><br>1. **Corticosteroids (like Prednisone)**<br>- **What They Do**: These are strong medicines that help calm down the immune system to stop it from attacking the new kidney.<br>- **Possible Side Effects**: You might gain weight, get more infections, have higher blood pressure, get diabetes, weak bones, mood swings, and possibly cataracts.<br><br>2. **Cyclophosphamide**<br>- **What It Does**: This medicine helps by stopping certain cells in the immune system that are attacking the kidney. It works by killing cells that grow too fast, which are part of the immune response causing problems.<br>- **Possible Side Effects**: It can increase the chance of getting infections, lower your blood counts, cause hair loss, and bladder problems. It might also affect having children later.<br><br>3. **Rituximab**<br>- **What It Does**: This medicine specifically targets and gets rid of certain immune cells (called B cells) that help produce harmful antibodies attacking the kidney.<br>- **Possible Side Effects**: You might have reactions like fever or feeling tired during the treatment, and there's a higher chance of getting infections. Rarely, it can cause serious skin and brain infections.<br><br>4. **Plasma Exchange (Plasmapheresis)**<br>- **What It Does**: This treatment involves taking out the part of your blood that has bad antibodies (which are causing the disease) and replacing it with good plasma or a plasma substitute.<br>- **Possible Side Effects**: You might feel dizzy or light-headed, have a risk of bleeding because of the medicines used to prevent clotting during the procedure, and sometimes have allergic reactions to the fluids used.

		- **Immunosuppressive Therapy**: Post-transplant patients are typically on a regimen of immunosuppressants to prevent organ rejection, including drugs like tacrolimus, mycophenolate mofetil, and sirolimus. These are continued and sometimes adjusted in the context of treating post-transplant anti-GBM disease.<br><br>### Monitoring and Management:<br><br>Patients with Alport Syndrome post-transplant anti-GBM disease require intensive monitoring to manage the balance between suppressing the autoimmune response and maintaining sufficient immune function to prevent infections. Regular blood tests to monitor kidney function, antibody levels, and the effects of medications are crucial.<br><br>### Conclusion:<br><br>The treatment of Alport Syndrome post-transplant anti-GBM disease involves aggressive immunosuppression to prevent the immune system from attacking the transplanted kidney. The use of medications like corticosteroids, cyclophosphamide, and rituximab, combined with procedures like plasma exchange, are essential to control this condition. Due to the potent nature of these treatments, close monitoring by healthcare providers is critical to adjust treatment as necessary and to manage any side effects or complications.	### Other Important Care:<br><br>- **Immunosuppressive Therapy**: Because you have a transplanted kidney, you'll also be taking medicines that help prevent your body from rejecting the new kidney. These include medicines like tacrolimus and mycophenolate mofetil.<br><br>### Keeping Track of Your Health:<br><br>If you have Alport Syndrome and get a new kidney, you need to see the doctor often. They will check your kidney function with blood tests and make sure the new kidney is working right. They will also check to make sure your medicines aren't causing other problems and adjust them if needed.<br><br>### To Sum It Up:<br><br>Treating Alport Syndrome after a kidney transplant, especially if you develop anti-GBM disease, involves using strong medicines to control your immune system and protect your new kidney. These treatments need careful watching by doctors to make sure they work right and don't cause serious side effects. Regular check-ups are very important to keep you healthy and to make sure your treatment is on track.
27	C3 Glomerulopathy	C3 Glomerulopathy is a rare kidney disorder characterized by the abnormal accumulation of C3 protein deposits in the kidney's glomeruli, the small units within the kidney where blood filtration occurs. The condition stems from dysregulation in the complement system, a part of the immune system that helps the body fight infections and also plays a role in inflammation. Currently, there is no standard treatment for C3 Glomerulopathy, but several approaches aim to manage symptoms and slow disease progression, focusing mainly on regulating the complement system.<br><br>### Common Medications Used for C3 Glomerulopathy:<br><br>1. **Eculizumab**<br>- **Mechanism**: Eculizumab is a monoclonal antibody that inhibits the C5 protein in the complement cascade, which prevents the formation of the membrane attack complex (MAC), a group of proteins that typically cause cell lysis and inflammation.<br>- **Potential Side Effects**: Increased risk of meningococcal infections, headaches, nausea, and hypertension. Vaccination against meningococcus is recommended before starting treatment.<br><br>2. **Mycophenolate Mofetil (MMF)**<br>- **Mechanism**: While primarily used as an immunosuppressant to prevent organ transplant rejection, MMF is also used in C3 Glomerulopathy to reduce proteinuria (excess protein in the urine) and manage the immune response indirectly.<br>- **Potential Side Effects**: Diarrhea, nausea, increased risk of infections, and potential for rare but serious side effects like leukopenia (low white blood cell count).<br><br>3. **Corticosteroids**<br>- **Mechanism**: Steroids like prednisone are used to reduce inflammation by broadly suppressing the immune system, thereby potentially reducing the activity of the complement system and inflammation in the kidneys.<br>- **Potential Side Effects**: Weight gain, increased risk of infections, diabetes, osteoporosis, mood swings, and cataracts.	C3 Glomerulopathy is a rare kidney condition where a protein called C3 builds up in the kidneys. This buildup can damage the kidneys because it's part of the body's defense system that isn't working right. There isn't a specific cure, but doctors use different medicines to help control the problem and keep the kidneys working as well as they can.<br><br>### Common Medications Used for C3 Glomerulopathy:<br><br>1. **Eculizumab**<br>- **What It Does**: This medicine stops a specific part of the body's defense system called the complement system from working too hard. It helps prevent damage to the kidney cells by blocking a protein that can hurt them.<br>- **Possible Side Effects**: It can make you more likely to get a serious type of infection called meningococcal infection. Doctors usually give a vaccine to prevent this infection before starting the medicine. Other side effects include headaches, feeling sick to your stomach, and high blood pressure.<br><br>2. **Mycophenolate Mofetil (MMF)**<br>- **What It Does**: This medicine is usually used to keep the body from rejecting a transplanted organ, but it can also help in C3 Glomerulopathy by controlling how much protein leaks into the urine and calming down the immune system.<br>- **Possible Side Effects**: It might cause stomach upset, diarrhea, and make you more likely to get infections. Sometimes, it can also lower the number of white blood cells, which help fight infections.<br><br>3. **Corticosteroids (like Prednisone)**<br>- **What It Does**: These are strong medicines that help reduce swelling and calm the immune system to prevent it from causing more damage to the kidneys.<br>- **Possible Side Effects**: They can cause weight gain, higher blood sugar, weak bones, mood changes, and make you more likely to get infections.

		### Experimental Treatments:<br><br>- **Complement Inhibitors (Other than Eculizumab)**: Newer agents targeting different parts of the complement system are being explored in clinical trials.<br>- **Plasma Exchange/Plasmapheresis**: This procedure involves removing plasma that contains harmful components, like abnormal C3 protein, and replacing it with fresh plasma or a plasma substitute. The goal is to decrease the levels of circulating factors that drive the disease.<br><br>### Monitoring and Management:<br><br>Patients with C3 Glomerulopathy require careful monitoring, given the disease's chronic nature and the potential for kidney function decline. Regular kidney function tests, urine tests for protein, and monitoring for potential side effects of medications are essential components of patient care.<br><br>### Conclusion:<br><br>Treatment for C3 Glomerulopathy involves using medications that modulate immune system activity and the complement system, aiming to reduce kidney inflammation and prevent further damage. Due to the potential for significant side effects from these treatments, regular follow-up with healthcare providers is crucial to adjust treatment as necessary and to manage any complications.	### Other Treatments Being Studied:<br><br>- **New Complement Inhibitors**: Doctors are looking into other medicines that might work better at controlling the complement system.<br>- **Plasma Exchange/Plasmapheresis**: This treatment involves taking out some of your blood plasma, which might have harmful proteins, and replacing it with good plasma. It helps lower the bad proteins that are causing kidney damage.<br><br>### Keeping Track of Your Health:<br><br>If you have C3 Glomerulopathy, you'll need regular check-ups to see how your kidneys are doing. This includes tests to check how well your kidneys are working and how much protein is in your urine. You'll also need to watch out for any side effects from the medicines.<br><br>### To Sum It Up:<br><br>Treating C3 Glomerulopathy involves using medicines to control the immune system and reduce damage to the kidneys. Because these treatments can sometimes cause side effects, it's really important to see your doctor regularly to make sure the treatments are working and to adjust them if needed.
28	C3GN	C3 Glomerulonephritis (C3GN) is a rare kidney disorder part of a larger group called C3 glomerulopathy. It is characterized by the deposition of C3 protein in the kidney's glomeruli, leading to inflammation and impaired kidney function. This condition results from dysregulation in the complement system, a part of the immune system that helps defend against pathogens but can damage the body if not properly regulated.<br><br>### Common Medications Used for C3GN:<br><br>1. **Eculizumab**<br>- **Mechanism**: Eculizumab is a monoclonal antibody that binds to the complement protein C5, preventing its cleavage into C5a and C5b, which are potent inflammatory molecules involved in the formation of the membrane attack complex (MAC). This action helps to inhibit the complement cascade, reducing inflammation and the subsequent damage to the kidney tissue.<br>- **Potential Side Effects**: The main risk is an increased susceptibility to infections, especially meningococcal meningitis. Vaccination against Neisseria meningitidis is required before starting treatment. Other side effects may include headache, nausea, and hypertension.<br><br>2. **Mycophenolate Mofetil (MMF)**<br>- **Mechanism**: MMF inhibits inosine monophosphate dehydrogenase, an enzyme critical for the proliferation of B and T lymphocytes. By suppressing these cells, MMF indirectly reduces the overall immune response, including the activation of the complement system.<br>- **Potential Side Effects**: Common side effects include gastrointestinal disturbances (such as diarrhea and abdominal pain), increased risk of infections, and potential leukopenia (reduction in white blood cell count).<br><br>3. **Corticosteroids**	C3 Glomerulonephritis (C3GN) is a rare kidney condition where a protein called C3 builds up in the kidneys, causing inflammation and damage. This happens because of problems in the immune system, specifically in a part called the complement system which usually helps fight infections but can harm the kidneys if it's not working right.<br><br>### Common Medications Used for C3GN:<br><br>1. **Eculizumab**<br>- **What It Does**: This medicine stops a specific part of the immune system called the complement system from causing too much inflammation in the kidneys. It does this by blocking a protein that plays a big role in the inflammation process.<br>- **Possible Side Effects**: The biggest risk is getting infections, especially a serious kind called meningitis. Doctors usually give a vaccine against this infection before starting the medicine. Other side effects might include headaches, feeling sick to your stomach, and higher blood pressure.<br><br>2. **Mycophenolate Mofetil (MMF)**<br>- **What It Does**: MMF weakens the immune system to reduce inflammation by stopping certain cells that are part of the immune response from growing too much.<br>- **Possible Side Effects**: This medicine can cause stomach upset, diarrhea, and make it easier for you to get infections. It can also lower the number of white blood cells, which help fight infections, making you even more prone to getting sick.<br><br>3. **Corticosteroids (like Prednisone)**<br>- **What It Does**: These are strong medicines that reduce swelling and calm down the immune system to prevent it from attacking the kidneys.<br>- **Possible Side Effects**: They can cause weight gain, higher blood sugar, high blood pressure, weak bones, increase the risk of infections, and make you feel

		- **Mechanism**: Corticosteroids such as prednisone suppress inflammation by inhibiting pathways that lead to the production of inflammatory proteins, and they also reduce the activity of the immune system broadly, including the complement system.<br>- **Potential Side Effects**: Long-term use can lead to weight gain, increased blood sugar, high blood pressure, osteoporosis, increased risk of infections, and mood swings.<br><br>### Additional Treatments:<br><br>- **Rituximab**: Sometimes used if the condition is thought to have an autoimmune component. It targets CD20 on B cells, reducing their numbers and the overall immune response.<br>- **Plasma Exchange (Plasmapheresis)**: This can be used to remove circulating antibodies and complement factors directly from the blood, which may help in cases where there is a significant autoimmune or inflammatory component.<br><br>### Monitoring and Management:<br><br>Patients with C3GN often require careful and regular monitoring to assess the effectiveness of these treatments and manage potential side effects. Regular blood tests, urine tests, and monitoring of kidney function are necessary. Adjustments in therapy may be needed based on the patient’s response and any side effects encountered.<br><br>### Conclusion:<br><br>Treating C3GN involves a combination of therapies aimed at controlling the dysregulated complement system and reducing immune system activity to prevent further damage to the kidneys. Due to the potential for significant side effects, especially from powerful immunosuppressive drugs, close monitoring by healthcare providers is crucial to ensure optimal management and to adjust treatment as necessary.	moody.<br><br>### Other Treatments That Might Help:<br><br>- **Rituximab**: This is used if doctors think your body is attacking itself (autoimmune). It targets and reduces specific immune cells, helping to calm down the immune response.<br>- **Plasma Exchange (Plasmapheresis)**: This treatment removes parts of your blood that contain harmful immune elements and replaces them with good plasma. This can help lower inflammation quickly.<br><br>### Keeping Track of Your Health:<br><br>If you have C3GN, you'll need regular check-ups to see how your kidneys are doing and how well the treatments are working. This includes blood tests, urine tests, and checks to make sure your kidneys are filtering properly. Doctors will also watch for side effects from the medicines and might change your treatment based on how you're doing.<br><br>### To Sum It Up:<br><br>Treating C3GN involves using medicines to control parts of the immune system that are causing harm to the kidneys. These treatments need to be carefully watched by doctors to make sure they're working and to keep side effects under control. Regular doctor visits are very important to keep track of your health and adjust treatments as needed.
29	Dense Deposit Disease	Dense Deposit Disease (DDD), also known as membranoproliferative glomerulonephritis type II, is a rare kidney disorder that is part of a larger group of diseases called C3 glomerulopathy. DDD is characterized by the abnormal accumulation of proteins in the kidney's glomerular basement membrane, leading to inflammation and damage. This condition results from dysregulation in the complement system, a part of the immune system.<br><br>### Common Medications Used for Dense Deposit Disease:<br><br>1. **Eculizumab**<br>- **Mechanism**: Eculizumab is a monoclonal antibody that inhibits the complement protein C5, preventing it from splitting into C5a and C5b, which are key components in the formation of the membrane attack complex (MAC) that contributes to cell damage. By inhibiting this step, eculizumab helps to reduce inflammation and damage in the kidneys.<br>- **Potential Side Effects**: The most serious risk is increased susceptibility to infections, especially meningococcal infections. Therefore, patients must receive meningococcal vaccines prior to starting treatment. Other possible side effects include headache, nausea, and hypertension.<br><br>2. **Mycophenolate Mofetil (MMF)**<br>- **Mechanism**: MMF inhibits the proliferation of B and T lymphocytes, which are types of white blood cells involved in the immune response, by blocking the production of their DNA. This suppression helps reduce the overall immune activity, including the activation of the complement system that is implicated in DDD.	Dense Deposit Disease (DDD), also known as membranoproliferative glomerulonephritis type II, is a rare kidney condition where certain proteins build up abnormally in the kidneys. This buildup can harm the kidneys' ability to filter blood and can cause inflammation. The condition is often linked to problems with the complement system, which is part of the body's immune system that helps fight infections but can damage the body if it isn't controlled properly.<br><br>### Common Medications Used for Dense Deposit Disease:<br><br>1. **Eculizumab**<br>- **What It Does**: Eculizumab is a special kind of medicine called a monoclonal antibody. It works by stopping a specific protein in the immune system called C5, which can cause damage when it splits into parts. By stopping it from splitting, this medicine helps reduce inflammation and damage in the kidneys.<br>- **Possible Side Effects**: The most serious side effect is a higher risk of getting meningococcal infections, which are very serious infections. That's why doctors give vaccines against this infection before starting the medicine. Other side effects can include headaches, feeling sick to your stomach, and high blood pressure.<br><br>2. **Mycophenolate Mofetil (MMF)**<br>- **What It Does**: MMF weakens the immune system to help calm down the inflammation by stopping certain white blood cells from growing.<br>- **Possible Side Effects**: It can cause stomach upset, diarrhea, and make you

		- **Potential Side Effects**: Common side effects include gastrointestinal issues such as diarrhea and abdominal pain, increased risk of infections, and leukopenia (a decrease in the number of white blood cells).<br><br>3. **Corticosteroids**<br>- **Mechanism**: Corticosteroids, like prednisone, are used to reduce inflammation by suppressing the immune system broadly. They help decrease the inflammatory response in the kidneys.<br>- **Potential Side Effects**: Long-term use can lead to weight gain, increased risk of infections, diabetes, osteoporosis, mood swings, and cataracts.<br><br>### Additional Treatments:<br><br>- **Rituximab**: Sometimes used for its effect on B cells, which are involved in the immune response and can contribute to the disease process in DDD.<br>- **Plasma Exchange (Plasmapheresis)**: This procedure can be used to remove certain proteins from the blood, including abnormal complement components, potentially reducing damage to the kidneys.<br><br>### Monitoring and Management:<br><br>Patients with Dense Deposit Disease require careful and regular monitoring to manage the disease and side effects of treatment. This includes frequent checks of kidney function, levels of complement proteins, and monitoring for infections. Treatment approaches can vary significantly based on the individual's response and the progression of the disease.<br><br>### Conclusion:<br><br>The treatment of Dense Deposit Disease involves a combination of medications aimed at regulating the immune system and specifically targeting the complement system to prevent further damage to the kidneys. Due to the significant potential for side effects, particularly from powerful immunosuppressive drugs, close monitoring by healthcare providers is essential to ensure effective management and to adjust treatment as necessary.	more likely to get infections. It can also lower the number of white blood cells, which makes it harder to fight off infections.<br><br>3. **Corticosteroids (like Prednisone)**<br>- **What It Does**: These are strong medicines that reduce swelling and calm down the immune system to prevent it from attacking the kidneys.<br>- **Possible Side Effects**: They can cause weight gain, higher blood sugar, high blood pressure, weak bones, increase the risk of infections, and mood changes.<br><br>### Other Treatments That Might Help:<br><br>- **Rituximab**: This medicine targets and reduces specific immune cells, helping to calm down the immune response.<br>- **Plasma Exchange (Plasmapheresis)**: This treatment involves taking out some of your blood plasma, which might have harmful proteins, and replacing it with good plasma. This can help lower the bad proteins that are causing kidney damage.<br><br>### Keeping Track of Your Health:<br><br>If you have Dense Deposit Disease, you'll need regular check-ups to see how your kidneys are doing and how well the treatments are working. This includes blood tests, urine tests, and checks to make sure your kidneys are filtering properly. Doctors will also watch for side effects from the medicines and might change your treatment based on how you're doing.<br><br>### To Sum It Up:<br><br>Treating Dense Deposit Disease involves using medicines to control the immune system and reduce damage to the kidneys. These treatments need to be carefully watched by doctors to make sure they work right and don't cause serious side effects. Regular doctor visits are very important to keep track of your health and adjust treatments as needed.
30	Atypical Hemolytic Uremic Syndrome	Atypical Hemolytic Uremic Syndrome (aHUS) is a rare disorder that leads to the abnormal destruction of red blood cells, which can clog the kidneys' filtering system, leading to severe kidney damage. It's associated with the dysregulation of the complement system, part of the immune system that, when not controlled, can attack the body's own cells.<br><br>### Common Medications Used for Atypical Hemolytic Uremic Syndrome:<br><br>1. **Eculizumab**<br>- **Mechanism**: Eculizumab is a monoclonal antibody that specifically binds to and blocks the complement protein C5, which prevents its split into C5a and C5b, important molecules in the process of inflammation and cell destruction. By stopping this split, eculizumab helps to prevent the cascade of events that lead to the destruction of red blood cells and subsequent kidney damage.<br>- **Potential Side Effects**: The most significant risk is an increased susceptibility to meningococcal infections. Therefore, vaccination against Neisseria meningitidis is required before starting treatment. Other side effects may include headache, nausea, and an increased risk of upper respiratory infections.<br><br>2. **Ravulizumab**	Atypical Hemolytic Uremic Syndrome (aHUS) is a rare disease where the body's defense system, called the complement system, mistakenly attacks its own red blood cells. This can cause serious kidney damage because the broken-down cells block the kidneys' filtering system. The treatment aims to stop this attack and protect the kidneys.<br><br>### Common Medications Used for Atypical Hemolytic Uremic Syndrome:<br><br>1. **Eculizumab**<br>- **What It Does**: Eculizumab is a special type of medicine called a monoclonal antibody. It works by blocking a part of the immune system that can cause damage when it's not working right. This medicine stops a protein called C5 from splitting into smaller parts that can hurt red blood cells and kidneys.<br>- **Possible Side Effects**: The biggest risk is getting a type of serious infection called meningococcal infection. So, doctors will give a vaccine against this infection before starting the medicine. Other side effects might include headaches, feeling nauseous, and getting upper respiratory infections more easily.<br><br>2. **Ravulizumab**

		- **Mechanism**: Similar to eculizumab, ravulizumab is a newer, long-acting monoclonal antibody that targets and inhibits C5 in the complement system. It offers similar protective effects but requires less frequent dosing compared to eculizumab.<br>- **Potential Side Effects**: Like eculizumab, the main risk is infection, particularly meningococcal infections, which necessitates vaccination. Other possible side effects are similar, including headaches, nausea, and potential hypertension.<br><br>### Additional Treatments:<br><br>- **Plasma Exchange (Plasmapheresis)**: This can be used in urgent situations to rapidly remove the harmful components of the blood, including autoantibodies and overactive complement proteins.<br>- **Immunosuppressive therapy**: In some cases, drugs like corticosteroids or rituximab might be used to reduce immune system activity further if there is an underlying autoimmune component.<br><br>### Monitoring and Management:<br><br>Patients with aHUS often require careful monitoring to manage the disease and the side effects of treatment. This includes regular checks on kidney function, blood counts, and signs of infection. Adjustment in therapy may be necessary based on the individual's response and any complications.<br><br>### Conclusion:<br><br>The primary treatment for aHUS involves medications that inhibit the complement system to prevent the destruction of red blood cells and protect the kidneys from damage. Due to the potential for serious side effects, particularly infection, regular follow-up with healthcare providers is essential to ensure the treatment is effective and to manage any arising complications. Vaccination, especially against meningococcus, is crucial before starting some of these therapies.	- **What It Does**: Ravulizumab works a lot like Eculizumab but lasts longer in the body, so you don't need to take it as often. It also stops the C5 protein from causing harm.<br>- **Possible Side Effects**: It has similar risks to Eculizumab, especially the risk of meningococcal infections, so vaccination is also necessary. Other side effects include headaches, nausea, and sometimes high blood pressure.<br><br>### Other Treatments That Might Help:<br><br>- **Plasma Exchange (Plasmapheresis)**: This treatment involves taking some blood out of the body, cleaning it from harmful substances like the overactive proteins, and then putting it back. It's especially used in urgent situations to quickly remove the harmful parts of the blood.<br>- **Immunosuppressive therapy**: Sometimes medicines like steroids or other drugs that calm the immune system are used if the disease is partly caused by the immune system being too active.<br><br>### Keeping Track of Your Health:<br><br>If you have aHUS, you'll need regular check-ups to see how your kidneys are doing and how well the treatment is working. This includes tests to check your kidney function and blood tests to make sure you're not getting too many side effects from the medicines. The doctor will adjust your treatment if needed.<br><br>### To Sum It Up:<br><br>Treating aHUS involves using special medicines to stop the part of the immune system that is damaging the red blood cells and kidneys. Because these treatments can cause serious side effects, like infections, it's really important to stay in close touch with your doctor, get the necessary vaccines, and go to all your check-ups. This helps make sure the treatment is safe and working well.
31	Monoclonal Ig-related Amyloidosis	Monoclonal Immunoglobulin-related Amyloidosis (often just called amyloidosis) is a condition where abnormal proteins, called amyloid proteins, build up in tissues and organs. These proteins are typically produced by plasma cells in the bone marrow. The buildup can lead to organ dysfunction and serious health issues. The treatment usually focuses on reducing the production of these abnormal proteins by targeting the plasma cells that produce them.<br><br>### Common Medications Used for Monoclonal Ig-related Amyloidosis:<br><br>1. **Bortezomib (Velcade)**<br>- **Mechanism**: Bortezomib is a proteasome inhibitor that works by blocking the proteasome in cells, which is responsible for breaking down proteins. By inhibiting this process, bortezomib causes the buildup of faulty proteins within plasma cells, leading to cell death. This reduces the production of abnormal proteins that form amyloid.<br>- **Potential Side Effects**: Peripheral neuropathy (nerve damage leading to tingling or numbness), fatigue, nausea, diarrhea, low blood counts, and an increased risk of infection.<br><br>2. **Lenalidomide (Revlimid)**<br>- **Mechanism**: Lenalidomide is an immunomodulatory drug that boosts the immune system to attack abnormal plasma cells. It also inhibits the growth of these cells and reduces inflammation associated with the disease.<br>- **Potential Side Effects**: Risk of blood clots, fatigue, diarrhea, constipation, rash, and	Monoclonal Immunoglobulin-related Amyloidosis (often just called amyloidosis) is a condition where abnormal proteins, called amyloid proteins, build up in the body's tissues and organs. These proteins are made by certain cells in the bone marrow called plasma cells. The buildup can cause organs to not work properly and lead to serious health problems. The main goal of treatment is to stop these cells from making too many abnormal proteins.<br><br>### Common Medications Used for Monoclonal Ig-related Amyloidosis:<br><br>1. **Bortezomib (Velcade)**<br>- **What It Does**: Bortezomib stops certain cells in the body from getting rid of damaged proteins. This makes the bad cells that produce too much amyloid protein fill up with damaged proteins and die, which helps reduce the amount of abnormal protein being made.<br>- **Possible Side Effects**: It can cause nerve damage which might feel like tingling or numbness, tiredness, feeling sick to your stomach, diarrhea, and you might catch infections more easily.<br><br>2. **Lenalidomide (Revlimid)**<br>- **What It Does**: Lenalidomide boosts the immune system to help it fight against the bad plasma cells and also stops these cells from growing. This helps

		suppression of the bone marrow leading to low blood counts.<br><br>3. **Dexamethasone**<br>- **Mechanism**: Dexamethasone is a corticosteroid that reduces inflammation and also works by suppressing the immune response, which can help reduce the number of abnormal plasma cells.<br>- **Potential Side Effects**: Increased blood sugar levels, increased risk of infections, weight gain, mood changes, and possible bone density loss with long-term use.<br><br>### Additional Treatments:<br><br>- **Cyclophosphamide**: An alkylating agent that can be used to kill fast-growing plasma cells by interfering with their DNA replication.<br>- **Stem Cell Transplantation**: In eligible patients, high-dose chemotherapy followed by a stem cell transplant can be an effective treatment to replace diseased bone marrow with healthy cells.<br>- **Monoclonal Antibodies**: Drugs like daratumumab, which target specific proteins on plasma cells, can also be used to treat amyloidosis.<br><br>### Monitoring and Management:<br><br>Patients with monoclonal Ig-related amyloidosis require close monitoring due to the potential severity of the disease and the side effects of treatments. Regular follow-ups include blood tests, organ function tests, and monitoring for signs of amyloid buildup.<br><br>### Conclusion:<br><br>Treatment for monoclonal Ig-related amyloidosis typically involves medications aimed at reducing the number of abnormal plasma cells to decrease the production of amyloid proteins. Due to the powerful nature of these medications and their potential side effects, including significant impacts on the immune system, careful management and regular monitoring by healthcare providers are crucial.	lower the amount of abnormal protein produced.<br>- **Possible Side Effects**: It can increase the risk of blood clots, make you feel tired, cause diarrhea or constipation, and cause rashes. It can also make it harder for your bone marrow to make enough blood cells.<br><br>3. **Dexamethasone**<br>- **What It Does**: Dexamethasone is a strong medicine that reduces swelling and can kill bad plasma cells when used with other treatments.<br>- **Possible Side Effects**: It can make your blood sugar go up, increase your chance of getting infections, make you gain weight, change your mood, and with long-term use, it can make your bones weaker.<br><br>### Other Treatments That Might Help:<br><br>- **Cyclophosphamide**: This medicine can kill fast-growing plasma cells by messing up their DNA, which stops them from growing.<br>- **Stem Cell Transplantation**: For some people, doctors can use high-dose medicine to kill the diseased bone marrow and then replace it with healthy stem cells.<br>- **Monoclonal Antibodies**: Drugs like daratumumab target specific proteins on the surface of plasma cells, helping to kill them and reduce the production of abnormal proteins.<br><br>### Keeping Track of Your Health:<br><br>If you have this kind of amyloidosis, you need to visit the doctor regularly. They will check how your organs are doing, how well your treatments are working, and watch for any side effects from the medicines. This includes tests like blood tests and checking how your heart and kidneys are working.<br><br>### To Sum It Up:<br><br>Treating amyloidosis involves using medicines that help reduce the number of plasma cells that make too much amyloid protein. Because these medicines are very strong and can have side effects, it's really important for doctors to check on patients often to make sure the treatment is working and to adjust it if needed.
32	Light Chain Amyloidosis	Light chain amyloidosis (AL amyloidosis) is a disease where abnormal proteins called light chains accumulate in tissues and organs, disrupting their normal function. This condition typically originates from plasma cells in the bone marrow that produce these abnormal proteins. Treatment for AL amyloidosis focuses on reducing the production of these light chains by targeting the plasma cells.<br><br>### Common Medications Used for Light Chain Amyloidosis:<br><br>1. **Bortezomib (Velcade)**<br>- **Mechanism**: Bortezomib is a proteasome inhibitor that works by blocking the proteasome in plasma cells. The proteasome is involved in breaking down proteins within the cell. By inhibiting this process, bortezomib leads to an accumulation of proteins within the plasma cells, causing them to die. This reduces the production of abnormal light chains.<br>- **Potential Side Effects**: Peripheral neuropathy (nerve damage causing tingling or numbness), fatigue, nausea, diarrhea, low blood counts, and increased risk of infection.	Light chain amyloidosis (AL amyloidosis) is a disease where abnormal proteins, called light chains, build up in tissues and organs, which can stop them from working properly. This problem starts from special cells in the bone marrow called plasma cells that make too much of these proteins. The goal of treatment is to reduce the number of these abnormal proteins by targeting the cells that make them.<br><br>### Common Medications Used for Light Chain Amyloidosis:<br><br>1. **Bortezomib (Velcade)**<br>- **What It Does**: Bortezomib is a medicine that stops certain cells in the bone marrow from getting rid of damaged proteins. Because these cells can't handle the buildup, they eventually die. This helps lower the amount of abnormal proteins being made.<br>- **Possible Side Effects**: It can cause nerve damage that feels like tingling or numbness, make you feel very tired, cause nausea or diarrhea, lower your blood counts (which makes infections more likely), and make you more likely to get sick.

		2. **Lenalidomide (Revlimid)**<br>- **Mechanism**: Lenalidomide is an immunomodulatory drug that boosts the immune system to attack abnormal plasma cells. It also inhibits the growth of these cells and reduces inflammation associated with the disease.<br>- **Potential Side Effects**: Risk of blood clots, fatigue, diarrhea, constipation, rash, and suppression of the bone marrow leading to low blood counts.<br><br>3. **Dexamethasone**<br>- **Mechanism**: Dexamethasone is a corticosteroid that reduces inflammation and also works by suppressing the immune response, which can help reduce the number of abnormal plasma cells.<br>- **Potential Side Effects**: Increased blood sugar levels, increased risk of infections, weight gain, mood changes, and possible bone density loss with long-term use.<br><br>### Additional Treatments:<br><br>- **Cyclophosphamide**: An alkylating agent used to kill fast-growing plasma cells by interfering with their DNA replication.<br>- **Daratumumab**: A monoclonal antibody that targets CD38, a protein commonly found on the surface of plasma cells. This binding leads to the death of these cells.<br>- **Stem Cell Transplantation**: In eligible patients, high-dose chemotherapy followed by a stem cell transplant can be an effective treatment to replace diseased bone marrow with healthy cells.<br><br>### Monitoring and Management:<br><br>Patients with light chain amyloidosis need careful and regular monitoring to manage the disease and side effects of treatment. This includes frequent blood tests, organ function tests, especially heart and kidney function, and monitoring for signs of amyloid buildup.<br><br>### Conclusion:<br><br>Treatment for light chain amyloidosis involves using a combination of therapies aimed at reducing the production of abnormal light chains by destroying or suppressing the plasma cells that produce them. Due to the potent nature of these medications and their potential side effects, including significant impacts on the immune system, careful management and regular monitoring by healthcare providers are crucial.	2. **Lenalidomide (Revlimid)**<br>- **What It Does**: Lenalidomide helps boost your body's immune system to fight against the abnormal cells and stops these cells from growing. It also helps reduce swelling linked with the disease.<br>- **Possible Side Effects**: It can increase the risk of blood clots, make you feel tired, cause diarrhea or constipation, and cause rashes. It can also make it harder for your bone marrow to make enough blood cells.<br><br>3. **Dexamethasone**<br>- **What It Does**: Dexamethasone is a strong medicine that reduces swelling and calms down the immune system to prevent it from causing harm to the body.<br>- **Possible Side Effects**: It can raise your blood sugar, increase your risk of infections, make you gain weight, affect your mood, and with long-term use, weaken your bones.<br><br>### Other Treatments That Might Help:<br><br>- **Cyclophosphamide**: This medicine kills fast-growing cells, including the abnormal plasma cells, by messing up their DNA.<br>- **Daratumumab**: This is a special kind of medicine that finds and sticks to certain proteins on the surface of plasma cells, leading to their destruction.<br>- **Stem Cell Transplantation**: For some people, doctors use strong medicine to destroy all the diseased bone marrow and then replace it with healthy stem cells to rebuild it.<br><br>### Keeping Track of Your Health:<br><br>If you have light chain amyloidosis, you need regular check-ups to see how your organs are doing and how well the treatments are working. This includes tests like blood tests and checks to see how well your heart and kidneys are working. Doctors will also keep an eye out for any side effects from the medicines and might change your treatment if needed.<br><br>### To Sum It Up:<br><br>Treating light chain amyloidosis involves using medicines to lower the production of abnormal proteins by destroying or controlling the plasma cells that make them. Because these treatments can be very strong and have side effects, it's really important to have regular doctor visits to make sure everything is going as planned and to adjust treatments as needed.
33	Heavy Chain Amyloidosis	Heavy chain amyloidosis (AH amyloidosis) is a rare form of amyloidosis where abnormal heavy chain proteins, produced by plasma cells, accumulate in tissues and organs, causing damage and dysfunction. Similar to other types of amyloidosis, the treatment for AH amyloidosis focuses on reducing the production of these abnormal proteins by targeting the plasma cells.<br><br>### Common Medications Used for Heavy Chain Amyloidosis:<br><br>1. **Melphalan**<br>- **Mechanism**: Melphalan is an alkylating agent that works by binding to DNA in cells, interfering with their ability to multiply. This is particularly effective against rapidly dividing cells, such as plasma cells that produce the abnormal heavy chains.	Heavy chain amyloidosis (AH amyloidosis) is a rare disease where certain proteins called heavy chains build up in the body's tissues and organs, causing damage. These proteins are made by cells in the bone marrow called plasma cells. The treatment aims to stop these cells from making too many abnormal proteins.<br><br>### Common Medications Used for Heavy Chain Amyloidosis:<br><br>1. **Melphalan**<br>- **What It Does**: Melphalan is a type of chemotherapy medicine that works by interfering with the DNA inside cells, stopping them from multiplying. It's particularly good at targeting cells that grow quickly, like the plasma cells making

		- **Potential Side Effects**: It can cause bone marrow suppression, leading to lower levels of white blood cells, red blood cells, and platelets, which increases the risk of infections, causes fatigue, and increases bleeding tendencies. Other side effects include nausea, vomiting, and risk of secondary cancers.<br><br>2. **Bortezomib (Velcade)**<br>- **Mechanism**: Bortezomib is a proteasome inhibitor that works by blocking the proteasome in plasma cells. The proteasome is a complex that breaks down proteins that the cell no longer needs or that are damaged. By inhibiting this process, bortezomib leads to an accumulation of proteins within the plasma cells, causing them to die. This helps reduce the production of abnormal heavy chains.<br>- **Potential Side Effects**: Peripheral neuropathy (nerve damage leading to tingling or numbness), fatigue, nausea, diarrhea, low blood counts, and an increased risk of infection.<br><br>3. **Dexamethasone**<br>- **Mechanism**: Dexamethasone is a corticosteroid used to reduce inflammation and suppress the immune system. It helps decrease the inflammatory response and reduces the activity of plasma cells.<br>- **Potential Side Effects**: Increased blood sugar levels, increased risk of infections, weight gain, mood changes, and possible bone density loss with long-term use.<br><br>### Additional Treatments:<br><br>- **Autologous Stem Cell Transplant**: For eligible patients, high-dose chemotherapy followed by a stem cell transplant can be an effective treatment. This procedure involves using high doses of chemotherapy to destroy the diseased bone marrow and then replacing it with the patient's own healthy stem cells to rebuild the bone marrow.<br>- **Monoclonal Antibodies**: Medications like daratumumab, which targets specific proteins on plasma cells, can also be used to treat AH amyloidosis. Daratumumab binds to CD38 on plasma cells, leading to their death.<br><br>### Monitoring and Management:<br><br>Patients with heavy chain amyloidosis need careful monitoring to manage the disease and the side effects of treatment. This includes regular blood tests, organ function tests, and monitoring for signs of amyloid buildup or organ dysfunction. Adjustments in therapy may be necessary based on the individual’s response and any complications.<br><br>### Conclusion:<br><br>The treatment of heavy chain amyloidosis involves a combination of therapies aimed at reducing the production of abnormal heavy chains by destroying or suppressing the plasma cells that produce them. Due to the potent nature of these medications and their potential side effects, close monitoring by healthcare providers is crucial to ensure optimal management and to adjust treatment as necessary.	the abnormal proteins.<br>- **Possible Side Effects**: It can make it harder for your bone marrow to make blood cells, which can lead to more infections, feeling very tired, and bleeding or bruising more easily. Other side effects include feeling sick to your stomach, throwing up, and a higher risk of getting other types of cancer later on.<br><br>2. **Bortezomib (Velcade)**<br>- **What It Does**: Bortezomib helps to kill the plasma cells by stopping them from getting rid of damaged proteins, causing these bad cells to die off. This lowers the amount of harmful proteins being made.<br>- **Possible Side Effects**: It can cause nerve damage which might feel like tingling or numbness, make you very tired, cause nausea or diarrhea, lower your blood counts (making infections more likely), and make you more likely to get sick.<br><br>3. **Dexamethasone**<br>- **What It Does**: Dexamethasone is a strong medicine that reduces swelling and calms down the immune system, which helps slow down the production of abnormal proteins.<br>- **Possible Side Effects**: It can raise your blood sugar, increase your chance of getting infections, make you gain weight, change your mood, and, if used for a long time, weaken your bones.<br><br>### Other Treatments That Might Help:<br><br>- **Autologous Stem Cell Transplant**: For some patients, doctors use strong medicine to destroy all the sick bone marrow and then replace it with healthy stem cells from the same person. This can sometimes cure the disease.<br>- **Monoclonal Antibodies**: Medicines like daratumumab attack specific targets on the plasma cells, leading to their destruction. This helps stop the production of harmful proteins.<br><br>### Keeping Track of Your Health:<br><br>If you have heavy chain amyloidosis, you need regular check-ups to see how your organs are doing and how well the treatments are working. This includes blood tests, checking your organ function, and watching for any new symptoms. Doctors will also watch out for side effects from the medicines and might change your treatment if needed.<br><br>### To Sum It Up:<br><br>Treating heavy chain amyloidosis involves using medicines to stop the plasma cells from making too many harmful proteins. These treatments are very strong and can have side effects, so it's really important to see your doctor regularly to make sure everything is going as planned and adjust treatments as needed.
34	Light and Heavy Chain Amyloidosis	The treatment for both light chain (AL) and heavy chain (AH) amyloidosis primarily focuses on reducing the production of abnormal proteins that accumulate and cause damage to tissues and organs. These conditions are caused by plasma cells in the bone marrow producing either abnormal light chains or heavy chains that form amyloid deposits.<br><br>### Common Medications Used for Light and Heavy Chain Amyloidosis:	Light chain (AL) and heavy chain (AH) amyloidosis are conditions where abnormal proteins called light chains or heavy chains build up in the body’s tissues and organs, causing damage. These proteins are made by cells in the bone marrow called plasma cells. The treatment for these diseases aims to reduce the amount of these abnormal proteins by targeting the cells that make them.<br><br>### Common Medications Used for Light and Heavy Chain Amyloidosis:

		1. **Bortezomib (Velcade)**<br>- **Mechanism**: Bortezomib is a proteasome inhibitor that blocks the proteasome in plasma cells. The proteasome is a complex that degrades unneeded or damaged proteins. Inhibiting the proteasome leads to the accumulation of proteins inside the plasma cells, causing cell stress and death. This reduction in plasma cells decreases the production of abnormal proteins.<br>- **Potential Side Effects**: Peripheral neuropathy (nerve damage leading to tingling or numbness), fatigue, nausea, diarrhea, low blood counts, and an increased risk of infection.<br><br>2. **Lenalidomide (Revlimid) and Thalidomide**<br>- **Mechanism**: These are immunomodulatory drugs (IMiDs) that help regulate the immune system. They enhance the immune response against abnormal plasma cells and inhibit their growth and survival. They also modulate the tumor microenvironment.<br>- **Potential Side Effects**: Risk of blood clots, fatigue, rash, neutropenia (low white blood cell count), and potential for birth defects if used during pregnancy.<br><br>3. **Dexamethasone and other corticosteroids**<br>- **Mechanism**: Corticosteroids are anti-inflammatory and immunosuppressive agents. They reduce inflammation and act directly on plasma cells to reduce their proliferation and induce apoptosis (cell death).<br>- **Potential Side Effects**: Increased blood sugar, increased risk of infections, weight gain, mood swings, and with long-term use, bone loss and muscle weakness.<br><br>4. **Melphalan**<br>- **Mechanism**: Melphalan is an alkylating agent used in chemotherapy. It works by cross-linking DNA strands in cells, preventing cell replication and leading to cell death. This is particularly effective in targeting rapidly dividing plasma cells producing amyloid proteins.<br>- **Potential Side Effects**: Bone marrow suppression, increased susceptibility to infections, nausea, vomiting, and secondary cancers.<br><br>5. **Daratumumab (Darzalex)**<br>- **Mechanism**: Daratumumab is a monoclonal antibody that targets CD38, a protein expressed on the surface of plasma cells. By binding to CD38, daratumumab triggers immune-mediated responses that lead to the destruction of plasma cells.<br>- **Potential Side Effects**: Infusion-related reactions (such as fever, chills, and breathlessness), fatigue, nausea, and effects on blood cell counts.<br><br>### Additional Treatments:<br><br>- **Autologous Stem Cell Transplant**: This procedure involves high-dose chemotherapy followed by transplantation of the patient's own stem cells to reset the bone marrow and reduce the population of plasma cells producing amyloid proteins.<br>- **Cyclophosphamide**: Another alkylating agent that can be used in combination with other treatments to enhance the effectiveness against plasma cells.<br><br>### Monitoring and Management:<br><br>Patients treated for AL and AH amyloidosis require careful monitoring. Regular follow-ups include blood tests to monitor organ function and blood cell counts, heart and kidney function assessments, and imaging studies if necessary. Treatment plans are frequently adjusted based on the patient's response to therapy and the presence of any side effects.	1. **Bortezomib (Velcade)**<br>- **What It Does**: Bortezomib stops certain cells in the bone marrow from getting rid of damaged proteins. Because these cells can't handle the buildup, they eventually die. This helps lower the amount of abnormal proteins being made.<br>- **Possible Side Effects**: It can cause nerve damage which might feel like tingling or numbness, make you very tired, cause nausea or diarrhea, lower your blood counts (which makes infections more likely), and make you more likely to get sick.<br><br>2. **Lenalidomide (Revlimid) and Thalidomide**<br>- **What It Does**: These medicines help regulate the immune system. They work by making the immune system stronger against the abnormal cells and stop these cells from growing.<br>- **Possible Side Effects**: There's a risk of blood clots, feeling very tired, getting skin rashes, and having lower than normal white blood cell counts which can make it easier to catch infections.<br><br>3. **Dexamethasone and other corticosteroids**<br>- **What It Does**: These are strong medicines that reduce swelling and calm down the immune system to prevent it from causing harm to the body.<br>- **Possible Side Effects**: They can raise your blood sugar, increase your chance of getting infections, make you gain weight, change your mood, and, if used for a long time, weaken your bones and muscles.<br><br>4. **Melphalan**<br>- **What It Does**: Melphalan is a chemotherapy drug that kills fast-growing cells, including the abnormal plasma cells producing harmful proteins.<br>- **Possible Side Effects**: It can make it hard for your bone marrow to make blood cells, increase your risk of infections, make you feel sick to your stomach, and lead to vomiting.<br><br>5. **Daratumumab (Darzalex)**<br>- **What It Does**: Daratumumab finds and sticks to certain proteins on the surface of plasma cells, leading to their destruction.<br>- **Possible Side Effects**: It can cause reactions when you get the infusion, like fever, chills, and feeling short of breath. It can also make you feel tired, nauseous, and affect your blood cell counts.<br><br>### Other Treatments That Might Help:<br><br>- **Autologous Stem Cell Transplant**: This treatment uses strong medicines to destroy all the sick bone marrow and then replaces it with healthy stem cells from the same person. This can sometimes cure the disease.<br><br>### Keeping Track of Your Health:<br><br>If you have light chain or heavy chain amyloidosis, you need regular check-ups to see how your organs are doing and how well the treatments are working. This includes tests like blood tests and checks to see how well your heart and kidneys are working. Doctors will also keep an eye out for any side effects from the medicines and might change your treatment if needed.

		### Conclusion:<br><br>Treatment for light and heavy chain amyloidosis involves a combination of medications aimed at reducing the number of abnormal plasma cells and hence the production of amyloid proteins. Due to the potent nature of these medications and their possible side effects, regular monitoring by healthcare providers is essential to manage the disease effectively.	### To Sum It Up:<br><br>Treating light and heavy chain amyloidosis involves using medicines to lower the production of abnormal proteins by destroying or controlling the plasma cells that make them. Because these treatments can be very strong and have side effects, it's really important to have regular doctor visits to make sure everything is going as planned and adjust treatments as needed.
35	Fibrillary GN	Fibrillary glomerulonephritis (Fibrillary GN) is a rare kidney disease characterized by the deposition of fibrillary proteins in the glomeruli, the filtering units of the kidneys. These protein deposits lead to inflammation and damage, impairing kidney function. Treatment for Fibrillary GN often involves medications aimed at managing symptoms and slowing the progression of the disease, as no specific therapy directly targets the underlying cause of fibrillary deposition.<br><br>### Common Medications Used for Fibrillary GN:<br><br>1. **Corticosteroids (such as Prednisone)**<br>- **Mechanism**: Corticosteroids are potent anti-inflammatory drugs that reduce inflammation in the kidneys by suppressing the immune system. They help decrease the inflammatory response that contributes to kidney damage.<br>- **Potential Side Effects**: Long-term use of corticosteroids can lead to weight gain, increased blood sugar, high blood pressure, increased risk of infections, bone density loss, and mood swings.<br><br>2. **Cyclophosphamide**<br>- **Mechanism**: Cyclophosphamide is an immunosuppressive agent that kills rapidly dividing cells, including immune cells that may be contributing to inflammation and damage in the kidneys.<br>- **Potential Side Effects**: Increased risk of infections, bone marrow suppression (which can lead to low blood cell counts), hair loss, bladder toxicity, and potential infertility.<br><br>3. **Mycophenolate Mofetil (MMF)**<br>- **Mechanism**: MMF inhibits the proliferation of B and T lymphocytes, which are part of the immune response involved in causing inflammation in the kidneys.<br>- **Potential Side Effects**: Common side effects include gastrointestinal issues such as diarrhea, increased risk of infections, and possible leukopenia (a decrease in white blood cells).<br><br>### Additional Treatments:<br><br>- **Rituximab**: Sometimes used for its potential to target B cells, which are involved in the autoimmune response that can contribute to kidney damage. Rituximab is a monoclonal antibody that depletes CD20-positive B cells.<br>- **Angiotensin-converting enzyme (ACE) inhibitors or Angiotensin II receptor blockers (ARBs)**: These drugs are used to manage blood pressure and proteinuria (protein in urine), which are common in patients with kidney diseases. They help protect kidney function by reducing blood pressure and decreasing the workload on the kidneys.<br>- **Potential Side Effects**: Dizziness, elevated blood potassium levels, and, in rare cases, kidney function deterioration.<br><br>### Monitoring and Management:	Fibrillary glomerulonephritis (Fibrillary GN) is a rare kidney condition where abnormal protein fibers build up in the kidneys' filters, called glomeruli. This buildup can cause inflammation and damage, making it hard for the kidneys to work properly. The treatments mainly focus on managing symptoms and slowing down the damage because there isn't a specific cure for the protein buildup itself.<br><br>### Common Medications Used for Fibrillary GN:<br><br>1. **Corticosteroids (like Prednisone)**<br>- **What They Do**: These are strong medicines that help reduce swelling and calm down the immune system to prevent it from attacking the kidneys.<br>- **Possible Side Effects**: If you take these medicines for a long time, they can cause weight gain, higher blood sugar, high blood pressure, make you more likely to get infections, weaken your bones, and affect your mood.<br><br>2. **Cyclophosphamide**<br>- **What It Does**: This medicine works by killing fast-growing cells, including those that are part of the immune system and causing inflammation in the kidneys.<br>- **Possible Side Effects**: It can increase your risk of getting infections, make your hair fall out, cause problems in your bladder, and reduce the number of blood cells, which can make you feel very tired and increase the risk of bleeding or bruising.<br><br>3. **Mycophenolate Mofetil (MMF)**<br>- **What It Does**: MMF slows down the growth of certain immune cells that are involved in the inflammation in the kidneys.<br>- **Possible Side Effects**: The most common side effects include stomach upset, diarrhea, and a higher chance of getting infections. It can also lower the number of white blood cells, which helps your body fight infections.<br><br>### Additional Treatments:<br><br>- **Rituximab**: This medicine targets specific immune cells, helping to reduce the number of cells that can cause inflammation in the kidneys.<br>- **ACE Inhibitors or ARBs**: These are blood pressure medicines that also help reduce protein loss in urine, which can help protect the kidneys from more damage.<br>- **Possible Side Effects**: These can make you feel dizzy, raise the potassium levels in your blood, and in rare cases, affect how well your kidneys work.<br><br>### Keeping Track of Your Health:<br><br>If you have Fibrillary GN, you need to see your doctor regularly to check how well your kidneys are working and to keep an eye on your blood pressure and urine protein levels. Your doctor will also check for side effects from your medicines and

		Patients with Fibrillary GN require regular monitoring to manage the disease and side effects of the treatment. This includes frequent kidney function tests, blood pressure monitoring, and checks for protein in the urine. Adjustments in therapy may be necessary based on the patient's response and the progression of the disease.<br><br>### Conclusion:<br><br>The treatment of Fibrillary GN involves using a combination of medications aimed at reducing inflammation and suppressing the immune response to slow down the progression of kidney damage. Due to the significant potential for side effects from these treatments, close monitoring by healthcare providers is essential to ensure optimal management and to adjust treatment as necessary.	might change your treatment based on how you're doing.<br><br>### To Sum It Up:<br><br>Treating Fibrillary GN involves using medicines to reduce inflammation and calm the immune system to slow down damage to the kidneys. Since these treatments can have strong side effects, it's really important to have regular check-ups with your doctor to make sure the treatment is working and to adjust it if needed.
36	Cryoglobulinemic GN	Cryoglobulinemic glomerulonephritis (Cryoglobulinemic GN) is a type of kidney inflammation that occurs as a complication of cryoglobulinemia, a condition characterized by the presence of abnormal proteins (cryoglobulins) in the blood that can precipitate at low temperatures. This disease often involves the immune system and can be associated with infections like hepatitis C, autoimmune diseases, or cancers. Treatment strategies for Cryoglobulinemic GN typically focus on reducing inflammation, managing the underlying cause, and controlling the immune system's production of cryoglobulins.<br><br>### Common Medications Used for Cryoglobulinemic GN:<br><br>1. **Corticosteroids (such as Prednisone)**<br>- **Mechanism**: Corticosteroids are potent anti-inflammatory agents that broadly suppress the immune system, reducing inflammation and the production of cryoglobulins.<br>- **Potential Side Effects**: Increased risk of infections, high blood sugar levels, high blood pressure, weight gain, mood swings, and, with long-term use, osteoporosis.<br><br>2. **Rituximab**<br>- **Mechanism**: Rituximab is a monoclonal antibody that targets CD20, a protein found on the surface of B cells (a type of white blood cell involved in the immune response). By depleting B cells, rituximab helps reduce the production of cryoglobulins and decreases inflammation.<br>- **Potential Side Effects**: Infusion reactions (like chills, fever, nausea), increased risk of infections, and, rarely, severe skin and brain infections.<br><br>3. **Cyclophosphamide**<br>- **Mechanism**: Cyclophosphamide is a cytotoxic drug that suppresses the immune system by killing rapidly dividing cells, including those that contribute to inflammation and antibody production.<br>- **Potential Side Effects**: Increased risk of infections, bone marrow suppression (leading to low blood cell counts), hair loss, and bladder toxicity.<br><br>### Additional Treatments:<br><br>- **Plasma Exchange (Plasmapheresis)**: This procedure can be used to remove cryoglobulins directly from the blood, reducing their levels and alleviating symptoms.<br>- **Antiviral Therapy**: For patients whose cryoglobulinemic GN is associated with hepatitis C, antiviral medications (like direct-acting antivirals for hepatitis C) can effectively reduce viral load, which in turn decreases the immune stimulation leading to cryoglobulin production.<br>- **Immunosuppressive Agents**: In cases where rituximab and cyclophosphamide are not sufficient, other immunosuppressants like azathioprine or mycophenolate mofetil may be	Cryoglobulinemic glomerulonephritis (Cryoglobulinemic GN) is a kind of kidney inflammation caused by abnormal proteins in the blood called cryoglobulins. These proteins can clump together in cold temperatures, leading to blockages and inflammation in the kidneys. This condition often involves the immune system and can be linked to other health issues like hepatitis C, autoimmune diseases, or certain cancers. The treatment focuses on reducing inflammation, handling the underlying cause, and stopping the immune system from making too many cryoglobulins.<br><br>### Common Medications Used for Cryoglobulinemic GN:<br><br>1. **Corticosteroids (like Prednisone)**<br>- **What They Do**: These are strong medicines that reduce swelling and calm down the immune system to stop it from causing harm to the kidneys.<br>- **Possible Side Effects**: They can make you more likely to get infections, raise your blood sugar, increase your blood pressure, cause weight gain, change your mood, and, if used for a long time, weaken your bones.<br><br>2. **Rituximab**<br>- **What It Does**: Rituximab is a special kind of medicine that targets specific cells of your immune system that are part of the problem, reducing their numbers and helping to calm down the immune reaction.<br>- **Possible Side Effects**: You might feel unwell during the treatment (like getting a fever or feeling tired), and there's a higher chance of getting infections. Rarely, it can cause serious skin and brain infections.<br><br>3. **Cyclophosphamide**<br>- **What It Does**: This medicine helps by stopping certain immune cells from growing too fast, which can help decrease the harmful proteins that cause kidney problems.<br>- **Possible Side Effects**: It can increase your risk of getting infections, make your hair fall out, cause problems in your bladder, and lower your blood counts, which can make you feel very tired and increase the risk of bleeding or bruising.<br><br>### Additional Treatments:<br><br>- **Plasma Exchange (Plasmapheresis)**: This treatment involves removing some of your blood, cleaning it from harmful proteins, and then putting it back. This can help lower the bad proteins that are causing kidney damage.<br>- **Antiviral Therapy**: If the cryoglobulinemic GN is linked to hepatitis C, doctors might use medicines that fight the virus, which can help reduce the immune

		used.<br><br>### Monitoring and Management:<br><br>Patients with cryoglobulinemic GN require regular monitoring to manage the disease and monitor for side effects of treatment. This includes regular kidney function tests, blood tests to measure cryoglobulin levels, and monitoring for signs of infection or other complications. Adjustments in therapy may be necessary based on the patient's response and any side effects encountered.<br><br>### Conclusion:<br><br>Treatment for cryoglobulinemic GN involves a combination of therapies aimed at reducing inflammation, managing the underlying cause, and controlling the immune system's activity. Due to the complexity of this disease and the potential for significant side effects from treatment, close monitoring by healthcare providers is essential to ensure effective management and to adjust treatment as necessary.	system's overreaction.<br>- **Other Immune System Medicines**: Sometimes, if the other treatments aren't enough, doctors might use other medicines that help calm the immune system.<br><br>### Keeping Track of Your Health:<br><br>If you have cryoglobulinemic GN, you need to see your doctor regularly to check how your kidneys are doing and how well the treatments are working. This includes tests to check your kidney function and blood tests to keep an eye on the levels of cryoglobulins. Your doctor will also watch for any side effects from the medicines and might change your treatment if needed.<br><br>### To Sum It Up:<br><br>Treating cryoglobulinemic GN involves using medicines to reduce swelling, handle the root cause, and control the immune system. These treatments need to be carefully watched by doctors to make sure they're working right and to adjust them if needed. Regular doctor visits are very important to keep track of your health and manage the treatment.
37	Immunotactoid GN	Immunotactoid glomerulonephritis (Immunotactoid GN) is a rare kidney disorder characterized by the deposition of organized microtubular monoclonal immunoglobulins in the glomeruli, which are the filtering units in the kidneys. It's similar to fibrillary GN but has distinct features under microscopic examination. The treatment of Immunotactoid GN often targets the underlying cause, which can include plasma cell disorders or lymphoproliferative disorders, and aims to reduce the production of abnormal immunoglobulins.<br><br>### Common Medications Used for Immunotactoid GN:<br><br>1. **Corticosteroids (such as Prednisone)**<br>- **Mechanism**: Corticosteroids are anti-inflammatory drugs that broadly suppress the immune system, reducing inflammation and antibody production by plasma cells, which may contribute to the disease.<br>- **Potential Side Effects**: Long-term use can lead to weight gain, increased blood sugar levels, high blood pressure, increased risk of infections, osteoporosis, and mood swings.<br><br>2. **Cyclophosphamide**<br>- **Mechanism**: Cyclophosphamide is a cytotoxic drug that suppresses the immune system by preventing the proliferation of rapidly dividing cells, including lymphocytes and plasma cells, which are responsible for producing the monoclonal immunoglobulins.<br>- **Potential Side Effects**: Increased risk of infections, bone marrow suppression (leading to low blood cell counts), hair loss, and bladder toxicity. There is also a potential risk of secondary cancers.<br><br>3. **Rituximab**<br>- **Mechanism**: Rituximab is a monoclonal antibody that targets CD20, a protein located on the surface of B-cells (a type of white blood cell involved in the immune response). By binding to CD20, rituximab helps to deplete these cells, thereby reducing the production of pathological immunoglobulins.<br>- **Potential Side Effects**: Infusion reactions (such as fever, chills, nausea), increased risk of infections, and rarely, severe skin and brain infections.<br><br>### Additional Treatments:	Immunotactoid glomerulonephritis (Immunotactoid GN) is a rare kidney condition where abnormal protein fibers build up in the kidney's filters, called glomeruli. This buildup can harm the kidneys, making it hard for them to clean the blood properly. The treatment usually focuses on dealing with the underlying cause, which might involve problems with plasma cells (a type of blood cell) that make too much of these proteins.<br><br>### Common Medications Used for Immunotactoid GN:<br><br>1. **Corticosteroids (like Prednisone)**<br>- **What They Do**: These are strong medicines that help reduce swelling and calm down the immune system to prevent it from causing harm to the kidneys.<br>- **Possible Side Effects**: They can make you more likely to get infections, raise your blood sugar, increase your blood pressure, cause weight gain, change your mood, and, if used for a long time, weaken your bones.<br><br>2. **Cyclophosphamide**<br>- **What It Does**: This medicine helps by stopping certain immune cells from growing too fast, which can help decrease the harmful proteins that cause kidney problems.<br>- **Possible Side Effects**: It can increase your risk of getting infections, make your hair fall out, cause problems in your bladder, and lower your blood counts, which can make you feel very tired and increase the risk of bleeding or bruising.<br><br>3. **Rituximab**<br>- **What It Does**: Rituximab targets specific immune cells, helping to reduce the number of cells that can cause inflammation in the kidneys.<br>- **Possible Side Effects**: You might have reactions like fever or tiredness when getting the medicine, and there's a higher chance of getting infections. Rarely, it can cause serious skin and brain infections.<br><br>### Additional Treatments:

		- **Plasma Exchange (Plasmapheresis)**: This procedure may be used to remove the circulating monoclonal immunoglobulins from the blood, reducing their deposition in the glomeruli and alleviating symptoms.<br>- **Immunosuppressive Agents**: Other immunosuppressants like mycophenolate mofetil or azathioprine may be used depending on the patient's response to initial treatments and the underlying cause.<br><br>### Monitoring and Management:<br><br>Patients with Immunotactoid GN require regular monitoring to assess the effectiveness of the treatment and manage potential side effects. This includes routine kidney function tests, urine tests for protein, blood tests to monitor immune cell counts, and assessments for signs of infection or other complications. Therapy may need adjustments based on the individual's response and progression of the disease.<br><br>### Conclusion:<br><br>The treatment of Immunotactoid GN involves a combination of therapies aimed at reducing the production of abnormal immunoglobulins and controlling the underlying lymphoproliferative disorder, if present. Due to the potent nature of these medications and their potential side effects, close monitoring by healthcare providers is essential to ensure effective management and to adjust treatment as necessary.	- **Plasma Exchange (Plasmapheresis)**: This treatment involves removing some of your blood, cleaning it from harmful proteins, and then putting it back. This can help lower the bad proteins that are causing kidney damage.<br>- **Other Immune System Medicines**: Sometimes, if the other treatments aren't enough, doctors might use other medicines that help calm the immune system.<br><br>### Keeping Track of Your Health:<br><br>If you have Immunotactoid GN, you need to see your doctor regularly to check how your kidneys are doing and how well the treatments are working. This includes tests to check your kidney function and blood tests to keep an eye on the levels of proteins and immune cells. Your doctor will also watch for any side effects from the medicines and might change your treatment if needed.<br><br>### To Sum It Up:<br><br>Treating Immunotactoid GN involves using medicines to reduce swelling, handle the root cause, and control the immune system. These treatments need to be carefully watched by doctors to make sure they're working right and to adjust them if needed. Regular doctor visits are very important to keep track of your health and manage the treatment.
38	Proliferative Glomerulonephritis With Monoclonal Ig Deposits	Proliferative Glomerulonephritis with Monoclonal Immunoglobulin Deposits (PGNMID) is a rare kidney disorder characterized by the deposition of monoclonal immunoglobulins (a type of protein made by plasma cells) in the kidney's glomeruli, the tiny structures within the kidneys that filter blood. This can lead to inflammation and damage, affecting kidney function. The treatment often involves targeting the plasma cells producing these abnormal proteins.<br><br>### Common Medications Used for PGNMID:<br><br>1. **Bortezomib (Velcade)**<br>- **Mechanism**: Bortezomib is a proteasome inhibitor that works by blocking the proteasome in plasma cells. The proteasome is involved in degrading damaged or unneeded proteins. Inhibiting this process leads to an accumulation of proteins within plasma cells, causing cell stress and death. This reduces the production of abnormal monoclonal proteins.<br>- **Potential Side Effects**: Peripheral neuropathy (nerve damage leading to tingling or numbness), fatigue, nausea, diarrhea, low blood counts, and an increased risk of infection.<br><br>2. **Cyclophosphamide**<br>- **Mechanism**: Cyclophosphamide is a chemotherapy agent that works by damaging the DNA of rapidly dividing cells, including plasma cells. This prevents them from reproducing, which reduces the number of cells producing abnormal immunoglobulins.<br>- **Potential Side Effects**: Increased risk of infections, bone marrow suppression (leading to low blood cell counts), hair loss, and potential bladder toxicity. Long-term use also increases the risk of secondary cancers.<br><br>3. **Dexamethasone**<br>- **Mechanism**: Dexamethasone is a corticosteroid used to reduce inflammation and suppress the immune system. It acts by decreasing the production of inflammatory chemicals and can induce death in certain immune cells, including plasma cells.<br>- **Potential Side Effects**: Increased blood sugar levels, increased risk of infections, weight	Proliferative Glomerulonephritis with Monoclonal Immunoglobulin Deposits (PGNMID) is a rare kidney condition where abnormal proteins, called monoclonal immunoglobulins, build up in the kidneys. These proteins are made by certain cells in the bone marrow called plasma cells. The buildup can cause inflammation and damage in the kidneys, affecting their ability to filter blood properly. Treatment usually targets these plasma cells to stop them from making the abnormal proteins.<br><br>### Common Medications Used for PGNMID:<br><br>1. **Bortezomib (Velcade)**<br>- **What It Does**: Bortezomib stops the plasma cells from getting rid of damaged proteins, causing these cells to stress and die. This helps lower the amount of abnormal proteins being made.<br>- **Possible Side Effects**: It can cause nerve damage that might feel like tingling or numbness, make you feel very tired, cause nausea or diarrhea, lower your blood counts (which makes infections more likely), and make you more likely to get sick.<br><br>2. **Cyclophosphamide**<br>- **What It Does**: This is a strong medicine that kills fast-growing cells, including plasma cells that are making the wrong proteins.<br>- **Possible Side Effects**: It can increase your risk of getting infections, make your hair fall out, cause problems in your bladder, and lower your blood counts, which can make you feel very tired and increase the risk of bleeding or bruising. It also raises the risk of getting other types of cancer if used for a long time.<br><br>3. **Dexamethasone**<br>- **What It Does**: Dexamethasone is a medicine that reduces swelling and calms down the immune system to prevent it from causing harm to the kidneys.<br>- **Possible Side Effects**: It can raise your blood sugar, increase your chance of

		gain, mood changes, and with long-term use, osteoporosis and muscle weakness.<br><br>### Additional Treatments:<br><br>- **Rituximab (Rituxan)**<br>- **Mechanism**: Rituximab is a monoclonal antibody that specifically targets CD20, a protein found on the surface of B-cells, which are precursor cells to plasma cells. By depleting these B-cells, rituximab reduces the overall number of plasma cells available to produce the monoclonal immunoglobulins.<br>- **Potential Side Effects**: Infusion reactions (such as chills, fever, nausea), increased risk of infections, and rarely, severe skin and brain infections.<br><br>- **Immunosuppressive Agents**: Depending on the severity and the patient's response to initial treatments, other immunosuppressants like mycophenolate mofetil or azathioprine may be used to further suppress immune activity.<br><br>### Monitoring and Management:<br><br>Patients with PGNMID require careful monitoring to assess the effectiveness of treatment and manage any side effects. Regular follow-ups include kidney function tests, urine tests for protein, and blood tests to monitor immune cell levels and signs of infection. Adjustments in therapy may be necessary based on the patient's response and any complications.<br><br>### Conclusion:<br><br>Treatment for PGNMID involves a combination of therapies aimed at reducing the production of abnormal monoclonal proteins by destroying or suppressing the plasma cells that produce them. Due to the potent nature of these medications and their potential side effects, close monitoring by healthcare providers is crucial to ensure effective management and to adjust treatment as necessary.	getting infections, make you gain weight, change your mood, and, if used for a long time, weaken your bones and muscles.<br><br>### Additional Treatments:<br><br>- **Rituximab (Rituxan)**<br>- **What It Does**: Rituximab targets and destroys specific cells that are part of the problem before they turn into plasma cells that make the abnormal proteins.<br>- **Possible Side Effects**: You might have reactions like fever or feeling tired during the treatment, and there's a higher chance of getting infections. Rarely, it can cause serious skin and brain infections.<br><br>### Keeping Track of Your Health:<br><br>If you have PGNMID, you need regular check-ups to see how your kidneys are doing and how well the treatments are working. This includes tests to check your kidney function and blood tests to keep an eye on the levels of proteins and immune cells. Your doctor will also watch for any side effects from the medicines and might change your treatment if needed.<br><br>### To Sum It Up:<br><br>Treating PGNMID involves using medicines to reduce the number of plasma cells making the harmful proteins. Since these treatments can be very strong and have side effects, it's really important to have regular doctor visits to make sure everything is going as planned and to adjust treatments as needed.
39	Monoclonal Ig Deposition Disease	Monoclonal Immunoglobulin Deposition Disease (MIDD) is a condition where abnormal proteins (monoclonal immunoglobulins) produced by plasma cells build up in various tissues of the body, including the kidneys, heart, and nerves. This buildup can interfere with the normal function of these organs. The treatment typically involves targeting the plasma cells that produce these abnormal proteins to reduce their levels and prevent further organ damage.<br><br>### Common Medications Used for Monoclonal Ig Deposition Disease:<br><br>1. **Bortezomib (Velcade)**<br>- **Mechanism**: Bortezomib is a proteasome inhibitor that blocks the proteasome in plasma cells. The proteasome is a complex in cells that degrades unneeded or damaged proteins. By inhibiting this process, bortezomib causes the accumulation of proteins within the plasma cells, leading to their death. This reduces the production of abnormal monoclonal immunoglobulins.<br>- **Potential Side Effects**: Peripheral neuropathy (nerve damage causing tingling or numbness), fatigue, nausea, diarrhea, low blood counts, and increased risk of infections.<br><br>2. **Melphalan**<br>- **Mechanism**: Melphalan is a chemotherapy agent that works by cross-linking the DNA strands in cells, thereby inhibiting DNA replication and RNA transcription. This action prevents the proliferation of plasma cells that produce the abnormal proteins.<br>- **Potential Side Effects**: Bone marrow suppression (leading to decreased blood cell	Monoclonal Immunoglobulin Deposition Disease (MIDD) is a condition where abnormal proteins made by certain cells in the bone marrow build up in different parts of the body, like the kidneys, heart, and nerves. This can cause these organs to not work properly. The treatment focuses on stopping these cells from making too many abnormal proteins.<br><br>### Common Medications Used for Monoclonal Ig Deposition Disease:<br><br>1. **Bortezomib (Velcade)**<br>- **What It Does**: Bortezomib stops specific cells in the bone marrow from getting rid of damaged proteins, causing these cells to die. This lowers the amount of abnormal proteins being made.<br>- **Possible Side Effects**: It can cause nerve damage which might feel like tingling or numbness, make you very tired, cause nausea or diarrhea, lower your blood counts (which makes infections more likely), and make you more likely to get sick.<br><br>2. **Melphalan**<br>- **What It Does**: Melphalan is a type of chemotherapy that stops cells from copying their DNA, preventing them from multiplying. This is especially effective against cells that produce the abnormal proteins.<br>- **Possible Side Effects**: It can lower the number of blood cells, which increases

		counts), increased risk of infections, nausea, vomiting, and potential for secondary cancers.<br><br>3. **Dexamethasone**<br>- **Mechanism**: Dexamethasone is a corticosteroid used to reduce inflammation and suppress the immune system. It decreases the production of inflammatory chemicals and can induce apoptosis (programmed cell death) in certain immune cells, including plasma cells.<br>- **Potential Side Effects**: Increased blood sugar levels, increased risk of infections, weight gain, mood changes, and with long-term use, osteoporosis and muscle weakness.<br><br>### Additional Treatments:<br><br>- **Rituximab (Rituxan)**<br>- **Mechanism**: Rituximab is a monoclonal antibody that targets CD20, a protein found on the surface of B cells (precursors to plasma cells). By binding to CD20, rituximab helps to deplete these cells, reducing the overall number of plasma cells and hence the production of monoclonal immunoglobulins.<br>- **Potential Side Effects**: Infusion reactions (such as fever, chills, nausea), increased risk of infections, and rarely, severe skin and brain infections.<br><br>- **Autologous Stem Cell Transplantation**<br>- **Mechanism**: For some patients, particularly those who are younger and in good health, high-dose chemotherapy followed by an autologous stem cell transplant may be used. This procedure involves using high doses of chemotherapy to destroy the diseased bone marrow and then replacing it with the patient's own healthy stem cells to rebuild the bone marrow.<br>- **Potential Side Effects**: High risk of infections, effects from high-dose chemotherapy, and complications related to stem cell transplantation.<br><br>### Monitoring and Management:<br><br>Patients with MIDD require regular monitoring to assess the effectiveness of the treatment and manage any side effects. This includes routine checks on kidney function, blood tests to monitor blood cell counts, and imaging studies if necessary. Adjustments in therapy may be necessary based on the patient’s response and any side effects encountered.<br><br>### Conclusion:<br><br>The treatment of MIDD involves a combination of therapies aimed at reducing the production of abnormal monoclonal immunoglobulins by destroying or suppressing the plasma cells that produce them. Due to the potent nature of these medications and their potential side effects, close monitoring by healthcare providers is crucial to ensure effective management and to adjust treatment as necessary.	the risk of infections, make you feel sick to your stomach, and might cause vomiting. There’s also a small chance it could lead to other types of cancer later on.<br><br>3. **Dexamethasone**<br>- **What It Does**: Dexamethasone is a strong medicine that reduces swelling and calms down the immune system to prevent it from causing harm to the body.<br>- **Possible Side Effects**: It can raise your blood sugar, increase your chance of getting infections, make you gain weight, change your mood, and, if used for a long time, weaken your bones and muscles.<br><br>### Additional Treatments:<br><br>- **Rituximab (Rituxan)**<br>- **What It Does**: Rituximab targets specific immune cells, helping to reduce the number of cells that can cause inflammation and produce abnormal proteins.<br>- **Possible Side Effects**: You might have reactions like fever or feeling tired during the treatment, and there’s a higher chance of getting infections. Rarely, it can cause serious skin and brain infections.<br><br>- **Autologous Stem Cell Transplantation**<br>- **What It Does**: For some patients, doctors use strong medicine to destroy all the sick bone marrow and then replace it with the patient's own healthy stem cells. This can sometimes cure the disease.<br>- **Possible Side Effects**: High risk of infections, effects from strong chemotherapy, and complications from the transplant process.<br><br>### Keeping Track of Your Health:<br><br>If you have MIDD, you need regular check-ups to see how your organs are doing and how well the treatments are working. This includes tests to check your kidney function and blood tests to keep an eye on the levels of proteins and immune cells. Your doctor will also watch for any side effects from the medicines and might change your treatment if needed.<br><br>### To Sum It Up:<br><br>Treating MIDD involves using medicines to reduce the number of cells making the harmful proteins. Since these treatments can be very strong and have side effects, it’s really important to have regular doctor visits to make sure everything is going as planned and adjust treatments as needed.
40	Light Chain Deposition Disease	Light Chain Deposition Disease (LCDD) is a condition in which abnormal light chain proteins produced by plasma cells in the bone marrow accumulate in various tissues, primarily affecting the kidneys. This accumulation can lead to kidney dysfunction and other organ issues. Treatment focuses on reducing the production of these abnormal light chains by targeting the plasma cells.<br><br>### Common Medications Used for Light Chain Deposition Disease:<br><br>1. **Bortezomib (Velcade)**<br>- **Mechanism**: Bortezomib is a proteasome inhibitor that works by blocking the proteasome in plasma cells. The proteasome is responsible for breaking down unneeded or	Light Chain Deposition Disease (LCDD) is a condition where abnormal proteins called light chains, made by certain cells in the bone marrow, build up in different parts of the body, especially the kidneys. This buildup can make it hard for the kidneys to work properly. The main goal of treatment is to reduce the number of these abnormal proteins by targeting the cells that make them.<br><br>### Common Medications Used for Light Chain Deposition Disease:<br><br>1. **Bortezomib (Velcade)**<br>- **What It Does**: Bortezomib stops certain cells in the bone marrow from getting rid of damaged proteins, causing these cells to die. This lowers the amount

damaged proteins in the cell. Inhibiting this process leads to an accumulation of proteins within the plasma cells, causing them to die. This reduces the production of abnormal light chains.
  - **Potential Side Effects**: Peripheral neuropathy (nerve damage causing tingling or numbness), fatigue, nausea, diarrhea, low blood counts, and an increased risk of infection.

2. **Melphalan**
  - **Mechanism**: Melphalan is an alkylating agent used in chemotherapy that works by cross-linking the DNA strands in cells, thereby inhibiting DNA replication and RNA transcription. This action prevents the proliferation of plasma cells that produce the abnormal proteins.
  - **Potential Side Effects**: Bone marrow suppression (leading to decreased blood cell counts), increased risk of infections, nausea, vomiting, and potential for secondary cancers.

3. **Dexamethasone**
  - **Mechanism**: Dexamethasone is a corticosteroid used to reduce inflammation and suppress the immune system. It acts by decreasing the production of inflammatory chemicals and can induce apoptosis (programmed cell death) in certain immune cells, including plasma cells.
  - **Potential Side Effects**: Increased blood sugar levels, increased risk of infections, weight gain, mood changes, and with long-term use, osteoporosis and muscle weakness.

Additional Treatments:

- **Autologous Stem Cell Transplantation**: For eligible patients, high-dose chemotherapy followed by a stem cell transplant can be an effective treatment. This procedure involves using high doses of chemotherapy to destroy the diseased bone marrow and then replacing it with the patient's own healthy stem cells to rebuild the bone marrow.
- **Cyclophosphamide**: Another chemotherapy agent that can be used in combination with other treatments. It also works by damaging DNA in cells, particularly targeting rapidly dividing cells like plasma cells.
- **Lenalidomide or Thalidomide**: These are immunomodulatory drugs that can enhance the immune response against abnormal plasma cells and inhibit their growth. They are particularly used when patients are refractory or have relapsed after initial therapy.
  - **Potential Side Effects**: Risk of blood clots, birth defects (if used during pregnancy), fatigue, rash, and neutropenia (low white blood cell count).

Monitoring and Management:

Patients with Light Chain Deposition Disease require regular monitoring to manage the disease and monitor for side effects of treatment. This includes routine checks on kidney function, blood tests to monitor blood cell counts, and assessments for signs of organ damage or other complications. Adjustments in therapy may be necessary based on the patient’s response and any side effects encountered.

Conclusion:

Treatment for LCDD involves a combination of therapies aimed at reducing the production of abnormal light chains by destroying or suppressing the plasma cells that produce them. Due to the potent nature of these medications and their potential side effects, close monitoring by healthcare providers is crucial to ensure effective management and to adjust treatment as necessary.

of abnormal proteins being made.
  - **Possible Side Effects**: It can cause nerve damage which might feel like tingling or numbness, make you very tired, cause nausea or diarrhea, lower your blood counts (which makes infections more likely), and make you more likely to get sick.

2. **Melphalan**
  - **What It Does**: Melphalan is a type of chemotherapy that stops cells from copying their DNA, preventing them from multiplying. This is especially effective against cells that produce the abnormal proteins.
  - **Possible Side Effects**: It can lower the number of blood cells, which increases the risk of infections, make you feel sick to your stomach, and might cause vomiting. There’s also a small chance it could lead to other types of cancer later on.

3. **Dexamethasone**
  - **What It Does**: Dexamethasone is a strong medicine that reduces swelling and calms down the immune system to prevent it from causing harm to the body.
  - **Possible Side Effects**: It can raise your blood sugar, increase your chance of getting infections, make you gain weight, change your mood, and, if used for a long time, weaken your bones and muscles.

Additional Treatments:

- **Autologous Stem Cell Transplantation**: For some patients, doctors use strong medicine to destroy all the sick bone marrow and then replace it with the patient's own healthy stem cells. This can sometimes cure the disease.
- **Cyclophosphamide**: Another chemotherapy drug that kills fast-growing cells, including the abnormal plasma cells.
- **Lenalidomide or Thalidomide**: These medicines help boost the immune system to fight against the abnormal cells and stop them from growing.
  - **Possible Side Effects**: There’s a risk of blood clots, birth defects if used during pregnancy, feeling very tired, getting skin rashes, and having lower than normal white blood cell counts which can make it easier to catch infections.

Keeping Track of Your Health:

If you have LCDD, you need regular check-ups to see how your kidneys are doing and how well the treatments are working. This includes tests to check your kidney function and blood tests to keep an eye on the levels of proteins and immune cells. Your doctor will also watch for any side effects from the medicines and might change your treatment if needed.

To Sum It Up:

Treating LCDD involves using medicines to reduce the number of cells making the harmful proteins. Since these treatments can be very strong and have side effects, it’s really important to have regular doctor visits to make sure everything is going as planned and to adjust treatments as needed.

41	Heavy Chain Deposition Disease	Heavy Chain Deposition Disease (HCDD) is a rare condition characterized by the deposition of abnormal heavy chain proteins in the kidneys and sometimes other organs. These proteins are produced by abnormal plasma cells in the bone marrow. Treatment focuses on reducing the production of these abnormal proteins by targeting the plasma cells and managing symptoms to prevent further organ damage.<br><br>### Common Medications Used for Heavy Chain Deposition Disease:<br><br>1. **Bortezomib (Velcade)**<br>- **Mechanism**: Bortezomib is a proteasome inhibitor that blocks the proteasome in plasma cells. The proteasome is involved in degrading unneeded or damaged proteins in the cell. By inhibiting this process, bortezomib causes an accumulation of proteins within the plasma cells, leading to cell stress and death. This reduces the production of abnormal heavy chains.<br>- **Potential Side Effects**: Peripheral neuropathy (nerve damage causing tingling or numbness), fatigue, nausea, diarrhea, low blood counts, and increased risk of infection.<br><br>2. **Melphalan**<br>- **Mechanism**: Melphalan is a chemotherapy agent that works by cross-linking the DNA strands in cells, thereby inhibiting DNA replication and RNA transcription. This action prevents the proliferation of plasma cells that produce the abnormal proteins.<br>- **Potential Side Effects**: Bone marrow suppression (leading to decreased blood cell counts), increased risk of infections, nausea, vomiting, and potential for secondary cancers.<br><br>3. **Dexamethasone**<br>- **Mechanism**: Dexamethasone is a corticosteroid used to reduce inflammation and suppress the immune system. It decreases the production of inflammatory chemicals and can induce apoptosis (programmed cell death) in certain immune cells, including plasma cells.<br>- **Potential Side Effects**: Increased blood sugar levels, increased risk of infections, weight gain, mood changes, and with long-term use, osteoporosis and muscle weakness.<br><br>### Additional Treatments:<br><br>- **Cyclophosphamide**: Another alkylating chemotherapy agent that can be used in combination with other treatments. It also works by damaging DNA in cells, particularly targeting rapidly dividing cells like plasma cells.<br>- **Potential Side Effects**: Bone marrow suppression, increased risk of infections, and potential bladder toxicity.<br><br>- **Autologous Stem Cell Transplantation**: In suitable patients, high-dose chemotherapy followed by a stem cell transplant can be an effective treatment. This procedure involves using high doses of chemotherapy to destroy the diseased bone marrow and then replacing it with the patient's own healthy stem cells to rebuild the bone marrow.<br>- **Potential Side Effects**: High risk of infections, effects from high-dose chemotherapy, and complications related to stem cell transplantation.<br><br>### Monitoring and Management:<br><br>Patients with Heavy Chain Deposition Disease require regular monitoring to assess the effectiveness of the treatment and manage any side effects. This includes routine checks on kidney function, blood tests to monitor blood cell counts, and assessments for signs of organ damage or other complications. Therapy may need adjustments based on the patient’s	Heavy Chain Deposition Disease (HCDD) is a rare condition where abnormal proteins called heavy chains build up in the kidneys and sometimes in other parts of the body. These proteins are made by abnormal plasma cells in the bone marrow. The treatment focuses on reducing the amount of these abnormal proteins by targeting the plasma cells and managing symptoms to prevent further damage.<br><br>### Common Medications Used for Heavy Chain Deposition Disease:<br><br>1. **Bortezomib (Velcade)**<br>- **What It Does**: Bortezomib works by stopping certain cells in the bone marrow from getting rid of damaged proteins. This causes the cells to die, which helps reduce the production of the abnormal heavy chains.<br>- **Possible Side Effects**: It can cause nerve damage that might feel like tingling or numbness, make you feel very tired, cause nausea or diarrhea, lower your blood counts (which makes infections more likely), and increase the risk of getting sick.<br><br>2. **Melphalan**<br>- **What It Does**: Melphalan is a type of chemotherapy that stops cells from copying their DNA, which prevents them from multiplying. This is especially effective against cells that produce abnormal proteins.<br>- **Possible Side Effects**: It can lower the number of blood cells, increasing the risk of infections, make you feel sick to your stomach, and might cause vomiting. There’s also a small chance it could lead to other types of cancer later on.<br><br>3. **Dexamethasone**<br>- **What It Does**: Dexamethasone is a strong medicine that reduces swelling and calms down the immune system to prevent it from causing harm to the body.<br>- **Possible Side Effects**: It can raise your blood sugar, increase your chance of getting infections, make you gain weight, change your mood, and, if used for a long time, weaken your bones and muscles.<br><br>### Additional Treatments:<br><br>- **Cyclophosphamide**: Another chemotherapy drug that kills fast-growing cells, including the abnormal plasma cells.<br>- **Possible Side Effects**: It can lower blood cell counts, increasing the risk of infections, and may cause problems in the bladder.<br><br>- **Autologous Stem Cell Transplantation**: For some patients, doctors use strong medicine to destroy the sick bone marrow and then replace it with the patient's own healthy stem cells. This can sometimes help manage the disease.<br>- **Possible Side Effects**: High risk of infections, effects from strong chemotherapy, and complications from the transplant process.<br><br>### Keeping Track of Your Health:<br><br>If you have HCDD, you need regular check-ups to see how your kidneys and other organs are doing and how well the treatments are working. This includes tests to check your kidney function and blood tests to monitor your blood cell levels and organ health. Your doctor will also watch for any side effects from the medicines and might change your treatment if needed.

		response and any side effects encountered.<br><br>### Conclusion:<br><br>Treatment for Heavy Chain Deposition Disease involves a combination of therapies aimed at reducing the production of abnormal heavy chains by destroying or suppressing the plasma cells that produce them. Due to the potent nature of these medications and their potential side effects, close monitoring by healthcare providers is essential to ensure effective management and to adjust treatment as necessary.	### To Sum It Up:<br><br>Treating HCDD involves using medicines to reduce the number of cells making the harmful proteins. Since these treatments can be very strong and have side effects, it's really important to have regular doctor visits to make sure everything is going as planned and to adjust treatments as needed.
42	Light and Heavy Chain Deposition Disease	Light Chain Deposition Disease (LCDD) and Heavy Chain Deposition Disease (HCDD) are rare conditions characterized by the deposition of abnormal light or heavy immunoglobulin chains in tissues, leading to organ dysfunction, most notably in the kidneys. Treatment strategies focus primarily on reducing the production of abnormal immunoglobulins and managing the symptoms and complications associated with the deposits.<br><br>### Medications Commonly Used in Treatment:<br><br>1. **Bortezomib**:<br>- **Mechanism of Action**: Bortezomib is a proteasome inhibitor that blocks the degradation of ubiquitinated proteins, causing a buildup of these proteins in plasma cells, leading to their apoptosis. It is particularly effective in treating diseases involving abnormal plasma cells that produce monoclonal light chains.<br>- **Potential Side Effects**: Peripheral neuropathy, thrombocytopenia, neutropenia, gastrointestinal disturbances, and increased risk of viral infections.<br><br>2. **Cyclophosphamide**:<br>- **Mechanism of Action**: Cyclophosphamide is a cytotoxic alkylating agent that interferes with the replication and function of DNA, leading to the reduction of abnormal plasma cells.<br>- **Potential Side Effects**: Myelosuppression, increased risk of infections, hemorrhagic cystitis, secondary malignancies, and alopecia.<br><br>3. **Dexamethasone**:<br>- **Mechanism of Action**: Dexamethasone is a corticosteroid used for its potent anti-inflammatory and immunosuppressive effects. It reduces inflammation and works in conjunction with other chemotherapeutic agents to suppress plasma cell proliferation.<br>- **Potential Side Effects**: Hyperglycemia, increased risk of infection, osteoporosis, muscle weakness, and psychiatric effects such as mood swings.<br><br>4. **Melphalan**:<br>- **Mechanism of Action**: Melphalan is another alkylating agent that works similarly to cyclophosphamide, causing cross-linking of DNA strands, ultimately leading to cell death, particularly of rapidly dividing cells like abnormal plasma cells.<br>- **Potential Side Effects**: Bone marrow suppression, gastrointestinal disturbances, mucositis, and secondary malignancies.<br><br>5. **Lenalidomide**:<br>- **Mechanism of Action**: Lenalidomide is an immunomodulatory drug that enhances the immune system's ability to attack abnormal plasma cells, inhibits angiogenesis, and induces cell cycle arrest and apoptosis of neoplastic cells.<br>- **Potential Side Effects**: Thrombosis, neutropenia, increased risk of infections, and rash.<br><br>### Considerations:	Light Chain Deposition Disease (LCDD) and Heavy Chain Deposition Disease (HCDD) are rare conditions where abnormal protein chains build up in the body, often hurting the kidneys. Doctors use special medicines to stop these proteins from forming and help with symptoms.<br><br>### Medications Commonly Used in Treatment:<br><br>1. **Bortezomib**:<br>- **What It Does**: This medicine stops harmful cells that make too many bad proteins from working correctly, causing them to die off.<br>- **Possible Side Effects**: Tingling or numbness in hands and feet, easy bruising, lower immune defense making it easier to catch colds, stomach upset.<br><br>2. **Cyclophosphamide**:<br>- **What It Does**: This medicine messes up the DNA in bad cells, which stops them from growing and makes them die.<br>- **Possible Side Effects**: Lower immune defense, easier to get infections, bladder irritation, hair loss, and a slight chance of other serious illnesses later on.<br><br>3. **Dexamethasone**:<br>- **What It Does**: This is a strong anti-swelling medicine that helps reduce inflammation and also helps other cancer-fighting medicines work better.<br>- **Possible Side Effects**: Higher blood sugar levels, more infections, weaker bones, muscle weakness, mood changes.<br><br>4. **Melphalan**:<br>- **What It Does**: Like cyclophosphamide, it damages the DNA in bad cells, causing them to stop growing and die.<br>- **Possible Side Effects**: Lower immune defense, upset stomach, mouth sores, and a slight chance of other serious illnesses later on.<br><br>5. **Lenalidomide**:<br>- **What It Does**: This medicine boosts the body's defense system to better fight off the bad cells and stops harmful cells from getting the blood supply they need to grow.<br>- **Possible Side Effects**: Blood clots, lower immune defense, infections, skin rash.<br><br>### What to Think About:<br>Doctors pick the medicine based on how healthy someone is, how badly their organs are affected, and if they have any other conditions. Sometimes, people might also need treatment like dialysis if their kidneys are really affected.

		The choice of treatment is typically guided by the overall health of the patient, the severity of organ involvement, and the presence of any underlying plasma cell dyscrasia such as multiple myeloma. In addition to these medications, supportive care, including renal replacement therapy, may be necessary for patients with significant kidney involvement.<br><br>### Advanced Therapies:<br>Given your interest in cutting-edge technologies, it's also worth mentioning that research into newer monoclonal antibodies and other targeted therapies is ongoing. These therapies aim to provide more specific targeting of abnormal cells with potentially fewer side effects.<br><br>This disease's rarity and complexity often require a multidisciplinary approach for effective management, involving nephrologists, hematologists, and potentially other specialists. Each patient's treatment plan is highly personalized based on their specific clinical scenario.	These diseases are very rare and need doctors from different areas to work together to provide the best care. The treatment plan is made just for each person based on their unique health needs.
43	Crystalglobulin GN	Crystalglobulinemia glomerulonephritis (Crystalglobulin GN) is a rare disease characterized by the deposition of monoclonal immunoglobulins that form crystalline structures in the kidneys, often leading to kidney damage. The treatment aims to reduce the production of these abnormal immunoglobulins and manage the resulting kidney dysfunction.<br><br>### Medications Commonly Used in Treatment:<br><br>1. **Bortezomib**:<br>- **Mechanism of Action**: Bortezomib is a proteasome inhibitor that promotes the death of plasma cells, which are the cells producing the abnormal immunoglobulins. By inhibiting the proteasome, it prevents the breakdown of proteins, leading to the accumulation of excess proteins inside the cells, which causes cell death.<br>- **Potential Side Effects**: Peripheral neuropathy (tingling or numbness in the extremities), thrombocytopenia (low platelet count), neutropenia (low white blood cell count), gastrointestinal problems (nausea, diarrhea), and an increased risk of viral infections.<br><br>2. **Cyclophosphamide**:<br>- **Mechanism of Action**: Cyclophosphamide is an alkylating agent that interferes with DNA replication in cells, effectively reducing the number of plasma cells producing the abnormal immunoglobulins.<br>- **Potential Side Effects**: Myelosuppression (suppression of bone marrow activity), increased risk of infections, hemorrhagic cystitis (bladder inflammation with bleeding), secondary malignancies, and alopecia (hair loss).<br><br>3. **Dexamethasone**:<br>- **Mechanism of Action**: Dexamethasone is a corticosteroid with potent anti-inflammatory and immunosuppressive properties. It reduces inflammation and aids in suppressing the immune system's activity, thereby decreasing the production of abnormal proteins.<br>- **Potential Side Effects**: Increased blood sugar, higher risk of infections, osteoporosis (bone weakening), muscle weakness, and mood swings.<br><br>4. **Rituximab**:<br>- **Mechanism of Action**: Rituximab is a monoclonal antibody that specifically targets CD20, a protein found on the surface of B cells (including abnormal plasma cells). This binding induces the destruction of these cells, reducing the production of the harmful immunoglobulins.<br>- **Potential Side Effects**: Infusion reactions (fever, chills, rash), increased risk of infections, and potential for severe skin and mouth reactions.	Crystalglobulinemia glomerulonephritis (Crystalglobulin GN) is a rare kidney disease caused by unusual protein crystals building up in the kidneys. This can harm the kidneys over time. Doctors use special medicines to stop these proteins from forming and to help keep the kidneys working well.<br><br>### Medications Commonly Used in Treatment:<br><br>1. **Bortezomib**:<br>- **What It Does**: This medicine stops harmful cells that make too many bad proteins from working right, causing them to die.<br>- **Possible Side Effects**: Tingling or numbness in hands and feet, easy bruising, getting colds more often, stomach upset like nausea or diarrhea.<br><br>2. **Cyclophosphamide**:<br>- **What It Does**: This medicine messes up the DNA in bad cells, which stops them from growing and makes them die.<br>- **Possible Side Effects**: Lower immune defense, easier to get infections, bladder irritation with bleeding, hair loss, and a slight chance of other serious illnesses later on.<br><br>3. **Dexamethasone**:<br>- **What It Does**: This is a strong anti-swelling medicine that helps reduce inflammation and also helps other cancer-fighting medicines work better.<br>- **Possible Side Effects**: Higher blood sugar levels, more infections, weaker bones, muscle weakness, mood changes.<br><br>4. **Rituximab**:<br>- **What It Does**: This medicine targets specific bad cells that are part of the problem, helping to destroy them and reduce the amount of harmful proteins.<br>- **Possible Side Effects**: Reactions during the medicine infusion like fever or rash, more infections, and sometimes severe skin reactions.<br><br>### What to Think About:<br>Doctors choose the right medicine based on how sick someone is and how much the kidneys are affected. They might also use treatments like dialysis, which is a machine that helps clean the blood if the kidneys aren't working well.<br><br>These diseases are very rare and need doctors from different areas to work together

		### Considerations and Additional Treatments:<br>Treatment strategies are tailored based on the specific characteristics of the disease in each patient, including the severity of kidney damage and any underlying conditions like multiple myeloma. In addition to these medications, supportive care measures are crucial, especially focusing on preserving kidney function. This might include interventions such as dialysis in cases where kidney function is severely compromised.<br><br>### Advanced and Investigational Therapies:<br>Emerging treatments, such as newer monoclonal antibodies and proteasome inhibitors, are under investigation and may offer more targeted approaches with potentially fewer side effects. These advanced therapies are particularly important to consider given the rarity and complexity of the disease, where traditional treatment methods may not be fully effective.<br><br>Overall, the management of Crystalglobulin GN requires a comprehensive approach that includes a combination of pharmacological treatment, monitoring of kidney function, and potentially, intervention to address complications such as kidney failure.	to provide the best care. The treatment plan is made just for each person based on their unique health needs.
44	Monotypic Anti-GBM Disease	Monotypic Anti-Glomerular Basement Membrane (Anti-GBM) Disease is a rare autoimmune disorder where the body's immune system mistakenly attacks and damages the kidney's filtering units, the glomeruli, and sometimes the lungs. This condition is characterized by the production of antibodies that specifically bind to the glomerular basement membrane. The treatment focuses on stopping these harmful antibodies from causing more damage.<br><br>### Medications Commonly Used in Treatment:<br><br>1. **Corticosteroids (e.g., Prednisone)**:<br>- **Mechanism of Action**: Corticosteroids are powerful anti-inflammatory drugs that reduce inflammation and suppress the immune system's overall activity, helping to decrease the production of harmful antibodies.<br>- **Potential Side Effects**: Increased risk of infections, elevated blood sugar levels, weight gain, increased appetite, mood swings, insomnia, and long-term use can lead to bone thinning (osteoporosis).<br><br>2. **Cyclophosphamide**:<br>- **Mechanism of Action**: Cyclophosphamide is a cytotoxic drug that suppresses the immune system by damaging the DNA of immune cells, particularly those responsible for the autoimmune attack, thus reducing antibody production.<br>- **Potential Side Effects**: Myelosuppression (lowering the blood cell count), increased susceptibility to infections, potential bladder toxicity (hemorrhagic cystitis), and a risk of secondary cancers.<br><br>3. **Rituximab**:<br>- **Mechanism of Action**: Rituximab is a monoclonal antibody that targets CD20 on B lymphocytes, which are a type of white blood cell involved in the immune response, including the production of antibodies. By depleting these cells, Rituximab can reduce the levels of harmful antibodies in the blood.<br>- **Potential Side Effects**: Infusion reactions (fever, chills, rash), increased risk of infections, and rarely, severe skin and mouth reactions.<br><br>4. **Plasmapheresis (Plasma Exchange)**:<br>- **Mechanism of Action**: Not a medication but a procedure where blood is filtered to remove harmful antibodies from the plasma (the liquid part of the blood), thereby reducing	Monotypic Anti-GBM Disease is a rare illness where the body's defense system mistakenly attacks the kidneys and sometimes the lungs. This happens because the body makes harmful antibodies that damage the kidneys' filtering parts. Doctors use special treatments to stop these harmful antibodies and help the body heal.<br><br>### Medications Commonly Used in Treatment:<br><br>1. **Corticosteroids (like Prednisone)**:<br>- **What It Does**: These are strong medicines that reduce swelling and lower the activity of the body's defense system to help stop it from harming the kidneys.<br>- **Possible Side Effects**: More chances of getting sick, higher blood sugar, gaining weight, feeling hungrier, mood changes, trouble sleeping, and weaker bones if used for a long time.<br><br>2. **Cyclophosphamide**:<br>- **What It Does**: This medicine calms the immune system by damaging the DNA of certain cells, reducing the number of bad antibodies that attack the kidneys.<br>- **Possible Side Effects**: Lower number of blood cells which can increase infection risk, bladder irritation, and a slight chance of other serious illnesses later on.<br><br>3. **Rituximab**:<br>- **What It Does**: This medicine targets specific cells in the immune system that help produce the harmful antibodies, lowering the amount of these bad antibodies.<br>- **Possible Side Effects**: Reactions during the treatment like fever or rash, more infections, and sometimes severe skin reactions.<br><br>4. **Plasmapheresis (Plasma Exchange)**:<br>- **What It Does**: This is not a medicine but a special procedure where doctors filter the blood to remove the harmful antibodies. This can quickly help reduce damage to the kidneys and lungs.<br>- **Possible Side Effects**: Lower blood pressure during the procedure, bleeding from the treatment, and possible allergic reactions to the fluids used in the process.<br><br>### What to Think About:

		the immediate damage to the kidneys and lungs.<br>- **Potential Side Effects**: Low blood pressure, bleeding from anticoagulants used during the procedure, and potential for allergic reactions to the plasma substitutes.<br><br>### Considerations:<br>The choice and combination of these treatments depend significantly on the severity of the symptoms and the extent of organ involvement. Rapid intervention is crucial to prevent irreversible damage to the kidneys or lungs. Following the acute phase of treatment, maintenance therapy with less aggressive medications might be necessary to prevent relapse.<br><br>### Emerging Treatments:<br>Research is ongoing into more specific treatments that can target the immune response more precisely, potentially leading to fewer side effects and better overall outcomes. These include newer biologic drugs that specifically inhibit the pathways involved in the production of harmful antibodies.<br><br>In managing Monotypic Anti-GBM Disease, a multidisciplinary approach is typically required, involving nephrologists, rheumatologists, and sometimes pulmonologists, to address the complex challenges of this condition and to tailor the treatment to the individual needs of the patient.	Doctors choose the best treatment based on how serious the illness is and which organs are affected. Starting treatment quickly is important to prevent lasting damage to the kidneys or lungs. After the main treatment, less strong medicines might be needed to keep the illness from coming back.<br><br>### New Research:<br>Doctors and scientists are working on new treatments that might work even better and have fewer side effects. These new treatments try to more precisely target the parts of the immune system that cause the problem.<br><br>Treating Monotypic Anti-GBM Disease often needs many different doctors working together to take care of the patient, including kidney specialists, arthritis and immune system doctors, and sometimes lung doctors, to make sure the treatment is just right for what each patient needs.
45	Monotypic Membranous Nephropathy	Monotypic Membranous Nephropathy is a subtype of membranous nephropathy characterized by the deposition of monoclonal immunoglobulin G (IgG) on the kidney's glomerular basement membrane, leading to kidney damage and proteinuria (excessive protein in urine). The treatment strategies aim to reduce the immune response causing these deposits and preserve kidney function.<br><br>### Medications Commonly Used in Treatment:<br><br>1. **Rituximab**:<br>- **Mechanism of Action**: Rituximab is a monoclonal antibody that targets CD20, a protein found on the surface of B-cells (a type of white blood cell). By binding to CD20, Rituximab leads to the destruction of these cells, which are responsible for producing the harmful antibodies contributing to kidney damage.<br>- **Potential Side Effects**: Infusion reactions (fever, chills, rash), increased risk of infections, and rarely, severe skin and mouth reactions.<br><br>2. **Corticosteroids (e.g., Prednisone)**:<br>- **Mechanism of Action**: Corticosteroids are anti-inflammatory drugs that also suppress the immune system’s overall activity. This helps reduce inflammation and decrease the production of antibodies attacking the kidney.<br>- **Potential Side Effects**: Higher risk of infections, increased blood sugar levels, weight gain, mood swings, increased appetite, insomnia, and with long-term use, osteoporosis.<br><br>3. **Cyclophosphamide**:<br>- **Mechanism of Action**: Cyclophosphamide is a cytotoxic drug that suppresses the immune system by interfering with DNA replication in cells, particularly immune cells. This reduces the proliferation of cells producing harmful antibodies.<br>- **Potential Side Effects**: Myelosuppression (decreased blood cell production), increased risk of infections, possible bladder irritation (hemorrhagic cystitis), and a risk of secondary cancers.<br><br>4. **Calcineurin Inhibitors (e.g., Tacrolimus, Cyclosporine)**:	Monotypic Membranous Nephropathy is a kind of kidney disease where harmful protein builds up in the kidneys, making it hard for them to work properly. The goal of treatment is to calm down the body’s immune system that’s mistakenly attacking the kidneys and to keep the kidneys working well.<br><br>### Medications Commonly Used in Treatment:<br><br>1. **Rituximab**:<br>- **What It Does**: This medicine targets specific cells in the immune system that are making the harmful proteins. By stopping these cells, it helps protect the kidneys from further damage.<br>- **Possible Side Effects**: Reactions during the medicine infusion like fever or rash, more infections, and sometimes severe skin reactions.<br><br>2. **Corticosteroids (like Prednisone)**:<br>- **What It Does**: These are strong medicines that reduce swelling and lower the activity of the body’s defense system to help stop it from harming the kidneys.<br>- **Possible Side Effects**: More chances of getting sick, higher blood sugar, gaining weight, mood changes, feeling hungrier, trouble sleeping, and weaker bones if used for a long time.<br><br>3. **Cyclophosphamide**:<br>- **What It Does**: This medicine calms the immune system by damaging the DNA of certain cells, reducing the number of bad antibodies that attack the kidneys.<br>- **Possible Side Effects**: Lower number of blood cells which can increase infection risk, bladder irritation, and a slight chance of other serious illnesses later on.<br><br>4. **Calcineurin Inhibitors (like Tacrolimus or Cyclosporine)**:<br>- **What It Does**: These medicines help lower the activity of certain immune cells, reducing the damage to the kidneys.<br>- **Possible Side Effects**: Kidney problems (which need careful monitoring),

		- **Mechanism of Action**: Calcineurin inhibitors reduce immune activity by inhibiting calcineurin, a protein important for activating T-cells, another type of immune cell involved in the autoimmune process.<br>- **Potential Side Effects**: Kidney toxicity (particularly important to monitor given the pre-existing kidney condition), increased risk of infections, tremors, high blood pressure, and potential for diabetes.<br><br>### Considerations:<br>The choice of therapy often depends on the severity of the symptoms, the extent of protein loss in the urine, and how quickly the kidney function is deteriorating. Treatment is usually aimed at controlling the symptoms and slowing down the progression of the disease. In severe cases, combinations of these medications may be used to achieve a more effective suppression of the immune system.<br><br>### Monitoring and Adjustments:<br>Given the potential for significant side effects, especially with treatments that heavily suppress the immune system, close monitoring by healthcare providers is crucial. Adjustments to medication doses or changes in treatment strategies are often needed based on the patient’s response and any side effects they experience.<br><br>### Emerging Treatments:<br>Research into more targeted therapies continues, with the aim of reducing side effects and improving outcomes for patients with Monotypic Membranous Nephropathy. New biologic agents and molecularly targeted drugs are being investigated, which may offer more precise mechanisms of action with potentially fewer side effects.<br><br>Treatment of Monotypic Membranous Nephropathy typically involves a collaborative approach between nephrologists and possibly other specialists to tailor the best possible management strategy for each patient.	more infections, shaky hands, high blood pressure, and a chance of getting diabetes.<br><br>### What to Think About:<br>Doctors decide on the best treatment based on how serious the disease is and how it's affecting the kidneys. Sometimes, they might use more than one medicine together to get better results.<br><br>### Checking How Treatment Is Going:<br>Because the medicines can cause other health problems, doctors will keep a close eye on how well the treatment is working and how the body is handling the medicines. They might adjust the doses or change medicines based on what they see.<br><br>### New Research:<br>Scientists are always looking for new and better ways to treat this kidney disease with fewer side effects. They are testing new medicines that specifically target the parts of the immune system causing the problem.<br><br>Treating this kidney disease usually involves working with kidney specialists and sometimes other doctors to make sure the treatment is just right for what each patient needs.
46	Thrombotic Microangiopathies	Thrombotic Microangiopathies (TMAs) refer to a group of disorders characterized by small blood clots that form in the small blood vessels, leading to reduced blood flow and potential damage to organs such as the kidneys and brain. Common types of TMA include thrombotic thrombocytopenic purpura (TTP) and atypical hemolytic uremic syndrome (aHUS). Treatment strategies are primarily aimed at managing clot formation and supporting the affected organs.<br><br>### Medications Commonly Used in Treatment:<br><br>1. **Plasma Exchange (Plasmapheresis)**:<br>- **Mechanism of Action**: Although not a medication, plasma exchange is a procedure where the plasma (the liquid part of blood) is removed and replaced with donor plasma or a plasma substitute. This helps remove antibodies or other substances causing the clotting.<br>- **Potential Side Effects**: Low blood pressure, bleeding from anticoagulants used during the procedure, and potential for allergic reactions to the plasma substitutes.<br><br>2. **Eculizumab**:<br>- **Mechanism of Action**: Eculizumab is a monoclonal antibody that inhibits the complement system, which is part of the immune system that can become overly active in conditions like aHUS, leading to blood clot formation.<br>- **Potential Side Effects**: Increased risk of infections, particularly meningococcal infections, which requires patients to be vaccinated against these infections before starting treatment. Headache and nausea are also common.	Thrombotic Microangiopathies (TMAs) are a group of serious conditions where tiny blood clots form in small blood vessels, which can block blood flow and harm organs like the kidneys and brain. There are different types of TMAs, including ones called thrombotic thrombocytopenic purpura (TTP) and atypical hemolytic uremic syndrome (aHUS). The main goal of treatment is to stop these clots from forming and to help the organs work properly.<br><br>### Medications Commonly Used in Treatment:<br><br>1. **Plasma Exchange (Plasmapheresis)**:<br>- **What It Does**: This isn't a medicine but a special procedure where doctors replace the liquid part of your blood (plasma) with new plasma or a substitute. This helps remove harmful substances that cause clotting.<br>- **Possible Side Effects**: Feeling dizzy, bleeding from the place where the needle goes in, and allergies to the new plasma.<br><br>2. **Eculizumab**:<br>- **What It Does**: This medicine stops part of the body's defense system from working too hard, which can cause the clots. This helps prevent new clots from forming.<br>- **Possible Side Effects**: More chances of getting infections (especially a serious type called meningococcal infection), headaches, and feeling sick to your stomach.

		3. **Caplacizumab**:<br>- **Mechanism of Action**: Caplacizumab is a monoclonal antibody that targets von Willebrand factor (a protein involved in clotting), preventing it from interacting with platelets and thereby reducing clot formation. This is especially used in acute TTP.<br>- **Potential Side Effects**: Bleeding, headache, and minor gastrointestinal symptoms.<br><br>4. **Rituximab**:<br>- **Mechanism of Action**: Rituximab is used particularly in refractory or recurrent cases of TTP. It targets CD20 on B cells, leading to their destruction and reducing the production of autoantibodies that can promote clotting.<br>- **Potential Side Effects**: Infusion reactions, increased risk of infections, and rarely severe skin and mouth reactions.<br><br>5. **Corticosteroids**:<br>- **Mechanism of Action**: Used to suppress the immune system and reduce inflammation, corticosteroids can help decrease the production of antibodies contributing to TMA.<br>- **Potential Side Effects**: Increased risk of infections, high blood sugar levels, increased appetite, weight gain, mood swings, and possible bone loss with long-term use.<br><br>### Considerations:<br>Treatment is often urgent to prevent significant organ damage and manage acute symptoms. The approach depends on the specific type of TMA and the severity of the symptoms. Close monitoring and supportive care are critical, including managing blood pressure and ensuring good kidney function.<br><br>### Advanced and Supportive Therapies:<br>Supportive treatments, such as dialysis, may be necessary for kidney support in severe cases. Research into more specific and less invasive treatments continues, aiming to improve outcomes and reduce side effects for patients with TMA.<br><br>Managing TMA requires a multidisciplinary approach, often involving hematologists, nephrologists, and other specialists to address the complex and potentially life-threatening nature of these disorders.	3. **Caplacizumab**:<br>- **What It Does**: This medicine targets a specific protein that helps clots form. By stopping this protein, it helps reduce the number of clots.<br>- **Possible Side Effects**: Easier bleeding, headaches, and stomach problems like nausea.<br><br>4. **Rituximab**:<br>- **What It Does**: This is used mostly when other treatments don’t work well. It targets certain cells that make harmful proteins, reducing their numbers and helping to stop clot formation.<br>- **Possible Side Effects**: Reactions during the treatment like fever or chills, more infections, and sometimes severe skin reactions.<br><br>5. **Corticosteroids**:<br>- **What It Does**: These medicines help calm down the immune system and reduce swelling, which can lower the number of harmful proteins that cause clotting.<br>- **Possible Side Effects**: More chances of getting infections, higher blood sugar, gaining weight, mood changes, feeling hungrier, and trouble sleeping.<br><br>### What to Think About:<br>Doctors need to treat these conditions quickly to avoid serious damage to the organs. They pick the best treatment based on the type of TMA and how severe it is. Watching the patient closely and managing other health problems like high blood pressure and kidney function is also important.<br><br>### Extra Help:<br>Sometimes, if the kidneys are really hurt, treatments like dialysis (a machine that helps clean the blood) might be needed.<br><br>Doctors, including blood specialists and kidney specialists, work together to provide the best care for people with TMA, trying to improve their health with as few side effects as possible.
47	Thrombotic Thrombocytopenic Purpura	Thrombotic Thrombocytopenic Purpura (TTP) is a rare blood disorder characterized by the formation of tiny blood clots throughout the body, leading to low platelet counts, anemia, kidney malfunction, fever, and neurological symptoms. Effective treatment is crucial for managing this life-threatening condition.<br><br>### Medications Commonly Used in Treatment:<br><br>1. **Plasma Exchange (Plasmapheresis)**:<br>- **Mechanism of Action**: Plasma exchange involves removing the patient’s blood plasma (which contains the abnormal proteins causing clot formation) and replacing it with fresh plasma or a plasma substitute. This procedure rapidly removes the antibodies or enzymes responsible for the blood clots.<br>- **Potential Side Effects**: Low blood pressure, bleeding due to anticoagulants used during the procedure, risk of infection, and possible allergic reactions to the plasma substitutes.<br><br>2. **Corticosteroids (e.g., Prednisone)**:<br>- **Mechanism of Action**: Corticosteroids are used to suppress the immune system and reduce inflammation. This helps decrease the production of antibodies that contribute to clot formation and blood vessel damage.	Thrombotic Thrombocytopenic Purpura (TTP) is a very serious blood disorder where small blood clots form all over the body. This can cause low platelet counts (which help with clotting), anemia (not enough red blood cells), kidney problems, fever, and brain issues. It's really important to treat this condition quickly to avoid severe health problems.<br><br>### Medications Commonly Used in Treatment:<br><br>1. **Plasma Exchange (Plasmapheresis)**:<br>- **What It Does**: In this treatment, doctors take out the patient’s blood plasma (which has bad proteins causing clots) and replace it with new plasma. This helps remove the bad substances quickly.<br>- **Possible Side Effects**: Feeling dizzy or light-headed, bleeding where the needle goes in, risk of getting sick from the procedure, and allergies to the new plasma.<br><br>2. **Corticosteroids (like Prednisone)**:<br>- **What It Does**: These medicines help calm down the body’s defense system and reduce swelling. This slows down the production of harmful substances that

		- **Potential Side Effects**: Increased susceptibility to infections, high blood sugar, weight gain, mood changes, insomnia, and with prolonged use, bone thinning.<br><br>3. **Rituximab**:<br>- **Mechanism of Action**: Rituximab is a monoclonal antibody that targets CD20 on B-cells. By depleting these cells, Rituximab reduces the levels of harmful antibodies in the bloodstream that are involved in the abnormal clotting process seen in TTP.<br>- **Potential Side Effects**: Infusion-related reactions (like fever, chills, nausea), increased risk of infections, and rarely, severe skin and mouth reactions.<br><br>4. **Caplacizumab**:<br>- **Mechanism of Action**: Caplacizumab targets von Willebrand factor, a protein that helps platelets clump and form clots. By blocking this interaction, it helps reduce the formation of unnecessary clots.<br>- **Potential Side Effects**: Nosebleeds, headache, bleeding gums, and other bleeding-related issues due to the drug's effect on clotting.<br><br>### Additional Considerations:<br>Immediate treatment is crucial in TTP to prevent serious complications such as stroke, heart attack, or severe kidney damage. The treatment strategy may involve a combination of the above medications depending on the severity of the condition and the response to initial therapy.<br><br>### Long-term Management:<br>Following stabilization, patients may require ongoing monitoring and intermittent treatment to prevent relapse, which is common in TTP. Lifestyle adjustments and supportive care are also essential parts of managing the condition long-term.<br><br>In TTP, the goal of treatment is not only to stop the formation of clots but also to prevent further complications and improve the patient's quality of life. Each treatment plan is tailored to the individual's specific needs, often requiring a team of specialists to manage the disease effectively.	lead to clotting.<br>- **Possible Side Effects**: Getting sick more easily, higher blood sugar, gaining weight, mood swings, trouble sleeping, and weaker bones if used for a long time.<br><br>3. **Rituximab**:<br>- **What It Does**: This medicine targets certain cells in the immune system that make bad antibodies causing clots. By reducing these cells, it helps lower the risk of clots.<br>- **Possible Side Effects**: Reactions during the treatment like fever or chills, more infections, and sometimes severe skin reactions.<br><br>4. **Caplacizumab**:<br>- **What It Does**: This medicine works on a specific protein that helps platelets stick together and form clots. By blocking this, it reduces unnecessary clot formation.<br>- **Possible Side Effects**: Nosebleeds, headaches, and bleeding gums, because it affects how blood clots.<br><br>### What to Think About:<br>It's very important to start treatment right away to avoid serious problems like a stroke or heart attack. Depending on how severe the TTP is, doctors might use a combination of these treatments.<br><br>### Long-term Care:<br>After the serious part is under control, doctors keep checking on the patient and might continue some treatments to prevent the TTP from coming back. It's also important for the patient to take care of their health and make some lifestyle changes as recommended by doctors.<br><br>In TTP, treatments aim to stop clots and keep them from causing more harm, while also trying to improve the patient's overall health. Each patient might need different treatments, and often several doctors work together to provide the best care.
48	Hemolytic Uremic Syndrome	Hemolytic Uremic Syndrome (HUS) is a severe condition primarily characterized by the destruction of red blood cells, acute kidney failure, and low platelet count. It most commonly occurs as a result of infection with certain strains of **E. coli** bacteria, which produce toxins (Shiga toxin) that cause damage to the lining of the blood vessels, leading to the symptoms of HUS. Treatment focuses on managing the symptoms and preventing further damage to the kidneys.<br><br>### Medications Commonly Used in Treatment:<br><br>1. **Supportive Care (Fluids and Electrolytes)**:<br>- **Mechanism of Action**: Not a medication, but intravenous fluids and electrolytes are vital for maintaining proper fluid balance and replacing nutrients lost due to kidney damage and diarrhea.<br>- **Potential Side Effects**: Care must be taken to avoid fluid overload, which can lead to swelling and increased blood pressure.<br><br>2. **Eculizumab** (for atypical HUS):<br>- **Mechanism of Action**: Eculizumab is a monoclonal antibody that binds to and inhibits the complement system's protein C5, part of the immune system that becomes overactive in atypical HUS. By inhibiting this protein, it helps prevent the formation of blood clots and	Hemolytic Uremic Syndrome (HUS) is a serious condition where red blood cells break down, kidneys may stop working well, and there are fewer platelets (which help blood clot). It often happens after an infection, like some kinds of bad **E. coli** bacteria, which make toxins that damage blood vessels. The goal of treatment is to help symptoms and protect the kidneys from more damage.<br><br>### Medications Commonly Used in Treatment:<br><br>1. **Supportive Care (Fluids and Electrolytes)**:<br>- **What It Does**: Doctors give fluids through a vein to help keep the body hydrated and replace important nutrients lost during illness. This helps keep everything balanced.<br>- **Possible Side Effects**: Doctors need to be careful not to give too much fluid, which can cause swelling and raise blood pressure.<br><br>2. **Eculizumab** (for a special kind called atypical HUS):<br>- **What It Does**: This medicine stops a part of the body's defense system from overworking, which can prevent blood clots and protect the kidneys.<br>- **Possible Side Effects**: There's a higher chance of getting serious infections like meningitis, so doctors give a vaccine before starting this medicine. It can also

		protects the kidneys from further damage.<br>- **Potential Side Effects**: Increased risk of meningococcal and other infections, headache, nausea, and hypertension. Vaccination against meningococcus is required before starting treatment.<br><br>3. **Dialysis**:<br>- **Mechanism of Action**: In cases where kidney function is significantly impaired, dialysis may be necessary to help remove toxins and excess fluids from the blood, essentially performing the kidney's filtering functions.<br>- **Potential Side Effects**: Low blood pressure, muscle cramps, infection risks associated with dialysis access sites, and long-term complications related to chronic dialysis.<br><br>4. **Plasma Exchange** (for some types of secondary HUS):<br>- **Mechanism of Action**: Plasma exchange involves removing the patient's plasma and replacing it with donor plasma. This can help remove the toxins or antibodies causing the disease, especially in cases not caused by Shiga toxin-producing **E. coli**.<br>- **Potential Side Effects**: Low blood pressure, bleeding due to anticoagulants used during the procedure, risk of infection, and possible allergic reactions to the donor plasma.<br><br>5. **Corticosteroids**:<br>- **Mechanism of Action**: These are used to reduce inflammation and regulate the immune response. In the context of HUS, they might be used to manage symptoms or treat underlying autoimmune conditions that could be contributing to the syndrome.<br>- **Potential Side Effects**: Increased risk of infections, high blood sugar levels, increased appetite, weight gain, mood swings, and with long-term use, bone thinning.<br><br>### Considerations:<br>The treatment approach varies depending on the type of HUS (typical or atypical) and the underlying causes. Typical HUS, often caused by an infectious agent, might not require as intensive immune-modulating therapies as atypical HUS, which is driven by genetic or autoimmune factors.<br><br>### Monitoring and Adjustments:<br>Close monitoring of kidney function, blood pressure, and blood counts is critical in managing HUS. Adjustments to treatment are often necessary based on the patient's response to therapy and progression of the disease.<br><br>In managing HUS, a multidisciplinary approach is typically required, involving nephrologists, hematologists, and other specialists to address the complex and potentially life-threatening nature of this disorder. The goal is to support kidney function while managing the acute symptoms and preventing long-term complications.	cause headaches, nausea, and high blood pressure.<br><br>3. **Dialysis**:<br>- **What It Does**: If the kidneys are really struggling, dialysis helps clean the blood, doing the job of the kidneys.<br>- **Possible Side Effects**: It can cause low blood pressure, muscle cramps, and infections where the dialysis is connected.<br><br>4. **Plasma Exchange** (used sometimes when HUS is caused by other things than **E. coli**):<br>- **What It Does**: This process takes out the patient's plasma (part of the blood) and replaces it with new plasma. This can remove bad substances or antibodies causing the disease.<br>- **Possible Side Effects**: It can lead to low blood pressure, bleeding from blood thinners used during the procedure, risk of infections, and allergic reactions to the new plasma.<br><br>5. **Corticosteroids**:<br>- **What It Does**: These are used to lower inflammation and control the body's immune response. They can help treat symptoms or deal with autoimmune conditions that might be causing HUS.<br>- **Possible Side Effects**: Increased chance of infections, higher blood sugar, gaining weight, mood changes, and weak bones if used for a long time.<br><br>### What to Think About:<br>Doctors choose treatments based on what caused the HUS and how severe it is. For HUS caused by bacteria, the focus is on supporting the body until it heals. For the atypical kind, more specific treatments like Eculizumab might be needed.<br><br>### Keeping an Eye on Things:<br>Doctors closely watch how the treatment is working, especially how well the kidneys are doing and how the blood looks under a microscope. They might need to change the treatment based on how the patient is responding.<br><br>Treating HUS usually involves a team of doctors, including kidney specialists and blood doctors, who work together to give the best care possible. The main goals are to manage the symptoms, support kidney health, and prevent more problems.
49	Secondary Thrombotic Microangiopathies	Secondary Thrombotic Microangiopathies (TMAs) refer to conditions where blood clotting in small vessels is a complication of another underlying condition, such as cancer, autoimmune diseases, infection, or the use of certain medications. Treatment focuses on managing the underlying cause and addressing the blood clotting itself.<br><br>### Medications Commonly Used in Treatment:<br><br>1. **Eculizumab**:<br>- **Mechanism of Action**: Eculizumab is a monoclonal antibody that inhibits the complement system's protein C5, which plays a role in immune responses that can contribute to clotting in TMAs. By blocking this protein, Eculizumab helps reduce clot formation and can	Secondary Thrombotic Microangiopathies (TMAs) are conditions where tiny blood clots form in small blood vessels. These clots can happen because of another health problem like cancer, autoimmune diseases (where the body attacks itself), infections, or certain medications. The goal of treatment is to manage the main cause and help stop the clotting.<br><br>### Medications Commonly Used in Treatment:<br><br>1. **Eculizumab**:<br>- **What It Does**: This medicine stops a part of the body's defense system from working too hard, which can prevent the formation of blood clots and protect

protect the kidneys and other organs from damage.
- **Potential Side Effects**: Increased risk of severe infections, especially meningococcal infections, so vaccination is necessary before starting treatment. Other side effects can include headache, nausea, and hypertension.

2. **Rituximab**:
- **Mechanism of Action**: Rituximab targets CD20 on B cells, leading to their destruction. It is used to reduce the levels of harmful antibodies in autoimmune-related TMAs.
- **Potential Side Effects**: Infusion-related reactions, increased risk of infections, and rarely, severe skin and mouth reactions.

3. **Corticosteroids**:
- **Mechanism of Action**: These drugs suppress the immune system and reduce inflammation, which is helpful in autoimmune-induced TMA or in cases where inflammation contributes to the condition.
- **Potential Side Effects**: Increased risk of infections, high blood sugar levels, increased appetite, weight gain, mood swings, and potential for bone thinning with long-term use.

4. **Immunosuppressants (e.g., Cyclophosphamide, Mycophenolate Mofetil)**:
- **Mechanism of Action**: These medications inhibit immune cell proliferation, reducing the immune system's activity and helping to manage autoimmune diseases that can lead to TMA.
- **Potential Side Effects**: Myelosuppression (decrease in bone marrow activity), increased risk of infections, potential for liver and kidney toxicity, and gastrointestinal issues.

5. **Plasma Exchange**:
- **Mechanism of Action**: Plasma exchange involves removing the patient’s plasma (which may contain harmful antibodies or other factors contributing to TMA) and replacing it with donor plasma or a plasma substitute. This can help quickly reduce the factors causing the clotting.
- **Potential Side Effects**: Risk of bleeding from anticoagulants used during the procedure, low blood pressure, risk of infection, and possible allergic reactions to the plasma substitutes.

Considerations:
The choice of treatment is significantly influenced by the underlying cause of the secondary TMA. For instance, if a medication is the trigger, discontinuing that medication may be the first step. If an infection is involved, treating that infection is critical.

Monitoring and Adjustments:
Patients with secondary TMAs require careful monitoring to assess their response to treatment and adjust as needed. Regular checks of kidney function, blood counts, and other relevant parameters are essential.

Advanced and Supportive Therapies:
Supportive treatments such as blood transfusions for severe anemia or interventions to manage blood pressure and kidney function may also be necessary. The complex nature of secondary TMAs often requires a multidisciplinary approach to tailor the best treatment strategy for each patient, depending on their specific underlying condition and health status.

organs like the kidneys.
- **Possible Side Effects**: There's a higher chance of getting severe infections, so doctors give a vaccine before starting this medicine. It can also cause headaches, nausea, and high blood pressure.

2. **Rituximab**:
- **What It Does**: This medicine targets certain immune cells, reducing the number of harmful antibodies that can cause clotting. It’s often used when TMAs are related to autoimmune problems.
- **Possible Side Effects**: Reactions during the medicine infusion like fever or chills, more infections, and sometimes severe skin reactions.

3. **Corticosteroids**:
- **What It Does**: These medicines calm down the immune system and reduce swelling, which can help in cases where the body’s defense system is causing problems.
- **Possible Side Effects**: Getting sick more easily, higher blood sugar, gaining weight, mood changes, and weak bones if used for a long time.

4. **Immunosuppressants (like Cyclophosphamide, Mycophenolate Mofetil)**:
- **What It Does**: These medicines help control the immune system by stopping immune cells from growing too much. They are used to treat autoimmune diseases that can lead to TMAs.
- **Possible Side Effects**: Lowering the body’s ability to fight infections, possible damage to the liver and kidneys, and stomach upset.

5. **Plasma Exchange**:
- **What It Does**: This treatment involves taking out the patient’s blood plasma (which might have bad substances) and replacing it with new plasma. This helps quickly reduce substances that cause clotting.
- **Possible Side Effects**: Bleeding due to blood thinners used during the procedure, low blood pressure, risk of infections, and allergic reactions to the new plasma.

What to Think About:
The best treatment depends on what's causing the TMA. If a medicine is the problem, stopping that medicine is important. If an infection is causing it, treating that infection is key.

Keeping an Eye on Things:
Doctors need to watch closely how treatments are working. They regularly check how the kidneys and other parts of the body are doing, and they might need to adjust treatments based on these checks.

Extra Help:
Sometimes extra treatments like blood transfusions for anemia (low red blood cells) or special care for blood pressure and kidneys are needed.

Treating Secondary TMAs usually needs a team of doctors, including specialists for the specific cause and kidney doctors, to make sure the treatment fits the patient's needs. The main goals are to handle the underlying condition causing the clots, stop more clots from forming, and keep the organs working well.

50	Cryofibrinogen-related MPGN	Cryofibrinogen-related Membranoproliferative Glomerulonephritis (MPGN) is a rare condition characterized by the accumulation of cryofibrinogen—abnormal proteins that precipitate in cold temperatures—in the small blood vessels of the kidneys. This leads to inflammation and damage, affecting kidney function. Treatment aims to manage symptoms, reduce inflammation, and prevent further accumulation of cryofibrinogen.<br><br>### Medications Commonly Used in Treatment:<br><br>1. **Corticosteroids (e.g., Prednisone)**:<br>- **Mechanism of Action**: Corticosteroids are anti-inflammatory agents that suppress the immune system and reduce inflammation in the kidneys. They help decrease the production of proteins that form cryofibrinogen.<br>- **Potential Side Effects**: Increased risk of infections, elevated blood sugar levels, weight gain, increased appetite, mood swings, and long-term effects such as bone weakening (osteoporosis).<br><br>2. **Cyclophosphamide**:<br>- **Mechanism of Action**: Cyclophosphamide is a cytotoxic drug that suppresses the immune system by interfering with DNA synthesis in cells, particularly those of the immune system. This reduces the production of abnormal proteins that can lead to cryofibrinogen formation.<br>- **Potential Side Effects**: Bone marrow suppression (leading to reduced white and red blood cell counts), increased risk of infections, potential for bladder toxicity (hemorrhagic cystitis), and a risk of secondary cancers.<br><br>3. **Plasma Exchange**:<br>- **Mechanism of Action**: Plasma exchange is a procedure that involves removing plasma that contains cryofibrinogen and other harmful antibodies from the blood, and replacing it with fresh plasma or a plasma substitute. This can rapidly decrease the level of circulating cryofibrinogen and alleviate symptoms.<br>- **Potential Side Effects**: Low blood pressure, bleeding due to anticoagulants used during the procedure, risk of infection, and possible allergic reactions to the plasma substitutes.<br><br>4. **Rituximab**:<br>- **Mechanism of Action**: Rituximab is a monoclonal antibody that targets CD20 on B lymphocytes, which are a type of white blood cell involved in the immune response, including the production of cryofibrinogen. By reducing these cells, Rituximab can decrease the levels of harmful antibodies and other proteins.<br>- **Potential Side Effects**: Infusion reactions, increased risk of infections, and potentially severe skin and mouth reactions.<br><br>5. **Immunosuppressants (such as Mycophenolate Mofetil)**:<br>- **Mechanism of Action**: These drugs inhibit the proliferation of immune cells, reducing the immune response and decreasing the production of proteins involved in inflammation and cryofibrinogen formation.<br>- **Potential Side Effects**: Myelosuppression, increased risk of infections, gastrointestinal disturbances, and potential liver toxicity.<br><br>### Considerations:<br>Treatment plans for Cryofibrinogen-related MPGN are highly individualized, depending on the severity of the disease and the patient's overall health. The goal is to control symptoms, maintain kidney function, and prevent complications.	Cryofibrinogen-related Membranoproliferative Glomerulonephritis (MPGN) is a rare kidney condition where abnormal proteins called cryofibrinogens build up in the small blood vessels of the kidneys. These proteins become solid in cold temperatures and can cause inflammation, hurting the kidneys. The main goal of treatment is to manage symptoms, reduce inflammation, and prevent more protein buildup.<br><br>### Medications Commonly Used in Treatment:<br><br>1. **Corticosteroids (like Prednisone)**:<br>- **What It Does**: These medicines help reduce swelling and calm down the immune system. They work to decrease the production of the proteins that can turn into cryofibrinogens.<br>- **Possible Side Effects**: Higher chance of getting infections, increased blood sugar, gaining weight, mood swings, and if used for a long time, weaker bones.<br><br>2. **Cyclophosphamide**:<br>- **What It Does**: This is a strong medicine that helps by stopping the immune system from making too many of the abnormal proteins that lead to problems.<br>- **Possible Side Effects**: Lower numbers of blood cells, which can make you get infections easier, bladder irritation, and a small chance of other serious diseases like cancer later on.<br><br>3. **Plasma Exchange**:<br>- **What It Does**: This treatment takes out the part of your blood that has the harmful proteins and replaces it with new plasma. This can quickly reduce the amount of bad proteins and help improve symptoms.<br>- **Possible Side Effects**: Feeling dizzy or lightheaded, bleeding where the needle goes in, risk of getting an infection, and allergic reactions to the new plasma.<br><br>4. **Rituximab**:<br>- **What It Does**: This medicine targets certain cells in your immune system that are involved in making the harmful proteins. By reducing these cells, it helps lower the production of these proteins.<br>- **Possible Side Effects**: Reactions during the medicine infusion like fever or chills, more infections, and sometimes severe skin reactions.<br><br>5. **Immunosuppressants (such as Mycophenolate Mofetil)**:<br>- **What It Does**: These medicines slow down the immune system to stop it from producing too many harmful proteins.<br>- **Possible Side Effects**: Lower immune defense making you more likely to catch infections, stomach upset, and possible liver problems.<br><br>### What to Think About:<br>Doctors need to create a treatment plan that fits the patient's specific needs based on how severe their condition is. The aim is to control the symptoms, keep the kidneys working well, and stop further damage.<br><br>### Keeping an Eye on Things:<br>Since the treatments can have serious side effects, doctors will regularly check the patient's health by testing blood, checking kidney function, and monitoring for any side effects. They might need to change the treatment based on how well it's

		### Monitoring and Adjustments:<br>Due to the potential severity of side effects, especially with medications that suppress the immune system, regular monitoring of blood counts, kidney function, and other vital signs is critical. Treatment regimens may need to be adjusted based on response and side effects.<br><br>This condition often requires a multidisciplinary approach, involving nephrologists, rheumatologists, and possibly hematologists, to manage the complex interactions of the immune system and kidney function effectively.	working or if it's causing problems.<br><br>Treating this kidney condition usually needs a team of doctors, including kidney specialists and others, to make sure the patient gets the best care possible. The main goals are to manage the symptoms, support kidney health, and prevent more issues.
51	C4 Glomerulopathy	C4 Glomerulopathy is a rare kidney disorder that is typically associated with abnormalities in the complement system, specifically the activation of the C4 component. This condition leads to the deposition of complement factors in the kidneys, causing inflammation and damage. Treatment primarily focuses on managing the abnormal complement activation and supporting kidney function.<br><br>### Medications Commonly Used in Treatment:<br><br>1. **Eculizumab**:<br>- **Mechanism of Action**: Eculizumab is a monoclonal antibody that inhibits the complement protein C5, preventing its split into C5a and C5b, which are potent inflammatory molecules. This inhibition helps reduce inflammation and prevents further damage to the kidney tissues.<br>- **Potential Side Effects**: Increased risk of meningococcal infections, necessitating vaccination before treatment. Other side effects include headache, nausea, and hypertension.<br><br>2. **Mycophenolate Mofetil**:<br>- **Mechanism of Action**: Mycophenolate Mofetil is an immunosuppressive agent that inhibits purine synthesis in lymphocytes, leading to reduced immune cell proliferation. This suppression helps reduce inflammation and immune-mediated damage in the kidneys.<br>- **Potential Side Effects**: Increased risk of infections, gastrointestinal disturbances (such as diarrhea), leukopenia (low white blood cell count), and potential liver toxicity.<br><br>3. **Corticosteroids (such as Prednisone)**:<br>- **Mechanism of Action**: Corticosteroids reduce inflammation by suppressing multiple inflammatory pathways at the molecular level. They also suppress the immune system globally, decreasing overall immune response and inflammation in the kidneys.<br>- **Potential Side Effects**: Increased risk of infections, hyperglycemia (high blood sugar), weight gain, mood swings, increased appetite, and long-term effects such as osteoporosis.<br><br>4. **Rituximab**:<br>- **Mechanism of Action**: Rituximab is a monoclonal antibody that targets CD20 on B lymphocytes, leading to their depletion. B cells are a component of the immune system involved in autoimmune responses and inflammation, and reducing their number can help manage conditions like C4 Glomerulopathy.<br>- **Potential Side Effects**: Infusion reactions, increased risk of infections, and rarely severe skin and mouth reactions.<br><br>### Additional Considerations:<br>The choice of therapy often depends on the individual patient's disease characteristics and overall health. Since C4 Glomerulopathy is associated with complement activation, treatments aim to modulate or inhibit this system to prevent further kidney damage.	C4 Glomerulopathy is a rare kidney disease linked to problems with the complement system—a part of the immune system that usually helps the body fight infections but can cause harm when it doesn't work right. This disease leads to harmful substances building up in the kidneys, which can cause inflammation and damage. The treatment aims to manage these problems and keep the kidneys working well.<br><br>### Medications Commonly Used in Treatment:<br><br>1. **Eculizumab**:<br>- **What It Does**: This medicine stops a specific part of the complement system (a protein called C5) from splitting into smaller parts that can cause inflammation. By doing this, it helps reduce swelling and prevents more damage to the kidneys.<br>- **Possible Side Effects**: It can increase the risk of getting severe infections like meningitis, so doctors usually give a vaccine before starting this medicine. Other side effects might include headaches, feeling sick to your stomach, and high blood pressure.<br><br>2. **Mycophenolate Mofetil**:<br>- **What It Does**: This medicine calms the immune system by stopping certain cells from growing too much, which can help reduce inflammation and damage in the kidneys.<br>- **Possible Side Effects**: There's a higher chance of getting infections, stomach issues like diarrhea, low white blood cell counts (which help fight infections), and potential liver problems.<br><br>3. **Corticosteroids (like Prednisone)**:<br>- **What It Does**: These are strong medicines that reduce swelling by calming down many parts of the immune system. They help lower the overall immune response, which can decrease inflammation in the kidneys.<br>- **Possible Side Effects**: More chances of getting infections, higher blood sugar levels, weight gain, mood changes, increased appetite, and with long use, weaker bones.<br><br>4. **Rituximab**:<br>- **What It Does**: This medicine targets specific immune cells (B cells) and reduces their numbers, which helps control the immune system's attack on the kidneys.<br>- **Possible Side Effects**: Reactions during the treatment like fever or chills, more infections, and sometimes severe skin reactions.<br><br>### What to Think About:<br>Doctors choose the best treatment based on how the disease is affecting the patient. Because this kidney problem is rare, doctors often need to adjust the

		### Monitoring and Adjustments:<br>Patients undergoing treatment require regular monitoring, including blood tests to check kidney function, complement activity levels, and potential side effects of the medications. Adjustments in therapy might be necessary based on these findings and the patient's response to treatment.<br><br>This condition's rarity necessitates a tailored approach to therapy, often involving nephrologists and possibly rheumatologists or immunologists to manage the complex interactions between the immune system and kidney function effectively.	treatment plan as they see how the patient responds.<br><br>### Keeping an Eye on Things:<br>Patients will need regular checks, including blood tests to see how well the kidneys are working and to watch for side effects from the medicines. Changes to the treatment might be needed based on these tests.<br><br>Treating C4 Glomerulopathy usually involves a team of doctors, including kidney specialists and sometimes doctors who specialize in the immune system, to make sure the patient gets the best care possible. The main goals are to manage the disease and keep the kidneys healthy.
52	Collagen III Glomerulopathy	Collagen III Glomerulopathy is a very rare kidney disorder characterized by the abnormal deposition of type III collagen in the glomeruli, which are the filtering units of the kidneys. This leads to progressive kidney damage and potentially kidney failure. Given the rarity and specific nature of this condition, treatment is largely supportive and experimental, aiming to manage symptoms and slow the progression of kidney damage.<br><br>### Medications Commonly Used in Treatment:<br><br>1. **Angiotensin-Converting Enzyme (ACE) Inhibitors (e.g., Lisinopril, Enalapril)**:<br>- **Mechanism of Action**: ACE inhibitors work by blocking the conversion of angiotensin I to angiotensin II, a potent vasoconstrictor that also stimulates aldosterone secretion. By reducing angiotensin II levels, these drugs decrease blood pressure and reduce the workload on the kidneys, thereby limiting further damage.<br>- **Potential Side Effects**: Cough, elevated blood potassium levels, low blood pressure, and in rare cases, kidney dysfunction and swelling of tissues (angioedema).<br><br>2. **Angiotensin II Receptor Blockers (ARBs) (e.g., Losartan, Valsartan)**:<br>- **Mechanism of Action**: ARBs block the action of angiotensin II at its receptor, preventing its vasoconstrictive effects and reducing blood pressure and kidney stress.<br>- **Potential Side Effects**: Dizziness, high potassium levels, and occasional kidney function impairment.<br><br>3. **Corticosteroids**:<br>- **Mechanism of Action**: Corticosteroids are used for their anti-inflammatory effects to reduce inflammation in the kidneys caused by abnormal collagen deposition. They suppress the immune system and decrease inflammation.<br>- **Potential Side Effects**: Increased risk of infections, high blood sugar levels, weight gain, increased appetite, mood swings, and bone thinning with long-term use.<br><br>4. **Immunosuppressants (e.g., Cyclophosphamide, Mycophenolate Mofetil)**:<br>- **Mechanism of Action**: These drugs suppress the immune system's overall activity to reduce inflammation and prevent further damage to kidney tissues.<br>- **Potential Side Effects**: Increased risk of infections, bone marrow suppression, gastrointestinal issues, and potential liver toxicity.<br><br>### Considerations:<br>Due to the lack of specific treatments for Collagen III Glomerulopathy, these medications are used primarily to manage symptoms and slow disease progression rather than cure the disease. The effectiveness of these treatments can vary significantly from patient to patient.<br><br>### Monitoring and Adjustments:	Collagen III Glomerulopathy is a very rare kidney condition where a type of protein called type III collagen builds up in the tiny filters of the kidneys. This causes damage to the kidneys and can lead to them not working well. Since this disease is so rare, doctors mainly focus on treating symptoms and trying to slow down the damage to the kidneys.<br><br>### Medications Commonly Used in Treatment:<br><br>1. **Angiotensin-Converting Enzyme (ACE) Inhibitors (like Lisinopril, Enalapril)**:<br>- **What It Does**: These medicines help lower blood pressure by stopping a specific process in the body that can make blood pressure go up. Lower blood pressure means less stress on the kidneys, which can help protect them from more damage.<br>- **Possible Side Effects**: Some people might get a cough, feel dizzy, have higher levels of potassium in their blood, or in rare cases, have kidney problems or swelling.<br><br>2. **Angiotensin II Receptor Blockers (ARBs) (like Losartan, Valsartan)**:<br>- **What It Does**: These drugs work by blocking a substance in the body that raises blood pressure. By stopping this substance, they help keep blood pressure lower, which is better for the kidneys.<br>- **Possible Side Effects**: They can make people feel dizzy, raise potassium levels, and sometimes affect how well the kidneys work.<br><br>3. **Corticosteroids**:<br>- **What It Does**: These are strong medicines that reduce swelling and calm down the body's defense system. They help lessen inflammation in the kidneys caused by the abnormal collagen.<br>- **Possible Side Effects**: They can increase the chance of getting infections, raise blood sugar levels, cause weight gain, mood changes, and make bones weaker if used for a long time.<br><br>4. **Immunosuppressants (like Cyclophosphamide, Mycophenolate Mofetil)**:<br>- **What It Does**: These medicines lower the activity of the immune system to help reduce inflammation and prevent more damage to the kidney tissues.<br>- **Possible Side Effects**: They can make it easier to catch infections, lower blood cell counts (which can also increase infection risk), cause stomach issues, and potentially harm the liver.<br><br>### What to Think About:<br>These treatments are mainly used to manage symptoms and slow down the disease since there's no cure. How well these treatments work can vary a lot from one

		Patients with this condition require close monitoring for kidney function and blood pressure, along with regular assessments to evaluate the effectiveness of the treatment and adjust dosages as necessary. Due to the potential side effects, especially from immune-suppressing drugs, regular blood tests are needed to monitor for complications.<br><br>Because Collagen III Glomerulopathy is a complex condition often leading to chronic kidney disease, a multidisciplinary approach involving nephrologists, potentially rheumatologists or pathologists, and a supportive care team is essential to manage the disease effectively.	person to another.<br><br>### Keeping an Eye on Things:<br>Patients with this disease need to be checked often by doctors to see how their kidneys are doing and how well the treatment is working. They also need regular blood tests to watch for any side effects from the medicines.<br><br>Managing Collagen III Glomerulopathy often involves a team of different doctors, including kidney specialists and possibly other types of doctors, to make sure the patient gets the best care possible. The main goals are to manage the symptoms, keep the kidneys working, and prevent more problems.
53	Sickle Cell Nephropathy	Sickle Cell Nephropathy is a complication of sickle cell disease, where the abnormal, sickle-shaped red blood cells block blood flow in the small vessels of the kidneys. This leads to damage over time, affecting kidney function. Managing sickle cell disease effectively is crucial to prevent or slow the development of nephropathy. The focus is primarily on preventing sickling events and managing symptoms.<br><br>### Medications Commonly Used in Treatment:<br><br>1. **Hydroxyurea**:<br>- **Mechanism of Action**: Hydroxyurea increases the production of fetal hemoglobin (HbF), which reduces the formation of sickle hemoglobin (HbS). Fewer sickle cells mean less blockage in kidney vessels and less damage.<br>- **Potential Side Effects**: Bone marrow suppression (which can lead to a reduced number of white blood cells, red blood cells, and platelets), gastrointestinal issues, and possible skin changes.<br><br>2. **Angiotensin-Converting Enzyme (ACE) Inhibitors (e.g., Lisinopril, Enalapril)**:<br>- **Mechanism of Action**: ACE inhibitors help reduce protein in the urine—a common problem in sickle cell nephropathy—by lowering blood pressure in the kidneys' filtering units, thereby reducing their workload and preventing damage.<br>- **Potential Side Effects**: Dry cough, high potassium levels, low blood pressure, and in rare cases, kidney impairment and swelling of tissues (angioedema).<br><br>3. **Angiotensin II Receptor Blockers (ARBs) (e.g., Losartan, Valsartan)**:<br>- **Mechanism of Action**: ARBs also help reduce proteinuria and manage blood pressure in the kidneys by blocking the effects of angiotensin II, a chemical that narrows blood vessels.<br>- **Potential Side Effects**: Dizziness, high potassium levels, and occasional kidney function impairment.<br><br>4. **Endothelin Receptor Antagonists (e.g., Bosentan)**:<br>- **Mechanism of Action**: These medications block endothelin, a powerful vasoconstrictor that can contribute to high blood pressure and damage in the kidney's blood vessels. This helps protect the kidneys by improving blood flow and reducing pressure.<br>- **Potential Side Effects**: Liver function abnormalities, fluid retention, headache, and possible harm to the fetus in pregnant women.<br><br>5. **Iron Chelation Therapy (e.g., Deferasirox, Deferoxamine)**:<br>- **Mechanism of Action**: Used primarily to manage iron overload, which can occur from frequent blood transfusions in sickle cell patients. Managing iron levels helps prevent damage to various organs, including the kidneys.<br>- **Potential Side Effects**: Gastrointestinal disturbances, hearing and vision changes, and	Sickle Cell Nephropathy is a kidney problem that happens in people with sickle cell disease. In this condition, the abnormal, sickle-shaped red blood cells block the tiny blood vessels in the kidneys. This blockage can damage the kidneys over time. Treating sickle cell disease well is very important to help prevent or slow down this kidney damage. Here's how some common treatments work:<br><br>### Medications Commonly Used in Treatment:<br><br>1. **Hydroxyurea**:<br>- **What It Does**: This medicine helps make a special type of hemoglobin called fetal hemoglobin. This new hemoglobin doesn't sickle as much, which means fewer blockages in the kidney's blood vessels and less damage.<br>- **Possible Side Effects**: It can lower the number of blood cells, which can make you more likely to get infections or feel tired, and it might upset your stomach or change your skin.<br><br>2. **Angiotensin-Converting Enzyme (ACE) Inhibitors (like Lisinopril, Enalapril)**:<br>- **What It Does**: These medicines lower the pressure inside the kidneys, which helps them filter blood without extra stress. This can stop proteins from leaking into the urine, a common problem in sickle cell nephropathy.<br>- **Possible Side Effects**: Some people might get a dry cough, feel dizzy, have high potassium levels, or in rare cases, have kidney problems or swelling.<br><br>3. **Angiotensin II Receptor Blockers (ARBs) (like Losartan, Valsartan)**:<br>- **What It Does**: Like ACE inhibitors, these drugs help manage protein in the urine and lower blood pressure by blocking certain body chemicals that tighten blood vessels.<br>- **Possible Side Effects**: They can make you feel dizzy, raise your potassium levels, and sometimes affect how well your kidneys work.<br><br>4. **Endothelin Receptor Antagonists (like Bosentan)**:<br>- **What It Does**: These medicines stop endothelin, a substance that narrows blood vessels and raises blood pressure. By blocking endothelin, these drugs help keep blood vessels open, which is good for kidney health.<br>- **Possible Side Effects**: They can affect liver function, cause fluid retention or headaches, and are not safe for pregnant women.<br><br>5. **Iron Chelation Therapy (like Deferasirox, Deferoxamine)**:<br>- **What It Does**: This treatment is used to remove extra iron from the body, which can build up due to frequent blood transfusions. Removing excess iron helps prevent damage to various organs, including the kidneys.

		potential kidney and liver toxicity.<br><br>### Considerations:<br>The choice of medication often depends on the severity of sickle cell disease, the extent of kidney involvement, and individual patient factors. The overall goal is to manage sickle cell disease effectively to prevent the onset or progression of nephropathy.<br><br>### Monitoring and Adjustments:<br>Patients require regular monitoring of kidney function, blood pressure, and hematological parameters. Adjustments in medication may be necessary based on kidney function tests, side effects, and the effectiveness of the treatment.<br><br>Managing sickle cell nephropathy requires a comprehensive approach that involves not only treating the kidney issues but also optimizing the management of the underlying sickle cell disease.	- **Possible Side Effects**: It might upset your stomach, or affect your hearing and vision, and it can also be tough on your kidneys and liver.<br><br>### What to Think About:<br>Doctors pick the best medicine based on how severe the sickle cell disease is and how much the kidneys are affected. The main goal is to manage sickle cell disease well to avoid kidney problems.<br><br>### Keeping an Eye on Things:<br>Doctors will regularly check how well your kidneys are working, how your blood pressure is, and how your blood looks. They might change your medicine based on these checks.<br><br>Treating sickle cell nephropathy involves looking after both the kidney problems and the overall sickle cell disease with a team of doctors to make sure you get the best care.
54	Mesangial Proliferative GN without IgA Deposits	Mesangial Proliferative Glomerulonephritis (Mesangial Proliferative GN) without IgA deposits is a type of kidney disease characterized by the proliferation of mesangial cells in the kidneys. These cells are part of the kidney's filtration system, and their overgrowth can affect kidney function. The condition lacks the immunoglobulin A (IgA) deposits that are typically seen in IgA nephropathy, making its treatment slightly different. Management usually focuses on controlling symptoms, reducing inflammation, and preserving kidney function.<br><br>### Medications Commonly Used in Treatment:<br><br>1. **Corticosteroids (e.g., Prednisone)**:<br>- **Mechanism of Action**: Corticosteroids are potent anti-inflammatory drugs that reduce inflammation and suppress the immune system. They help decrease the proliferation of mesangial cells and reduce proteinuria (protein in urine), which can be a symptom of this condition.<br>- **Potential Side Effects**: Increased risk of infections, high blood sugar levels, increased appetite, weight gain, mood swings, and with long-term use, bone thinning (osteoporosis), and muscle weakness.<br><br>2. **Angiotensin-Converting Enzyme (ACE) Inhibitors (e.g., Lisinopril, Enalapril)**:<br>- **Mechanism of Action**: ACE inhibitors help lower blood pressure and reduce the workload on the kidneys by decreasing the pressure in the glomeruli (kidney filtration units). This reduction in pressure can decrease the amount of protein lost in urine and slow the progression of kidney disease.<br>- **Potential Side Effects**: Dry cough, elevated blood potassium levels, low blood pressure, and in rare cases, kidney dysfunction and swelling of tissues (angioedema).<br><br>3. **Angiotensin II Receptor Blockers (ARBs) (e.g., Losartan, Valsartan)**:<br>- **Mechanism of Action**: ARBs also lower blood pressure and reduce kidney stress by blocking the action of angiotensin II, a chemical that narrows blood vessels. This helps protect the kidney from further damage.<br>- **Potential Side Effects**: Similar to ACE inhibitors, including dizziness, high potassium levels, and potential kidney impairment.<br><br>4. **Immunosuppressants (e.g., Cyclophosphamide, Mycophenolate Mofetil)**:<br>- **Mechanism of Action**: These drugs suppress the immune system to reduce inflammation and prevent further damage to the kidney tissues. They are particularly used in	Mesangial Proliferative Glomerulonephritis (Mesangial Proliferative GN) without IgA deposits is a type of kidney disease where certain cells in the kidneys (called mesangial cells) grow too much. This can make it hard for the kidneys to filter blood properly. The treatment helps to control symptoms, lessen swelling, and keep the kidneys working well.<br><br>### Medications Commonly Used in Treatment:<br><br>1. **Corticosteroids (like Prednisone)**:<br>- **What It Does**: These are strong medicines that help reduce swelling and calm down the immune system. They help stop the mesangial cells from growing too much and lower the amount of protein that leaks into the urine, a common problem in this disease.<br>- **Possible Side Effects**: Can make it easier to get infections, raise blood sugar levels, increase appetite, cause weight gain, change moods, and with long use, make bones weaker.<br><br>2. **Angiotensin-Converting Enzyme (ACE) Inhibitors (like Lisinopril, Enalapril)**:<br>- **What It Does**: These medicines help lower blood pressure and make it easier for the kidneys to do their job by lowering the pressure inside the kidney filters. This helps slow down the damage to the kidneys and decreases protein in the urine.<br>- **Possible Side Effects**: Some people might get a dry cough, have higher potassium in their blood, feel dizzy, or in rare cases, have kidney problems or swelling.<br><br>3. **Angiotensin II Receptor Blockers (ARBs) (like Losartan, Valsartan)**:<br>- **What It Does**: Like ACE inhibitors, these drugs help manage blood pressure and reduce stress on the kidneys by stopping certain chemicals in the body that tighten blood vessels.<br>- **Possible Side Effects**: Similar to ACE inhibitors, including dizziness, high potassium levels, and sometimes affecting how well the kidneys work.<br><br>4. **Immunosuppressants (like Cyclophosphamide, Mycophenolate Mofetil)**:<br>- **What It Does**: These drugs lower the activity of the immune system to reduce swelling and prevent more damage to the kidney tissues.<br>- **Possible Side Effects**: Can increase the chance of getting infections, lower

		more severe cases where inflammation is significant.<br>- **Potential Side Effects**: Increased risk of infections, myelosuppression (reduced bone marrow function), gastrointestinal disturbances, and potential liver toxicity.<br><br>5. **Fish Oil (Omega-3 Fatty Acids)**:<br>- **Mechanism of Action**: While not a standard pharmacological treatment, fish oil supplements can help reduce inflammation and have been studied for their potential protective effects on the kidneys.<br>- **Potential Side Effects**: Fish oil is generally well-tolerated but can cause gastrointestinal symptoms like belching, indigestion, or diarrhea. High doses can also thin the blood, increasing the risk of bleeding.<br><br>### Considerations:<br>The choice of treatment depends on the severity of the symptoms and the extent of kidney damage. The treatment aims to control blood pressure, reduce proteinuria, and manage immune-related kidney damage.<br><br>### Monitoring and Adjustments:<br>Patients undergoing treatment for Mesangial Proliferative GN need regular monitoring of kidney function, blood pressure, and urinary protein levels. Adjustments to the treatment plan may be necessary based on the patient's response and any side effects encountered.<br><br>This condition often requires a comprehensive approach that might include a nephrologist, a dietitian, and possibly other specialists to manage the disease effectively. The goal is to prevent progression to more severe kidney damage or failure.	the body's ability to fight infections, cause stomach issues, and potentially harm the liver.<br><br>5. **Fish Oil (Omega-3 Fatty Acids)**:<br>- **What It Does**: Taking fish oil supplements might help reduce swelling and protect the kidneys, although it's not a typical medicine.<br>- **Possible Side Effects**: Generally safe but can cause stomach upset like belching or diarrhea. High doses might increase the risk of bleeding.<br><br>### What to Think About:<br>Doctors pick the best treatment based on how the disease is affecting the kidneys. The main goals are to control blood pressure, lower protein loss in urine, and stop the kidneys from getting worse.<br><br>### Keeping an Eye on Things:<br>Doctors will check on how well the kidneys are working, how the blood pressure is, and how much protein is in the urine. They might need to change the medicine based on these checks.<br><br>Treating this kidney condition usually involves help from different doctors, including kidney specialists, to make sure everything is managed well and to keep the kidneys as healthy as possible.
55	IgM Nephropathy	IgM Nephropathy is a rare kidney disorder characterized by the deposition of IgM antibodies in the mesangium of the glomeruli, which are the filtering units of the kidneys. This can lead to proteinuria (excess protein in urine) and hematuria (blood in urine), and potentially progressive kidney damage. The treatment is somewhat similar to other proteinuric kidney diseases and aims to manage symptoms, reduce proteinuria, and preserve kidney function.<br><br>### Medications Commonly Used in Treatment:<br><br>1. **Corticosteroids (e.g., Prednisone)**:<br>- **Mechanism of Action**: Corticosteroids are anti-inflammatory agents that reduce inflammation in the kidneys by suppressing the immune system. This helps to decrease the production of antibodies like IgM that contribute to inflammation and damage in the glomeruli.<br>- **Potential Side Effects**: Increased risk of infections, elevated blood sugar levels, weight gain, increased appetite, mood swings, and long-term effects like osteoporosis and muscle weakness.<br><br>2. **Angiotensin-Converting Enzyme (ACE) Inhibitors (e.g., Lisinopril, Enalapril)**:<br>- **Mechanism of Action**: ACE inhibitors help lower blood pressure and reduce the workload on the kidneys by decreasing the pressure in the glomeruli (kidney filtration units). This helps reduce proteinuria and can slow the progression of kidney damage.<br>- **Potential Side Effects**: Dry cough, elevated blood potassium levels, low blood pressure, and in rare cases, kidney dysfunction and swelling of tissues (angioedema).<br><br>3. **Angiotensin II Receptor Blockers (ARBs) (e.g., Losartan, Valsartan)**:<br>- **Mechanism of Action**: ARBs lower blood pressure and reduce kidney stress by blocking the action of angiotensin II, a substance that narrows blood vessels. This helps protect the	IgM Nephropathy is a rare kidney disease where a type of antibody called IgM builds up in the kidneys. This can lead to protein in the urine (proteinuria) and blood in the urine (hematuria), which may harm the kidneys over time. The goal of treatment is to manage symptoms, lower the amount of protein in the urine, and keep the kidneys working well.<br><br>### Medications Commonly Used in Treatment:<br><br>1. **Corticosteroids (like Prednisone)**:<br>- **What It Does**: These medicines help reduce swelling and calm down the immune system. By doing this, they lower the amount of IgM antibodies that are causing damage and inflammation in the kidneys.<br>- **Possible Side Effects**: Can make it easier to get infections, raise blood sugar levels, cause weight gain, increase appetite, mood changes, and with long use, make bones weaker and muscles weaker.<br><br>2. **Angiotensin-Converting Enzyme (ACE) Inhibitors (like Lisinopril, Enalapril)**:<br>- **What It Does**: These medicines help lower blood pressure and make it easier for the kidneys to do their job by lowering the pressure inside the kidney filters. This helps slow down the damage to the kidneys and decreases protein in the urine.<br>- **Possible Side Effects**: Some people might get a dry cough, have higher potassium in their blood, feel dizzy, or in rare cases, have kidney problems or swelling.<br><br>3. **Angiotensin II Receptor Blockers (ARBs) (like Losartan, Valsartan)**:<br>- **What It Does**: Like ACE inhibitors, these drugs help manage blood pressure and reduce stress on the kidneys by stopping certain chemicals in the body that

		kidneys from further damage.<br>- **Potential Side Effects**: Dizziness, high potassium levels, and occasionally kidney function impairment.<br><br>4. **Immunosuppressants (e.g., Cyclophosphamide, Mycophenolate Mofetil)**:<br>- **Mechanism of Action**: These drugs suppress the overall activity of the immune system, reducing the inflammation and antibody production that can contribute to kidney damage.<br>- **Potential Side Effects**: Increased risk of infections, bone marrow suppression (leading to reduced white and red blood cells), gastrointestinal disturbances, and potential liver toxicity.<br><br>5. **Fish Oil (Omega-3 Fatty Acids)**:<br>- **Mechanism of Action**: While not a standard treatment, fish oil supplements are thought to reduce inflammation and have been used experimentally in various types of glomerulonephritis.<br>- **Potential Side Effects**: Fish oil is generally well-tolerated but can cause gastrointestinal symptoms like belching, indigestion, or diarrhea. High doses can also thin the blood, increasing the risk of bleeding.<br><br>### Considerations:<br>The choice of therapy often depends on the severity of symptoms and the extent of kidney involvement. Treatment strategies typically focus on controlling hypertension, reducing proteinuria, and managing immune-related kidney damage.<br><br>### Monitoring and Adjustments:<br>Patients with IgM Nephropathy need regular monitoring, including assessments of kidney function, urine protein levels, and blood pressure. Treatment adjustments may be necessary based on the patient's response to therapy and any side effects encountered.<br><br>Managing IgM Nephropathy requires a comprehensive approach that might involve nephrologists, and possibly other specialists, to tailor the best possible management strategy for each patient. The overall goal is to slow disease progression and prevent complications.	tighten blood vessels.<br>- **Possible Side Effects**: Similar to ACE inhibitors, including dizziness, high potassium levels, and sometimes affecting how well the kidneys work.<br><br>4. **Immunosuppressants (like Cyclophosphamide, Mycophenolate Mofetil)**:<br>- **What It Does**: These drugs lower the activity of the immune system to reduce swelling and prevent more damage to the kidney tissues.<br>- **Possible Side Effects**: Can increase the chance of getting infections, lower the body's ability to fight infections, cause stomach issues, and potentially harm the liver.<br><br>5. **Fish Oil (Omega-3 Fatty Acids)**:<br>- **What It Does**: Taking fish oil supplements might help reduce swelling and protect the kidneys, although it's not a typical medicine.<br>- **Possible Side Effects**: Generally safe but can cause stomach upset like belching or diarrhea. High doses might increase the risk of bleeding.<br><br>### What to Think About:<br>Doctors choose the best treatment based on how the disease is affecting the kidneys. The main goals are to control blood pressure, lower protein loss in urine, and stop the kidneys from getting worse.<br><br>### Keeping an Eye on Things:<br>Doctors will check on how well the kidneys are working, how the blood pressure is, and how much protein is in the urine. They might need to change the medicine based on these checks.<br><br>Treating IgM Nephropathy involves a team of doctors, including kidney specialists, to make sure everything is managed well and to keep the kidneys as healthy as possible.
56	C1q Nephropathy	C1q Nephropathy is a rare glomerular disease characterized by the deposition of the C1q complement component in the kidneys, leading to proteinuria (excess protein in the urine) and potentially progressive kidney damage. The treatment strategy for C1q Nephropathy often parallels those for other proteinuric and immune-mediated kidney diseases, particularly those involving complement system dysregulation.<br><br>### Medications Commonly Used in Treatment:<br><br>1. **Corticosteroids (e.g., Prednisone)**:<br>- **Mechanism of Action**: Corticosteroids are potent anti-inflammatory drugs that reduce inflammation by suppressing the immune system. They help decrease the immune response that contributes to inflammation and damage in the kidneys.<br>- **Potential Side Effects**: Increased risk of infections, elevated blood sugar levels, weight gain, mood swings, increased appetite, and long-term effects such as osteoporosis and muscle weakness.<br><br>2. **Cyclophosphamide**:<br>- **Mechanism of Action**: Cyclophosphamide is a cytotoxic drug that suppresses the immune system by damaging the DNA of rapidly dividing cells, including those involved in the immune response. This reduces the overall immune activity and decreases inflammation and	C1q Nephropathy is a rare kidney condition where a part of the immune system called C1q gets stuck in the kidneys. This can cause too much protein in the urine and may harm the kidneys over time. The treatment helps to manage symptoms, reduce protein loss in urine, and protect kidney health.<br><br>### Medications Commonly Used in Treatment:<br><br>1. **Corticosteroids (like Prednisone)**:<br>- **What It Does**: These medicines help reduce swelling and calm down the immune system. By doing this, they help lower the immune response that causes inflammation and damage in the kidneys.<br>- **Possible Side Effects**: Can make it easier to get infections, raise blood sugar levels, cause weight gain, mood changes, increase appetite, and with long use, make bones weaker and muscles weaker.<br><br>2. **Cyclophosphamide**:<br>- **What It Does**: This is a strong medicine that helps by stopping the immune system from being too active. It does this by damaging the DNA of cells that are quickly dividing, which are often involved in causing swelling and damage.<br>- **Possible Side Effects**: Can lower the number of blood cells which makes it

		damage to the kidneys.<br>- **Potential Side Effects**: Bone marrow suppression (leading to reduced blood cell counts), increased risk of infections, potential for bladder toxicity (hemorrhagic cystitis), and a risk of secondary cancers.<br><br>3. **Mycophenolate Mofetil**:<br>- **Mechanism of Action**: Mycophenolate Mofetil inhibits purine synthesis in lymphocytes, leading to reduced proliferation of these immune cells. This suppression helps decrease the immune-mediated damage to the kidney.<br>- **Potential Side Effects**: Increased risk of infections, gastrointestinal disturbances (such as diarrhea), leukopenia (low white blood cell count), and potential liver toxicity.<br><br>4. **Rituximab**:<br>- **Mechanism of Action**: Rituximab is a monoclonal antibody that targets CD20 on B lymphocytes, leading to their depletion. Reducing the number of B cells decreases the production of antibodies that can contribute to inflammation and damage in the kidneys.<br>- **Potential Side Effects**: Infusion reactions, increased risk of infections, and rarely severe skin and mouth reactions.<br><br>5. **Angiotensin-Converting Enzyme (ACE) Inhibitors (e.g., Lisinopril, Enalapril)** and **Angiotensin II Receptor Blockers (ARBs) (e.g., Losartan, Valsartan)**:<br>- **Mechanism of Action**: Both ACE inhibitors and ARBs help lower blood pressure and reduce proteinuria by decreasing the pressure within the glomeruli and improving filtration efficiency. This can help protect kidney function and slow disease progression.<br>- **Potential Side Effects**: Dry cough (more common with ACE inhibitors), elevated potassium levels, low blood pressure, and in rare cases, kidney dysfunction and swelling of tissues (angioedema).<br><br>### Considerations:<br>The choice of treatment for C1q Nephropathy depends on the severity of symptoms and the extent of kidney involvement. Treatment aims to control proteinuria, manage blood pressure, and suppress immune system activity to reduce kidney damage.<br><br>### Monitoring and Adjustments:<br>Regular monitoring of kidney function, blood pressure, and urinary protein levels is essential. Adjustments in therapy may be necessary based on the patient’s response to treatment and any side effects encountered. Managing C1q Nephropathy requires a multidisciplinary approach and may involve nephrologists, rheumatologists, and potentially other specialists to provide comprehensive care.	easier to get infections, can irritate the bladder, and has a small chance of causing other cancers later on.<br><br>3. **Mycophenolate Mofetil**:<br>- **What It Does**: This medicine stops certain immune cells from growing too much. This helps lower the immune system's attack on the kidneys.<br>- **Possible Side Effects**: More likely to get infections, stomach problems like diarrhea, low white blood cell counts (which help fight infections), and can also affect the liver.<br><br>4. **Rituximab**:<br>- **What It Does**: This medicine targets specific immune cells called B cells and reduces their numbers. Fewer B cells mean fewer harmful antibodies that can cause kidney damage.<br>- **Possible Side Effects**: Can cause reactions during the treatment like fever or chills, more infections, and sometimes severe skin reactions.<br><br>5. **Angiotensin-Converting Enzyme (ACE) Inhibitors (like Lisinopril, Enalapril)** and **Angiotensin II Receptor Blockers (ARBs) (like Losartan, Valsartan)**:<br>- **What It Does**: Both of these types of medicines help lower blood pressure and reduce protein in the urine by easing the pressure in the tiny filters inside the kidneys. This helps keep the kidneys working well and slows down any damage.<br>- **Possible Side Effects**: They can cause a dry cough (more common with ACE inhibitors), higher potassium levels, low blood pressure, and sometimes affect how well the kidneys work.<br><br>### What to Think About:<br>Doctors pick the best treatment based on how the disease is affecting the kidneys. The main goals are to control blood pressure, lower protein loss in urine, and calm the immune system to protect the kidneys.<br><br>### Keeping an Eye on Things:<br>Doctors will regularly check how well the kidneys are working, what the blood pressure is, and how much protein is in the urine. They might need to change the medicine based on these checks.<br><br>Treating C1q Nephropathy usually involves different doctors, including kidney specialists, to make sure everything is managed well and to keep the kidneys as healthy as possible.
57	Lipoprotein Glomerulopathy	Lipoprotein Glomerulopathy is a rare kidney disorder characterized by the abnormal deposition of lipoproteins in the glomeruli, the kidney's filtering units. This leads to proteinuria (excess protein in the urine) and can progress to kidney failure. The disease is associated with genetic variations affecting the metabolism of lipoproteins. Treatment mainly focuses on managing lipid levels and protecting kidney function.<br><br>### Medications Commonly Used in Treatment:<br><br>1. **Fibrates (e.g., Fenofibrate, Gemfibrozil)**:<br>- **Mechanism of Action**: Fibrates primarily reduce triglycerides and increase HDL (high-density lipoprotein) cholesterol levels by activating a receptor called PPAR-alpha. This action helps to normalize lipid metabolism, reducing the deposition of lipoproteins in the kidneys.<br>- **Potential Side Effects**: Gastrointestinal symptoms like nausea and diarrhea, gallstones,	Lipoprotein Glomerulopathy is a rare kidney disease where abnormal fats, called lipoproteins, build up in the tiny filters of the kidneys. This causes too much protein to spill into the urine and can lead to kidney damage. The main goal of treatment is to manage the levels of these fats in the blood and protect the kidneys.<br><br>### Medications Commonly Used in Treatment:<br><br>1. **Fibrates (like Fenofibrate, Gemfibrozil)**:<br>- **What It Does**: These medicines help lower bad fat levels in the blood, especially triglycerides, and help increase good cholesterol by working on specific receptors in the body. This helps reduce the build-up of fats in the kidneys.<br>- **Possible Side Effects**: They might cause stomach upset, nausea, or diarrhea, increase the chance of getting gallstones, and sometimes affect the liver.

		and in rare cases, liver enzyme elevations and muscle pain or weakness.<br><br>2. **Statins (e.g., Atorvastatin, Simvastatin)**:<br>- **Mechanism of Action**: Statins inhibit an enzyme called HMG-CoA reductase, which is crucial for cholesterol synthesis in the liver. This reduction in cholesterol production helps lower LDL (low-density lipoprotein) cholesterol levels and potentially reduces lipoprotein deposits in the glomeruli.<br>- **Potential Side Effects**: Muscle pain, increased risk of diabetes, and liver dysfunction. Muscle symptoms are the most common side effect and can range from mild discomfort to a severe condition called rhabdomyolysis.<br><br>3. **Omega-3 Fatty Acids (Fish Oil)**:<br>- **Mechanism of Action**: Omega-3 fatty acids help reduce triglyceride levels in the blood, which can contribute to the abnormal lipoprotein profiles seen in lipoprotein glomerulopathy. They may also have anti-inflammatory effects that can benefit kidney function.<br>- **Potential Side Effects**: Fishy aftertaste, gastrointestinal disturbances such as bloating or diarrhea, and increased bleeding risk if taken in high doses.<br><br>4. **Warfarin**:<br>- **Mechanism of Action**: In some cases, warfarin has been used to manage lipoprotein glomerulopathy due to its effects on clotting factors and possibly lipoproteins. The exact mechanism by which it affects this condition is not well understood and is considered an unconventional approach.<br>- **Potential Side Effects**: Increased risk of bleeding, requiring regular monitoring of blood clotting times to ensure safety.<br><br>### Considerations:<br>The choice of treatment for lipoprotein glomerulopathy depends largely on lipid levels and kidney function. Managing lipid levels effectively is crucial to slowing the progression of the disease and preventing further kidney damage.<br><br>### Monitoring and Adjustments:<br>Regular monitoring of lipid profiles, kidney function, and potential drug side effects is essential. Adjustments in medication dosages or changes in therapeutic strategies may be necessary based on these monitoring results.<br><br>The rarity of lipoprotein glomerulopathy often means that treatment is tailored to individual cases, frequently requiring the involvement of a nephrologist and possibly a lipidologist or endocrinologist to manage the complex interactions of lipid metabolism and kidney function effectively.	2. **Statins (like Atorvastatin, Simvastatin)**:<br>- **What It Does**: Statins work by stopping the liver from making too much cholesterol. This helps lower the bad cholesterol and may help prevent more fat from building up in the kidneys.<br>- **Possible Side Effects**: They can cause muscle pain, raise the risk of diabetes, and sometimes cause liver problems. Muscle pain is the most common issue.<br><br>3. **Omega-3 Fatty Acids (Fish Oil)**:<br>- **What It Does**: These are special fats that can help lower the levels of triglycerides in the blood, which are a type of bad fat. They might also help reduce swelling and protect the kidneys.<br>- **Possible Side Effects**: They can leave a fishy taste in your mouth, cause stomach issues like bloating or diarrhea, and if taken in high doses, increase the risk of bleeding.<br><br>4. **Warfarin**:<br>- **What It Does**: Sometimes doctors use warfarin, a medicine that affects blood clotting, to help manage this kidney condition, though it's not very common and the reasons it might work aren't well understood.<br>- **Possible Side Effects**: The main risk with warfarin is it can make you bleed more easily, so doctors have to check your blood regularly to make sure it's safe.<br><br>### What to Think About:<br>Doctors choose the best medicines based on how much bad fat is in your blood and how well your kidneys are working. It's really important to control these fat levels to slow down the damage to your kidneys.<br><br>### Keeping an Eye on Things:<br>Doctors need to keep a close check on how much fat is in your blood, how well your kidneys are working, and watch for any side effects from the medicines. They might need to change the medicines or adjust the doses based on these checks.<br><br>Treating Lipoprotein Glomerulopathy usually involves working with kidney doctors and sometimes doctors who specialize in blood fats to make sure you get the best care. The main goals are to manage the fats in your blood and keep your kidneys as healthy as possible.
58	Fibronectin Glomerulopathy	Fibronectin Glomerulopathy is a rare genetic kidney disease characterized by the deposition of fibronectin—a large glycoprotein—within the kidneys. This condition leads to proteinuria (excessive protein in urine) and can progress to kidney failure. Due to the rarity of the disease, treatment options are limited and primarily aimed at managing symptoms and slowing the progression of the disease.<br><br>### Medications Commonly Used in Treatment:<br><br>1. **Angiotensin-Converting Enzyme (ACE) Inhibitors (e.g., Lisinopril, Enalapril)**:<br>- **Mechanism of Action**: ACE inhibitors work by blocking the conversion of angiotensin I to angiotensin II, a potent vasoconstrictor that also stimulates aldosterone secretion. This helps to lower blood pressure and reduce the workload on the kidneys, thereby reducing	Fibronectin Glomerulopathy is a rare kidney disease that happens when a special protein called fibronectin builds up in the kidneys. This causes too much protein to spill into the urine and can lead to kidney damage over time. The treatments focus on managing symptoms and trying to slow down the damage to the kidneys.<br><br>### Medications Commonly Used in Treatment:<br><br>1. **Angiotensin-Converting Enzyme (ACE) Inhibitors (like Lisinopril, Enalapril)**:<br>- **What It Does**: These medicines help lower blood pressure and make it easier for the kidneys to do their job by reducing the pressure inside the kidney filters. This helps lower the amount of protein lost in urine and protects the kidneys.<br>- **Possible Side Effects**: Some people might get a dry cough, have higher

		proteinuria and helping to protect kidney function.<br>- **Potential Side Effects**: Common side effects include a persistent dry cough, elevated blood potassium levels, low blood pressure, and in rare cases, kidney dysfunction and swelling of tissues (angioedema).<br><br>2. **Angiotensin II Receptor Blockers (ARBs) (e.g., Losartan, Valsartan)**:<br>- **Mechanism of Action**: ARBs block the effects of angiotensin II by preventing it from binding to its receptor on blood vessels. Like ACE inhibitors, this reduces blood pressure and helps decrease the amount of protein lost in urine, which can be beneficial in managing kidney disease.<br>- **Potential Side Effects**: Similar to ACE inhibitors, ARBs can cause dizziness, high potassium levels, and occasionally affect kidney function.<br><br>3. **Corticosteroids (e.g., Prednisone)**:<br>- **Mechanism of Action**: Corticosteroids are used for their anti-inflammatory properties. They can reduce inflammation in the kidneys and may help in controlling proteinuria in some kidney diseases, though their effectiveness specifically in Fibronectin Glomerulopathy may vary.<br>- **Potential Side Effects**: Long-term use can lead to increased risk of infections, high blood sugar levels, weight gain, mood swings, increased appetite, and bone thinning.<br><br>### Considerations:<br>The choice of therapy often depends on individual factors such as the extent of proteinuria, blood pressure levels, and overall kidney function. Given the genetic nature and rarity of Fibronectin Glomerulopathy, treatments are generally supportive and focused on symptom management and delaying progression to kidney failure.<br><br>### Monitoring and Adjustments:<br>Regular monitoring of kidney function, blood pressure, and urinary protein levels is essential for patients undergoing treatment. Adjustments to the medication regimen may be necessary based on the patient's response to treatment and any side effects encountered.<br><br>Managing Fibronectin Glomerulopathy typically involves a multidisciplinary approach, often including nephrologists and genetic counselors. The goals are to optimize kidney function, manage complications, and provide genetic counseling to affected families given the hereditary aspect of the disease.	potassium in their blood, feel dizzy, or in rare cases, have kidney problems or swelling.<br><br>2. **Angiotensin II Receptor Blockers (ARBs) (like Losartan, Valsartan)**:<br>- **What It Does**: These drugs also help manage blood pressure and reduce stress on the kidneys by stopping certain chemicals in the body that tighten blood vessels.<br>- **Possible Side Effects**: Similar to ACE inhibitors, including dizziness, high potassium levels, and sometimes affecting how well the kidneys work.<br><br>3. **Corticosteroids (like Prednisone)**:<br>- **What It Does**: These are strong medicines that reduce swelling and calm down the immune system. They might help lower the amount of protein in the urine, which can be a problem in kidney diseases.<br>- **Possible Side Effects**: Can make it easier to get infections, raise blood sugar levels, cause weight gain, mood changes, increase appetite, and with long use, make bones weaker.<br><br>### What to Think About:<br>Doctors choose the best treatment based on how much protein is in the urine, blood pressure, and overall kidney health. Since this kidney problem is rare, the treatments focus more on managing symptoms and trying to keep the kidneys working as long as possible.<br><br>### Keeping an Eye on Things:<br>Doctors will need to regularly check how well the kidneys are working, what the blood pressure is, and how much protein is in the urine. They might need to change the medicines based on these checks.<br><br>Treating Fibronectin Glomerulopathy usually involves different doctors, including kidney specialists, to make sure everything is managed well and to keep the kidneys as healthy as possible. The main goals are to manage the symptoms, protect the kidneys, and help with any complications.
59	Lecithin-Cholesterol Acyltransferase Deficiency Disease	Lecithin-Cholesterol Acyltransferase (LCAT) Deficiency Disease is a rare genetic disorder characterized by the body's inability to produce a functional enzyme called lecithin-cholesterol acyltransferase. This enzyme is crucial for the normal metabolism of lipids in the blood, specifically for converting free cholesterol into a form that can be carried away by lipoproteins. Without this enzyme, cholesterol accumulates in various tissues, leading to symptoms such as corneal opacities, anemia, proteinuria, and ultimately, kidney dysfunction.<br><br>### Medications Commonly Used in Treatment:<br><br>Due to the rarity and complexity of LCAT deficiency, specific treatments are limited and primarily focused on managing symptoms and complications associated with the disease. There are no FDA-approved treatments specifically targeting the enzyme deficiency itself, but some therapies aim to manage the lipid abnormalities and protect organ function.<br><br>1. **Statins (e.g., Atorvastatin, Simvastatin)**:<br>- **Mechanism of Action**: Statins inhibit HMG-CoA reductase, an enzyme in the liver that	Lecithin-Cholesterol Acyltransferase (LCAT) Deficiency Disease is a rare genetic condition where the body can't make enough of an enzyme called lecithin-cholesterol acyltransferase. This enzyme helps manage cholesterol in the blood by changing its form so it can be moved out of the blood by special carriers. Without this enzyme, cholesterol builds up in different parts of the body, leading to problems like cloudy eyes (corneal opacities), anemia, protein in the urine, and kidney issues.<br><br>### Medications Commonly Used in Treatment:<br><br>Since this disease is rare, there aren't any treatments specifically made to fix the enzyme problem yet, but doctors use some medicines to help manage symptoms and complications:<br><br>1. **Statins (like Atorvastatin, Simvastatin)**:<br>- **What It Does**: Statins help lower the amount of cholesterol the liver makes. This can help reduce overall cholesterol levels, which is important because the body

		is crucial for cholesterol synthesis. By lowering the production of cholesterol, statins can help reduce the total cholesterol levels in the blood, although their effectiveness in LCAT deficiency may be limited because the pathology is not due to overproduction of cholesterol but a metabolism issue.<br>- **Potential Side Effects**: Muscle pain, increased risk of diabetes, liver dysfunction, and in rare cases, a severe muscle breakdown condition called rhabdomyolysis.<br><br>2. **Omega-3 Fatty Acids (Fish Oil)**:<br>- **Mechanism of Action**: Omega-3 fatty acids can help reduce triglyceride levels in the blood and are often used as part of the management for dyslipidemias. They may provide cardiovascular benefits and help manage lipid profiles in LCAT deficiency.<br>- **Potential Side Effects**: Fishy aftertaste, gastrointestinal disturbances such as bloating or diarrhea, and increased bleeding risk if taken in high doses.<br><br>3. **Dietary Modifications**:<br>- **Mechanism of Action**: Adjusting the diet to lower fat intake and manage cholesterol levels can be crucial for patients with LCAT deficiency. This includes reducing saturated fats and possibly increasing intake of healthy fats that can help improve the lipid profile.<br>- **Potential Side Effects**: Dietary restrictions can sometimes lead to nutritional deficiencies if not well balanced, so consultation with a dietitian is recommended.<br><br>### Experimental Treatments:<br>There are ongoing research efforts looking into enzyme replacement therapy and gene therapy as potential direct treatments for LCAT deficiency, aiming to replace the deficient enzyme or correct the genetic defect causing the enzyme deficiency. However, these are still under investigation and not widely available.<br><br>### Monitoring and Adjustments:<br>Patients with LCAT deficiency require regular monitoring of lipid levels, kidney function, and liver function tests to adjust treatments as necessary and to prevent complications. The treatment plan often involves a multidisciplinary team including a nephrologist, cardiologist, and a genetic counselor.<br><br>Managing LCAT deficiency focuses on symptom control and preventing progression to more severe complications, such as renal failure. Each patient's treatment plan is highly personalized, depending on the severity of their symptoms and overall health status.	isn't handling cholesterol properly due to the enzyme problem.<br>- **Possible Side Effects**: These can include muscle pain, a higher chance of getting diabetes, liver problems, and a rare but serious muscle breakdown called rhabdomyolysis.<br><br>2. **Omega-3 Fatty Acids (Fish Oil)**:<br>- **What It Does**: Fish oil helps lower the amount of another type of fat called triglycerides in the blood and can be good for heart health. It's used to help manage fat levels in the blood for this condition.<br>- **Possible Side Effects**: Might cause a fishy taste in your mouth, stomach upset like bloating or diarrhea, and a higher risk of bleeding if taken in large amounts.<br><br>3. **Dietary Modifications**:<br>- **What It Does**: Eating less fat, especially bad fats, and more healthy fats can help manage cholesterol levels. This is important for patients with LCAT deficiency to help control the condition.<br>- **Possible Side Effects**: Changing diet suddenly can sometimes lead to not getting enough of certain nutrients, so it's important to work with a dietitian to make sure the diet stays balanced and healthy.<br><br>### Experimental Treatments:<br>Researchers are looking into new treatments like enzyme replacement therapy (adding the missing enzyme back into the body) and gene therapy (fixing the gene that causes the problem). These are not ready for regular use but are being studied to see if they could help in the future.<br><br>### Monitoring and Adjustments:<br>Patients need regular check-ups to watch their cholesterol and fat levels, how well their kidneys are working, and how their liver is doing. The treatment plan might need changes based on these check-ups.<br><br>Managing LCAT deficiency involves trying to control symptoms and prevent problems like kidney damage. Doctors use a team approach with specialists like kidney doctors and heart doctors to take care of patients with this condition. Each person's treatment is customized to their specific needs and health status.
60	Nephropathic Cystinosis	Nephropathic cystinosis is a rare genetic disorder characterized by the accumulation of cystine crystals in the cells of various organs, including the kidneys. This accumulation causes damage and dysfunction, particularly affecting the kidneys early in life, which can lead to kidney failure if not properly managed. The treatment for nephropathic cystinosis primarily involves managing cystine levels in the body to prevent or reduce organ damage.<br><br>### Medications Commonly Used in Treatment:<br><br>1. **Cysteamine (Cystagon, Procysbi)**:<br>- **Mechanism of Action**: Cysteamine works by converting cystine within the cells into cysteine and a cysteine-cysteamine mixed disulfide, which are more soluble and can be more easily removed from the cells. This helps to reduce the toxic levels of cystine in the cells, thereby preventing further damage to the kidneys and other organs.<br>- **Potential Side Effects**: Cysteamine can cause gastrointestinal symptoms like nausea, vomiting, and diarrhea. It may also cause bad breath, skin odor, lethargy, headaches, and, in some cases, bone marrow suppression, which can lead to issues like neutropenia (low white	Nephropathic cystinosis is a rare disease where a substance called cystine gets stuck inside cells in the body, building up and causing damage, especially in the kidneys. If not treated, it can lead to serious kidney problems and affect other parts of the body. The main goal of treatment is to lower the amount of cystine in the cells.<br><br>### Medications Commonly Used in Treatment:<br><br>1. **Cysteamine (Cystagon, Procysbi)**:<br>- **What It Does**: Cysteamine helps to break down the cystine in the cells into smaller parts that the body can get rid of more easily. This helps to stop the cystine from building up and causing damage.<br>- **Possible Side Effects**: It can cause stomach upset like nausea, vomiting, and diarrhea. It might also make your breath or skin smell different, make you feel tired, give you headaches, and sometimes affect your blood cells, making it easier to get sick.

		blood cell count). Long-term use has been associated with Ehlers-Danlos-like symptoms, such as hyperelastic skin and joint hypermobility.<br><br>2. **Cysteamine Eye Drops**:<br>- **Mechanism of Action**: These are used to treat corneal cystine crystal accumulation, which is common in cystinosis patients. The eye drops help to reduce the cystine crystal buildup directly in the cornea.<br>- **Potential Side Effects**: Local irritation, sensitivity to light, and possible eye discomfort or pain.<br><br>3. **Electrolyte and Mineral Supplements**:<br>- **Mechanism of Action**: Because cystinosis can lead to losses in essential minerals and salts through the kidneys (known as renal Fanconi syndrome), supplements such as potassium, phosphate, and magnesium, along with vitamin D and sometimes sodium bicarbonate, are used to correct these imbalances.<br>- **Potential Side Effects**: Depending on the supplement, side effects might include gastrointestinal issues, imbalances in other electrolytes, and in the case of sodium bicarbonate, possible metabolic alkalosis if not carefully monitored.<br><br>### Considerations:<br>Treatment strategies depend heavily on managing the systemic build-up of cystine to prevent organ damage. As cystinosis is a progressive condition that primarily affects the kidneys but can also impact other organs, comprehensive care from a multidisciplinary team is crucial. This team often includes nephrologists, ophthalmologists, and endocrinologists to manage the various aspects of the disease.<br><br>### Monitoring and Adjustments:<br>Regular monitoring of cystine levels in leukocytes is critical to ensure that cysteamine dosage is effective and that cystine levels are kept at target levels. Routine kidney function tests, eye examinations, and monitoring for the development of new symptoms or complications related to the disease or its treatment are also essential.<br><br>The goals are to manage the disease proactively, preserve kidney function for as long as possible, and address complications as they arise to improve quality of life and outcomes for patients with nephropathic cystinosis.	2. **Cysteamine Eye Drops**:<br>- **What It Does**: These drops help remove cystine crystals from the eyes, which can form in people with cystinosis. Using the drops helps keep the eyes healthy.<br>- **Possible Side Effects**: The drops might irritate the eyes, make them sensitive to light, or feel uncomfortable.<br><br>3. **Electrolyte and Mineral Supplements**:<br>- **What It Does**: Cystinosis can cause the body to lose important minerals and salts through the urine. Supplements like potassium, phosphate, and magnesium, along with vitamin D and sometimes sodium bicarbonate, help replace what's lost and keep the body balanced.<br>- **Possible Side Effects**: These supplements can sometimes cause stomach issues or upset the balance of other minerals and salts in the body.<br><br>### What to Think About:<br>Treating cystinosis means focusing on keeping the cystine levels low to prevent damage to the kidneys and other organs. Doctors will use medicines to reduce cystine levels and provide supplements to help with any minerals lost due to kidney problems.<br><br>### Keeping an Eye on Things:<br>Doctors will regularly check the levels of cystine in the blood to make sure the medicines are working. They'll also check how well the kidneys are working, look at the eyes for any signs of crystals, and watch for any new symptoms or side effects from the treatment.<br><br>The aim is to manage the disease carefully, keep the kidneys working as well as possible, and handle any other health issues that come up to improve the quality of life for someone with nephropathic cystinosis. This involves help from different types of doctors, like kidney specialists, eye doctors, and others who work together to provide the best care.
61	Idiopathic Nodular Glomerulosclerosis	Idiopathic Nodular Glomerulosclerosis (ING) is a rare kidney disorder characterized by the thickening and hardening of the blood vessels in the kidneys' filtering units, known as glomeruli. This condition resembles changes seen in diabetic nephropathy but occurs in patients without diabetes. The exact cause of ING is not fully understood, and treatment mainly focuses on managing symptoms and slowing the progression of the disease.<br><br>### Medications Commonly Used in Treatment:<br><br>1. **Angiotensin-Converting Enzyme (ACE) Inhibitors (e.g., Lisinopril, Enalapril)**:<br>- **Mechanism of Action**: ACE inhibitors work by blocking the conversion of angiotensin I to angiotensin II, a powerful substance that can tighten blood vessels. By lowering angiotensin II levels, these medications help relax blood vessels, reducing blood pressure and the workload on the kidneys. This can slow the progression of kidney damage and reduce proteinuria (protein in urine).<br>- **Potential Side Effects**: Common side effects include a persistent dry cough, elevated blood potassium levels, low blood pressure, and in rare cases, kidney dysfunction and swelling of tissues (angioedema).	Idiopathic Nodular Glomerulosclerosis (ING) is a rare kidney condition where the small blood vessels in the kidneys' filters, called glomeruli, get thick and hard. This problem looks similar to what happens in the kidneys of people with diabetes, but it happens in people without diabetes. The exact cause isn't known, and treatments focus on managing symptoms and slowing down the disease.<br><br>### Medications Commonly Used in Treatment:<br><br>1. **Angiotensin-Converting Enzyme (ACE) Inhibitors (like Lisinopril, Enalapril)**:<br>- **What It Does**: These medicines help relax blood vessels by lowering the levels of a substance that makes blood vessels tight. This makes it easier for the heart to pump blood and reduces pressure in the kidneys, which can help slow down kidney damage and reduce protein in the urine (a sign of kidney issues).<br>- **Possible Side Effects**: Some people might get a dry cough, have higher potassium in their blood, feel dizzy, or in rare cases, have kidney problems or swelling.

		2. **Angiotensin II Receptor Blockers (ARBs) (e.g., Losartan, Valsartan)**:<br>- **Mechanism of Action**: ARBs block the action of angiotensin II directly at its receptor sites on blood vessels, preventing it from exerting its blood vessel-tightening effects. Like ACE inhibitors, this helps to lower blood pressure and decrease kidney stress, thereby protecting the kidneys from further damage.<br>- **Potential Side Effects**: They can cause dizziness, high potassium levels, and occasionally affect kidney function. They are generally better tolerated regarding the cough associated with ACE inhibitors.<br><br>3. **Statins (e.g., Atorvastatin, Simvastatin)**:<br>- **Mechanism of Action**: Statins are primarily used to lower cholesterol levels in the blood. They work by inhibiting an enzyme involved in cholesterol synthesis in the liver. By reducing cholesterol, statins can help prevent further vascular damage in the kidneys which might be contributing to the sclerosis and fibrosis.<br>- **Potential Side Effects**: Statins are generally well tolerated, but they can cause muscle pain, increased risk of diabetes, and liver dysfunction. Rarely, they can lead to a serious condition known as rhabdomyolysis.<br><br>### Considerations:<br>The treatment strategy for ING aims to address the underlying vascular changes and protect the kidneys from further damage. By managing blood pressure and reducing proteinuria, these medications not only help slow disease progression but also address some of the common cardiovascular risks associated with kidney diseases.<br><br>### Monitoring and Adjustments:<br>Patients with ING require regular monitoring of their kidney function, blood pressure, electrolytes, and proteinuria levels. Treatment adjustments may be necessary based on the patient’s response to therapy and the progression of kidney disease.<br><br>Overall, the management of Idiopathic Nodular Glomerulosclerosis involves a combination of cardiovascular risk management, blood pressure control, and careful monitoring of kidney function. Collaboration with a nephrologist is essential to optimize treatment and manage this condition effectively.	2. **Angiotensin II Receptor Blockers (ARBs) (like Losartan, Valsartan)**:<br>- **What It Does**: These drugs also help relax blood vessels by stopping the substance that tightens them from working. This helps lower blood pressure and reduces stress on the kidneys, protecting them from further damage.<br>- **Possible Side Effects**: They can cause dizziness, high potassium levels, and sometimes affect how well the kidneys work. They're usually easier to handle than ACE inhibitors because they're less likely to cause a cough.<br><br>3. **Statins (like Atorvastatin, Simvastatin)**:<br>- **What It Does**: Statins are used to lower cholesterol levels. High cholesterol can hurt blood vessels, so lowering it helps protect the kidneys and other blood vessels in the body.<br>- **Possible Side Effects**: These medicines are generally safe but can cause muscle pain, a higher chance of getting diabetes, and liver issues. Very rarely, they can lead to a serious muscle problem.<br><br>### What to Think About:<br>Doctors use these medicines to help manage blood pressure and protect the kidneys from getting worse. They also help lower risks related to heart health, which is important when dealing with kidney diseases.<br><br>### Keeping an Eye on Things:<br>Kids with ING need to have regular check-ups to see how their kidneys are doing, what their blood pressure is like, and to check their blood for any signs of problems from the medicines. Sometimes doctors need to change the medicines based on these check-ups.<br><br>Treating ING means working with a kidney doctor to watch the condition closely and make sure the treatments are helping without causing other problems. The main goals are to manage the symptoms, keep the kidneys working as well as possible, and take care of the patient's overall health.
62	Scleroderma Renal Crisis	Scleroderma Renal Crisis (SRC) is a severe complication of systemic sclerosis (scleroderma), characterized by an abrupt increase in blood pressure and rapid deterioration of kidney function. This condition is considered an emergency and requires immediate treatment to prevent irreversible kidney damage and other serious outcomes.<br><br>### Medications Commonly Used in Treatment:<br><br>1. **Angiotensin-Converting Enzyme (ACE) Inhibitors (e.g., Captopril, Enalapril)**:<br>- **Mechanism of Action**: ACE inhibitors block the conversion of angiotensin I to angiotensin II, a potent vasoconstrictor that increases blood pressure and contributes to kidney damage. By inhibiting this conversion, ACE inhibitors help lower blood pressure and improve blood flow through the kidneys, potentially reducing the risk of further kidney damage.<br>- **Potential Side Effects**: Common side effects include a persistent dry cough, hyperkalemia (high potassium levels), low blood pressure, and in rare cases, kidney dysfunction and swelling of tissues (angioedema). Careful monitoring is necessary, especially when starting therapy, to adjust dosages and avoid acute kidney failure.	Scleroderma Renal Crisis (SRC) is a serious problem that happens in people with a disease called scleroderma. It causes very high blood pressure and quick damage to the kidneys. It's very important to treat this right away to stop the kidneys from getting worse.<br><br>### Medications Commonly Used in Treatment:<br><br>1. **Angiotensin-Converting Enzyme (ACE) Inhibitors (like Captopril, Enalapril)**:<br>- **What It Does**: These medicines help lower blood pressure by stopping a chemical in your body that makes blood vessels tight. When blood vessels are more relaxed, blood pressure goes down, and it's easier for blood to flow through your kidneys, which helps protect them.<br>- **Possible Side Effects**: Some people might get a dry cough, have higher potassium levels, feel dizzy, or in rare cases, have swelling or kidney problems. Doctors watch closely when you start this medicine to make sure it's safe.<br><br>2. **Calcium Channel Blockers (like Nifedipine, Amlodipine)**:<br>- **What It Does**: These drugs help lower blood pressure by stopping calcium

		2. **Calcium Channel Blockers (e.g., Nifedipine, Amlodipine)**:<br>- **Mechanism of Action**: These drugs relax and widen blood vessels by blocking calcium channels in vascular smooth muscle. This reduces the resistance in peripheral blood vessels, helping to lower blood pressure. They are often used when ACE inhibitors are insufficient or as additional therapy.<br>- **Potential Side Effects**: Potential side effects include headache, flushing, dizziness, rapid heart rate (tachycardia), and swelling in the legs due to fluid accumulation (edema).<br><br>### Additional Treatments and Considerations:<br>- **Blood Pressure Monitoring**: Frequent monitoring of blood pressure is crucial for patients with SRC. Effective control of hypertension is key to managing this condition.<br>- **Kidney Function Monitoring**: Regular monitoring of kidney function through blood tests and urine analysis helps assess the progression of the disease and the effectiveness of treatment.<br><br>### Intensive Care:<br>Patients with severe SRC might require treatment in an intensive care unit (ICU) to manage acute complications and stabilize their condition. This includes the use of intravenous medications to rapidly control blood pressure if not responsive to oral medications and to manage potential complications like heart failure or severe renal impairment.<br><br>### Preventive Measures:<br>- For patients with scleroderma, regular monitoring and early intervention at signs of renal involvement can prevent the onset of a full-blown renal crisis.<br>- Educating patients about the importance of regular blood pressure monitoring and adherence to prescribed medications is vital.<br><br>Managing scleroderma renal crisis involves a multidisciplinary approach, including rheumatologists, nephrologists, and sometimes cardiologists, to address the complex needs of these patients and optimize their treatment outcomes.	from entering the muscles in the walls of your blood vessels. This makes the blood vessels relax and widen, which helps lower blood pressure.<br>- **Possible Side Effects**: These can include headaches, feeling warm and red in the face (flushing), feeling dizzy, a fast heartbeat, and swelling in the legs.<br><br>### Extra Care Needed:<br>- **Checking Blood Pressure**: It's really important for people with SRC to check their blood pressure often. Keeping blood pressure under control is a big part of treating this condition.<br>- **Checking Kidneys**: Doctors will also regularly test how well the kidneys are working by checking your blood and urine.<br><br>### Intensive Care:<br>Sometimes, if SRC is very severe, patients might need to go to the intensive care unit (ICU) in the hospital. Here, doctors can give strong medicines through an IV (a tube that goes into a vein) to quickly lower blood pressure and manage other serious problems that might happen.<br><br>### Preventing Problems:<br>- For people with scleroderma, watching for any signs of kidney problems and keeping blood pressure low is key to avoiding a crisis.<br>- Learning about the importance of taking medicine correctly and keeping track of blood pressure is very important for staying healthy.<br><br>Treating scleroderma renal crisis involves doctors from different specialties, like kidney doctors (nephrologists) and arthritis doctors (rheumatologists), who work together to take care of you and help you feel better.
63	Oxalate Nephropathy	Oxalate nephropathy is a condition where high levels of oxalate accumulate in the kidneys, leading to kidney damage. This can occur due to increased intake or absorption of oxalate from the diet, or from the body producing too much oxalate, often due to genetic conditions. Treatment focuses on reducing oxalate levels in the body and managing kidney function.<br><br>### Medications Commonly Used in Treatment:<br><br>1. **Potassium Citrate**:<br>- **Mechanism of Action**: Potassium citrate is used to alkalinize the urine, which can prevent the formation of oxalate stones. By increasing the pH of the urine, potassium citrate reduces the crystallization of urinary oxalate, helping to prevent further kidney damage.<br>- **Potential Side Effects**: Gastrointestinal symptoms such as abdominal discomfort, nausea, vomiting, and diarrhea are common. High doses may lead to hyperkalemia (high potassium levels), which can affect heart rhythm.<br><br>2. **Calcium Supplements (Calcium Citrate)**:<br>- **Mechanism of Action**: Calcium supplements are used to bind dietary oxalate in the gut, preventing its absorption into the bloodstream and subsequent deposition in the kidneys. Calcium citrate is preferred as it can help keep the urine alkalinized as well.<br>- **Potential Side Effects**: Constipation is a common side effect. Excessive calcium can also lead to hypercalcemia (high calcium levels), which can cause renal function impairment and other metabolic disturbances.	Oxalate nephropathy is a kidney condition where too much of a substance called oxalate builds up in the kidneys. This can hurt the kidneys because oxalate can form crystals that damage kidney cells. The treatment aims to lower the amount of oxalate in the body and keep the kidneys working well.<br><br>### Medications Commonly Used in Treatment:<br><br>1. **Potassium Citrate**:<br>- **What It Does**: This medicine helps make your urine less acidic, which can stop oxalate stones from forming. By making the urine a bit more basic, it's harder for oxalate to turn into crystals.<br>- **Possible Side Effects**: It can cause stomach upset, like belly pain, nausea, or diarrhea. If you take too much, it might lead to too much potassium in your blood, which can affect your heart rhythm.<br><br>2. **Calcium Supplements (like Calcium Citrate)**:<br>- **What It Does**: Calcium can grab onto oxalate from the food in your gut, which keeps it from getting into your blood and going to your kidneys. This can help reduce the oxalate that builds up in the kidneys.<br>- **Possible Side Effects**: Taking calcium can sometimes make you constipated. Taking too much calcium can lead to too much calcium in your blood, which can cause other health problems.

		3. **Pyridoxine (Vitamin B6)**:<br>- **Mechanism of Action**: For patients with primary hyperoxaluria, a genetic condition that leads to excessive production of oxalate, pyridoxine can be effective. It reduces the production of oxalate by modulating the enzyme AGT (alanine:glyoxylate aminotransferase), which is involved in glyoxylate metabolism.<br>- **Potential Side Effects**: High doses of pyridoxine can lead to sensory neuropathy, which is usually reversible upon discontinuation of the drug.<br><br>4. **Water Intake**:<br>- **Mechanism of Action**: Increasing water intake helps to dilute the urine, reducing the concentration of oxalate and lowering the risk of oxalate crystal formation.<br>- **Potential Side Effects**: Excessive water intake can lead to water intoxication, though this is rare and typically occurs only with very high volumes.<br><br>### Considerations:<br>Managing oxalate nephropathy requires careful monitoring of kidney function and regular assessment of urine and blood chemistry to ensure effectiveness of the treatment and to adjust as necessary. Dietary management, including reducing foods high in oxalates (such as spinach, rhubarb, and beets), is also crucial.<br><br>### Monitoring and Adjustments:<br>Patients will need regular monitoring to assess the effectiveness of these treatments on oxalate levels and kidney function. Adjustments to diet, medication dosages, and overall treatment strategies may be necessary based on lab results and clinical outcomes.<br><br>Oxalate nephropathy often requires collaboration between nephrologists, nutritionists, and sometimes genetic counselors, particularly when dealing with inherited forms of the disease. The overarching goal is to reduce oxalate levels in the body, prevent further kidney damage, and manage any underlying conditions contributing to the disease.	3. **Pyridoxine (Vitamin B6)**:<br>- **What It Does**: In some people, especially those with a genetic condition that makes them produce too much oxalate, Vitamin B6 can help lower the amount of oxalate made by the body.<br>- **Possible Side Effects**: If you take a lot of Vitamin B6, it can cause problems with your nerves, but this usually goes away if you stop taking so much.<br><br>4. **Water Intake**:<br>- **What It Does**: Drinking a lot of water can help dilute your urine. With more water in your urine, oxalate is less likely to form crystals because it's not as concentrated.<br>- **Potential Side Effects**: Drinking an extreme amount of water in a short time can be harmful, but it's rare. It can lead to a condition where your body's salt levels get too low.<br><br>### What to Think About:<br>People with this condition need to watch what they eat and drink. Avoiding foods high in oxalates, like spinach, nuts, and chocolate, can help. It's also important to take your medicine as your doctor tells you and to drink plenty of water.<br><br>### Keeping an Eye on Things:<br>Doctors will need to check your kidney function regularly with blood and urine tests to see how well the treatment is working. They may need to adjust your medicine or diet based on these tests.<br><br>Treating oxalate nephropathy involves working with doctors, like kidney specialists, and sometimes dietitians, to make sure you stay as healthy as possible. The main goals are to lower the oxalate in your body and to keep your kidneys working well.
64	IgG4-relared Disease	IgG4-related disease is a chronic inflammatory condition characterized by the infiltration of IgG4-producing plasma cells and associated fibrosis (scarring) in various organs. This disease can affect multiple organ systems, including the pancreas, kidneys, salivary glands, and lymph nodes. Treatment primarily aims to reduce inflammation, prevent organ damage, and manage symptoms.<br><br>### Medications Commonly Used in Treatment:<br><br>1. **Corticosteroids (e.g., Prednisone)**:<br>- **Mechanism of Action**: Corticosteroids are the first line of treatment and work by broadly suppressing inflammation and the immune response. They are effective at reducing the number of IgG4-producing cells and the associated fibrosis.<br>- **Potential Side Effects**: Long-term use can lead to side effects such as increased risk of infections, diabetes, osteoporosis, weight gain, hypertension, and mood changes.<br><br>2. **Rituximab**:<br>- **Mechanism of Action**: Rituximab is a monoclonal antibody that targets CD20 on B lymphocytes, leading to their depletion. Since B cells are a source of IgG4, reducing their number can decrease the production of IgG4 and help control the disease.<br>- **Potential Side Effects**: Infusion reactions, increased risk of infections, and, rarely, severe mucocutaneous reactions. Patients are often premedicated with steroids, acetaminophen, and antihistamines to reduce these risks.	IgG4-related disease is a condition where a type of immune cell called IgG4 gets into different parts of the body and causes swelling and scarring. This can happen in places like the pancreas, kidneys, and glands in the neck. The goal of treatment is to reduce this swelling, stop the scarring, and keep the organs working well.<br><br>### Medications Commonly Used in Treatment:<br><br>1. **Corticosteroids (like Prednisone)**:<br>- **What It Does**: These are strong medicines that help calm down the immune system and reduce swelling quickly. They are usually the first treatment doctors use.<br>- **Possible Side Effects**: If you take these for a long time, they can cause some problems like easier infections, higher blood sugar, weaker bones, weight gain, high blood pressure, and mood changes.<br><br>2. **Rituximab**:<br>- **What It Does**: Rituximab is a special type of medicine that targets and reduces certain immune cells that make IgG4, which can help control the disease.<br>- **Possible Side Effects**: It can cause reactions when you get the infusion (like fever or chills), increase the risk of getting infections, and very rarely, cause severe skin reactions. Doctors often give medicines before the infusion to help reduce these side effects.

		3. **Azathioprine and Mycophenolate Mofetil**:<br>- **Mechanism of Action**: These immunosuppressive agents are used as steroid-sparing medications to reduce the need for long-term corticosteroid use. They inhibit purine synthesis, which is crucial for the proliferation of cells that contribute to the immune response and inflammation.<br>- **Potential Side Effects**: Increased susceptibility to infections, liver toxicity, gastrointestinal symptoms, and bone marrow suppression.<br><br>4. **Methotrexate**:<br>- **Mechanism of Action**: Methotrexate is another immunosuppressive agent that inhibits dihydrofolate reductase, leading to a reduction in DNA synthesis and cellular proliferation. This helps control inflammation and immune system activity.<br>- **Potential Side Effects**: Liver toxicity, bone marrow suppression, fatigue, and mucositis. Regular monitoring of liver function and complete blood counts is necessary.<br><br>### Additional Treatments:<br>In some cases, especially when critical organs are involved or there is resistance to standard therapies, other immunosuppressive agents or biologics may be considered. The choice of medication depends on the specific organs involved and the severity of the disease.<br><br>### Considerations:<br>The goal of therapy is to induce remission with an initial high dose of corticosteroids, followed by a gradual tapering while monitoring for symptoms and signs of relapse. Maintenance therapy with immunosuppressive drugs may be necessary to prevent relapse, particularly in severe cases.<br><br>### Monitoring and Adjustments:<br>Regular follow-up with blood tests to monitor inflammation markers, organ function tests, and imaging studies are essential to assess the response to treatment and adjust medications accordingly. Monitoring is also critical to manage side effects associated with long-term immunosuppression.<br><br>IgG4-related disease requires a multidisciplinary approach, often involving rheumatologists, gastroenterologists, nephrologists, and other specialists, depending on the organs affected. This collaborative care is crucial for effectively managing this complex and multi-system disease.	3. **Azathioprine and Mycophenolate Mofetil**:<br>- **What It Does**: These medicines help reduce the need to use steroids for a long time. They work by slowing down the immune system in a different way to prevent swelling and damage.<br>- **Possible Side Effects**: They can make it easier to get infections, cause stomach upset, and affect the liver or blood cells, so doctors will keep a close watch with regular blood tests.<br><br>4. **Methotrexate**:<br>- **What It Does**: Methotrexate also helps control the immune system and reduce swelling. It's used when other medicines might not be suitable.<br>- **Possible Side Effects**: It can cause tiredness, liver problems, sores in the mouth, and affect blood cells. Regular blood tests are needed to keep an eye on these issues.<br><br>### What to Think About:<br>Doctors start with a high dose of steroids and then slowly reduce the dose while watching how well the treatment is working. Sometimes, other immune system medicines are used to help keep the disease under control without using steroids for too long.<br><br>### Keeping an Eye on Things:<br>Patients with this disease need regular check-ups. These include blood tests to see how the inflammation is doing and how well the organs are working, and sometimes imaging tests like scans to look inside the body. This helps the doctors decide if the treatment is working or if they need to change it.<br><br>### Working Together:<br>Treating IgG4-related disease often needs different kinds of doctors like kidney specialists, stomach doctors, and immune system doctors working together. This helps make sure that all parts of the disease are being treated properly.
65	Kimura Disease	Kimura Disease is a rare chronic inflammatory condition characterized by painless swelling or lumps in the lymph nodes and surrounding tissues, primarily in the head and neck regions. It may also affect the salivary glands and can lead to abnormal increases in eosinophils, a type of white blood cell, and elevated immunoglobulin E (IgE) levels. The exact cause of Kimura Disease is unknown, and its treatment focuses on reducing inflammation and managing symptoms.<br><br>### Medications Commonly Used in Treatment:<br><br>1. **Corticosteroids (e.g., Prednisone)**:<br>- **Mechanism of Action**: Corticosteroids are anti-inflammatory drugs that suppress the immune system broadly. They reduce swelling by decreasing the activity of eosinophils and other inflammatory cells involved in Kimura Disease.<br>- **Potential Side Effects**: Long-term use can lead to side effects such as increased risk of infections, diabetes, osteoporosis, weight gain, hypertension, and mood changes. Because of	Kimura Disease is a rare condition where people get painless lumps mainly around the head and neck area. These lumps are swollen lymph nodes and tissues. The disease also makes a type of white blood cell called eosinophils and a substance called IgE go up higher than normal. Doctors aren't sure why this happens. The treatment helps reduce swelling and control symptoms.<br><br>### Medications Commonly Used in Treatment:<br><br>1. **Corticosteroids (like Prednisone)**:<br>- **What It Does**: These medicines help reduce swelling by calming down the immune system. They are very effective at making the swelling go down and are usually the first treatment used.<br>- **Possible Side Effects**: If used for a long time, they can cause problems like easier infections, higher blood sugar, weak bones, weight gain, high blood pressure, and mood changes. Doctors try to use them for the shortest time possible to avoid

		these risks, doctors often try to use the lowest effective dose for the shortest period possible.<br><br>2. **Cyclosporine**:<br>- **Mechanism of Action**: Cyclosporine is an immunosuppressive medication that specifically inhibits T-cells, a type of lymphocyte involved in the immune response. It is used when steroids are not effective or to reduce the steroid dosage needed.<br>- **Potential Side Effects**: Cyclosporine can cause kidney damage, increased blood pressure, gum hypertrophy, and increased hair growth. Regular monitoring of kidney function and blood pressure is required during treatment.<br><br>3. **Cyclophosphamide**:<br>- **Mechanism of Action**: Cyclophosphamide is a cytotoxic drug that suppresses the immune system by damaging DNA in rapidly dividing cells, including those active in inflammatory processes.<br>- **Potential Side Effects**: This drug can cause bone marrow suppression (leading to decreased blood cells), increased risk of infections, potential bladder toxicity (hemorrhagic cystitis), and increased risk of secondary cancers.<br><br>4. **Mycophenolate mofetil**:<br>- **Mechanism of Action**: Mycophenolate mofetil inhibits the proliferation of B and T lymphocytes, thus reducing the immune response and inflammation.<br>- **Potential Side Effects**: Common side effects include gastrointestinal disturbances, potential liver dysfunction, and a higher risk of infections.<br><br>### Additional Treatment Options:<br>- **Surgical Excision**: For localized lesions, surgical removal of the affected lymph nodes or tissue can be effective but may not always prevent recurrence.<br>- **Radiation Therapy**: In some cases, especially for those not responding well to medications, low-dose radiation therapy has been used to control the growth of lymphoid tissues.<br><br>### Monitoring and Adjustments:<br>Patients with Kimura Disease need regular follow-up to monitor the effectiveness of therapy and manage side effects. Blood tests, imaging studies, and physical examinations are essential to adjust treatments and ensure the disease is being managed effectively.<br><br>### Considerations:<br>Given the rarity of Kimura Disease, treatment is often tailored to individual cases, with a focus on managing symptoms and preventing complications. Collaboration among specialists, including dermatologists, rheumatologists, and oncologists, may be necessary to provide comprehensive care.	these issues.<br><br>2. **Cyclosporine**:<br>- **What It Does**: This medicine helps control the immune system by focusing on certain white blood cells called T-cells that can cause inflammation. It's used when steroids alone don't work well enough or to help use less steroids.<br>- **Possible Side Effects**: Cyclosporine can cause kidney problems, higher blood pressure, gums that grow over the teeth, and extra hair growth. Doctors will check your kidney function and blood pressure often if you're taking this medicine.<br><br>3. **Cyclophosphamide**:<br>- **What It Does**: This is a strong medicine that attacks fast-growing cells, including those that cause inflammation in Kimura Disease.<br>- **Possible Side Effects**: It can lead to fewer blood cells (which can make infections more likely), bladder problems, and a higher chance of other cancers later on.<br><br>4. **Mycophenolate mofetil**:<br>- **What It Does**: This medicine also controls the immune system by slowing down the growth of certain immune cells, which helps reduce swelling and symptoms.<br>- **Possible Side Effects**: It might cause stomach problems, liver issues, and make it easier to get infections.<br><br>### Additional Treatment Options:<br>- **Surgical Excision**: Sometimes, doctors can remove the swollen lymph nodes or tissues with surgery to get rid of the lumps.<br>- **Radiation Therapy**: For tough cases, a low dose of radiation might be used to help control the growth of the lumpy tissues.<br><br>### Keeping an Eye on Things:<br>Patients with Kimura Disease need regular check-ups to see how well the treatments are working and to make sure the side effects are managed. This includes blood tests, scans, and physical exams.<br><br>### What to Think About:<br>Each person's treatment might be different because the disease is so rare. It often involves different types of doctors working together to figure out the best way to manage the disease and keep you feeling as good as possible.
66	Fabry Disease	Fabry disease is a genetic disorder caused by the buildup of a specific type of fat called globotriaosylceramide (Gb3 or GL-3) due to a deficiency in the enzyme alpha-galactosidase A. This buildup can damage organs and tissues throughout the body, including the kidneys, heart, and nervous system. Treatment focuses on replacing the missing enzyme and managing symptoms.<br><br>### Medications Commonly Used in Treatment:<br><br>1. **Enzyme Replacement Therapy (ERT)**:<br>- **Examples**: Agalsidase beta (Fabrazyme) and Agalsidase alfa (Replagal).<br>- **Mechanism of Action**: These medications provide the enzyme that people with Fabry	Fabry disease is a rare genetic disorder where the body can't break down a specific type of fat called globotriaosylceramide (Gb3 or GL-3) because it lacks a necessary enzyme. This fat buildup can harm organs like the kidneys, heart, and nerves. The main treatments help replace the missing enzyme and manage the symptoms to keep the body healthy.<br><br>### Medications Commonly Used in Treatment:<br><br>1. **Enzyme Replacement Therapy (ERT)**:<br>- **Examples**: Agalsidase beta (Fabrazyme) and Agalsidase alfa (Replagal).<br>- **What It Does**: These medications give the body the enzyme it's missing. This

		disease are missing. They help break down the accumulated Gb3, preventing further buildup and reducing damage to organs.<br>- **Potential Side Effects**: Common side effects include reactions to the infusion, such as fever, chills, nausea, and flushing. Some patients may develop antibodies to the enzyme, which can reduce the effectiveness of the treatment.<br><br>2. **Chaperone Therapy**:<br>- **Example**: Migalastat (Galafold).<br>- **Mechanism of Action**: Migalastat works differently from enzyme replacement by stabilizing the body's own dysfunctional enzyme so that it can break down the Gb3 more effectively. It is specifically effective in patients with certain genetic mutations.<br>- **Potential Side Effects**: Side effects are generally mild, including headache, nasopharyngitis (cold-like symptoms), and urinary tract infections.<br><br>### Additional Treatments:<br>- **Pain Management**: Due to the chronic pain that can occur with Fabry disease, medications like gabapentin or carbamazepine may be used to manage neuropathic pain.<br>- **Kidney Protection**: ACE inhibitors or ARBs (angiotensin receptor blockers) may be prescribed to protect the kidneys from damage caused by proteinuria (protein in urine).<br>- **Cardiovascular Care**: Medications to manage heart disease, including beta-blockers, diuretics, or calcium channel blockers, may be needed due to the risk of heart complications in advanced Fabry disease.<br><br>### Monitoring and Adjustments:<br>Regular monitoring is critical to assess the effectiveness of the treatment and adjust as necessary based on enzyme levels, organ function, and overall health. Patients typically undergo routine blood tests, kidney function tests, and heart assessments, along with evaluations for pain and gastrointestinal health.<br><br>### Considerations:<br>The choice of treatment depends largely on the specific mutation present in the patient, the organs affected, and the severity of symptoms. Genetic counseling is often recommended for patients and their families to understand the inheritance pattern and the risks for future generations.<br><br>Managing Fabry disease requires a comprehensive approach tailored to each patient's specific needs and symptoms, often involving a team of specialists including geneticists, nephrologists, cardiologists, and pain management experts.	helps break down the fat that's building up and stops it from causing more damage to organs.<br>- **Possible Side Effects**: Some people might feel sick during the infusion (when the medicine is given into a vein), like getting a fever, feeling cold, or having stomach aches. Sometimes, the body might react to the new enzyme, making the treatment less effective.<br><br>2. **Chaperone Therapy**:<br>- **Example**: Migalastat (Galafold).<br>- **What It Does**: This medicine works by helping the body's own enzyme work better to break down the fat. It's especially good for people with certain types of genetic changes.<br>- **Possible Side Effects**: The side effects are usually mild and can include headaches, cold symptoms, or urinary tract infections.<br><br>### Additional Treatments:<br>- **Pain Management**: Medicines like gabapentin or carbamazepine might be used to help with nerve pain that can happen in people with Fabry disease.<br>- **Kidney Protection**: Doctors may give medicines called ACE inhibitors or ARBs to help protect the kidneys from damage caused by protein in the urine.<br>- **Heart Care**: Because Fabry disease can also affect the heart, some patients might need heart medicines like beta-blockers or calcium channel blockers to help keep the heart working well.<br><br>### Keeping an Eye on Things:<br>Doctors will regularly check how well the treatments are working and how the organs are doing. This includes blood tests, kidney checks, and heart checks. They will make sure the medicines are helping and not causing any problems.<br><br>### What to Think About:<br>The type of treatment used can depend on the person's specific genetic makeup, which parts of the body are affected, and how severe the symptoms are. Sometimes, genetic counselors will work with families to help them understand the disease and its effects on future generations.<br><br>Treating Fabry disease means taking a detailed and personalized approach, often involving a team of different doctors like genetic specialists, kidney doctors, heart doctors, and pain specialists to make sure all parts of the disease are managed well.
67	von Gierke Disease (Glycogen Storage Disease)	Von Gierke Disease, also known as Glycogen Storage Disease Type I (GSD I), is a genetic disorder caused by the deficiency of the enzyme glucose-6-phosphatase. This enzyme is crucial for converting glycogen (stored glucose) into free glucose that the body can use. As a result, individuals with this condition have difficulty maintaining normal blood sugar levels, particularly during fasting periods. They often accumulate glycogen and fat in the liver and kidneys, which can lead to organ enlargement and dysfunction, including renal (kidney) involvement.<br><br>### Medications Commonly Used in Treatment:<br><br>**1. Cornstarch**:<br>- **Mechanism of Action**: Uncooked cornstarch is a slow-release form of glucose that helps maintain stable blood glucose levels, especially overnight or between meals. By providing a steady supply of glucose, it reduces the body's need to break down stored	Von Gierke Disease, or Glycogen Storage Disease Type I (GSD I), is a genetic condition where the body can't properly turn stored sugar (glycogen) into glucose (sugar) that it can use. This causes low blood sugar and stores too much glycogen and fat in places like the liver and kidneys, which can make these organs not work well.<br><br>### Medications Commonly Used in Treatment:<br><br>**1. Cornstarch**:<br>- **What It Does**: Cornstarch acts like a slow-release sugar that helps keep blood sugar levels stable, especially during the night or between meals. This helps prevent the body from running out of sugar.<br>- **Possible Side Effects**: If not used correctly, it can lead to either too low or too high blood sugar levels.

		glycogen.<br>- **Potential Side Effects**: If not timed or dosed correctly, it can lead to hypoglycemia (low blood sugar) or contribute to hyperglycemia (high blood sugar) if taken in excess.<br><br>**2. Allopurinol**:<br>- **Mechanism of Action**: In GSD I, the increased breakdown of glycogen and other metabolic disturbances can lead to the overproduction of uric acid, which may contribute to kidney damage. Allopurinol reduces the production of uric acid by inhibiting the enzyme xanthine oxidase.<br>- **Potential Side Effects**: Rash, gastrointestinal upset, and rarely, more severe reactions such as Stevens-Johnson syndrome or toxic epidermal necrolysis. Long-term use requires monitoring of liver and kidney function.<br><br>**3. ACE Inhibitors (e.g., Lisinopril, Enalapril)**:<br>- **Mechanism of Action**: These drugs are used to manage hypertension and protect the kidneys from further damage by reducing the pressure within the glomeruli (the kidney's filtering units).<br>- **Potential Side Effects**: Cough, elevated blood potassium levels, low blood pressure, and in rare cases, kidney dysfunction and swelling of tissues (angioedema).<br><br>### Dietary Management:<br>- **Controlled intake of fructose and galactose**: Patients are advised to avoid foods containing fructose and galactose, which they cannot properly metabolize.<br>- **Frequent meals with carbohydrates**: To prevent hypoglycemia, regular carbohydrate intake through frequent meals is crucial.<br><br>### Additional Considerations:<br>- **Liver transplantation**: In some severe cases, a liver transplant may be considered to replace the deficient enzyme, especially when there are significant complications or a high risk of liver cancer (hepatocellular carcinoma).<br>- **Regular monitoring**: Continuous monitoring of blood sugar levels, kidney function, liver function, and lipid levels is essential to manage the disease effectively and adjust the treatment plan as needed.<br><br>### Monitoring and Adjustments:<br>Patients with von Gierke Disease require lifelong management and monitoring to ensure adequate glucose levels and to prevent complications. The treatment strategy may evolve as new therapies become available and as the patient ages.<br><br>Managing von Gierke Disease, especially with renal involvement, requires a multidisciplinary approach, often involving geneticists, gastroenterologists, nephrologists, and nutritionists to address the various aspects of the disease and provide comprehensive care.	**2. Allopurinol**:<br>- **What It Does**: Since breaking down too much glycogen can make too much uric acid, which can hurt the kidneys, allopurinol helps lower uric acid levels by blocking the chemical that makes it.<br>- **Possible Side Effects**: It can cause a rash, upset stomach, and very rarely, serious skin reactions. It's important to check how the liver and kidneys are working if taking this medicine for a long time.<br><br>**3. ACE Inhibitors (like Lisinopril, Enalapril)**:<br>- **What It Does**: These medicines help lower blood pressure and can protect the kidneys by easing the pressure in tiny filters inside the kidneys.<br>- **Possible Side Effects**: They might cause a cough, higher potassium levels, low blood pressure, and sometimes affect how well the kidneys work.<br><br>### Dietary Management:<br>- **Avoiding certain sugars**: Patients should not eat foods that have fructose or galactose, which are sugars they can't handle well.<br>- **Eating regularly**: Eating meals with carbohydrates regularly throughout the day helps keep blood sugar stable.<br><br>### Additional Considerations:<br>- **Liver transplant**: In very serious cases, if the liver is really affected, a liver transplant might be considered.<br>- **Regular check-ups**: It's crucial to keep checking blood sugar levels, how well the kidneys and liver are working, and cholesterol levels.<br><br>### Keeping an Eye on Things:<br>Kids with von Gierke Disease need to be checked regularly to make sure their treatments are working well and to adjust as needed. This might include changing how much medicine they take or changing their diet.<br><br>Treating von Gierke Disease, especially when the kidneys are involved, requires a team of different doctors and specialists, like genetic doctors, liver doctors, kidney doctors, and diet experts. This team approach helps take care of all parts of the disease.
